# Switching on photoreactivity in Ti_4_-oxo clusters by increasing the size of 1,*n*-alkane diolate bridging ligands

**DOI:** 10.1039/d5sc08522e

**Published:** 2025-11-27

**Authors:** Ashwani Chikara, Alexander R. Veale, Stephen E. Brown, Jack M. Woolley, Frank De Proft, Sebastian D. Pike

**Affiliations:** a Department of Chemistry, University of Warwick CV4 7AL Coventry UK Sebastian.pike@warwick.ac.uk; b Research Group of General Chemistry (ALGC), Vrije Universiteit Brussel (VUB) Brussels Belgium; c Warwick Centre for Ultrafast Spectroscopy, Research Technology Platforms, University of Warwick Coventry UK; d Department of Physics, University of Warwick CV4 7AL Coventry UK

## Abstract

A series of new isostructural Ti-oxo clusters containing bridging bidentate 1,*n*-alkane diolate ligands with the formula [Ti_4_O_4_(O_2_PR_2_)_4_{O(CH_2_)_*n*_O}_2_] (R = Ph, *n* = 2–5; R = Cy, *n* = 2–4) were prepared by an alkoxide exchange strategy. The cluster with the 1,5-pentane diolate ligand undergoes productive photoredox chemistry in solution under UV light, resulting in the oxidation of one end of the alkane diolate, and subsequent cyclisation into the lactone tetrahydro-2*H*-pyran-2-ol, along with formation of a two-electron reduced Ti-oxo cluster stabilised by pyridine. Clusters with smaller bridging alkane diolates show no productive photoredox reactivity, except for R = Cy, *n* = 3, in which the photoredox products are unstable to further redox processes. Ultrafast electronic absorption spectroscopy studies reveal that all clusters undergo a similar initial photoexcitation step, therefore, productive redox pathways are controlled by the availability of a suitable transition state for rapid proton-coupled electron transfer from the initially generated pendant alkane diolate radicals {Ti–O(CH_2_)_*n*_O˙}. This is dependent on the flexibility (*i.e.* size) of the 1,*n*-alkane diolate ligand backbone. Interestingly, the productive photoredox pathway of the 1,5-pentane diolate cluster is turned off when the flexibility of the cluster is restricted in the single crystal phase.

## Abstract 2

UV light promotes the oxidation of one end of a bridging bidentate 1,5-diolate ligand on a Ti-oxo cluster. In contrast, due to a lack of conformational flexibility, smaller diolates cannot undergo the same photoreaction mechanism.

## Graphical abstract

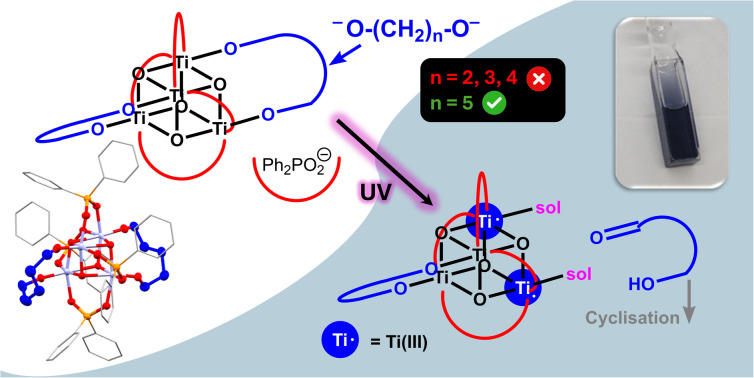

## Introduction

TiO_2_ is one of the most studied photoactive metal oxide materials, with a wide range of applications based on photocatalysis including self-cleaning materials, and for improving urban air quality.^1–3^ It remains a key material for cutting-edge applications and fundamental studies.^4,5^ Furthermore, titanium is very Earth abundant, and can be used sustainably, providing an impetus for further research on photoactive Ti-based materials, including nanostructures and metal–organic-frameworks (MOFs).^6–12^ Molecularly sized and precisely defined titanium-oxo clusters offer an opportunity to study the photoreactivity of titanium oxides at the atomic level. In recent years, a wide variety of titanium-oxo clusters have been studied, including as models for TiO_2_.^13–17^ Titanium-oxo clusters are highly tuneable, allowing for interaction with a wide range of surface organic ligands, which can moderate their electronic structure and photochemical properties.^18–20^ This tuneability allows for the design of clusters to probe photoreaction mechanism and to mimic oxide materials with certain surface functionality.^21–23^

**Fig. 1:**
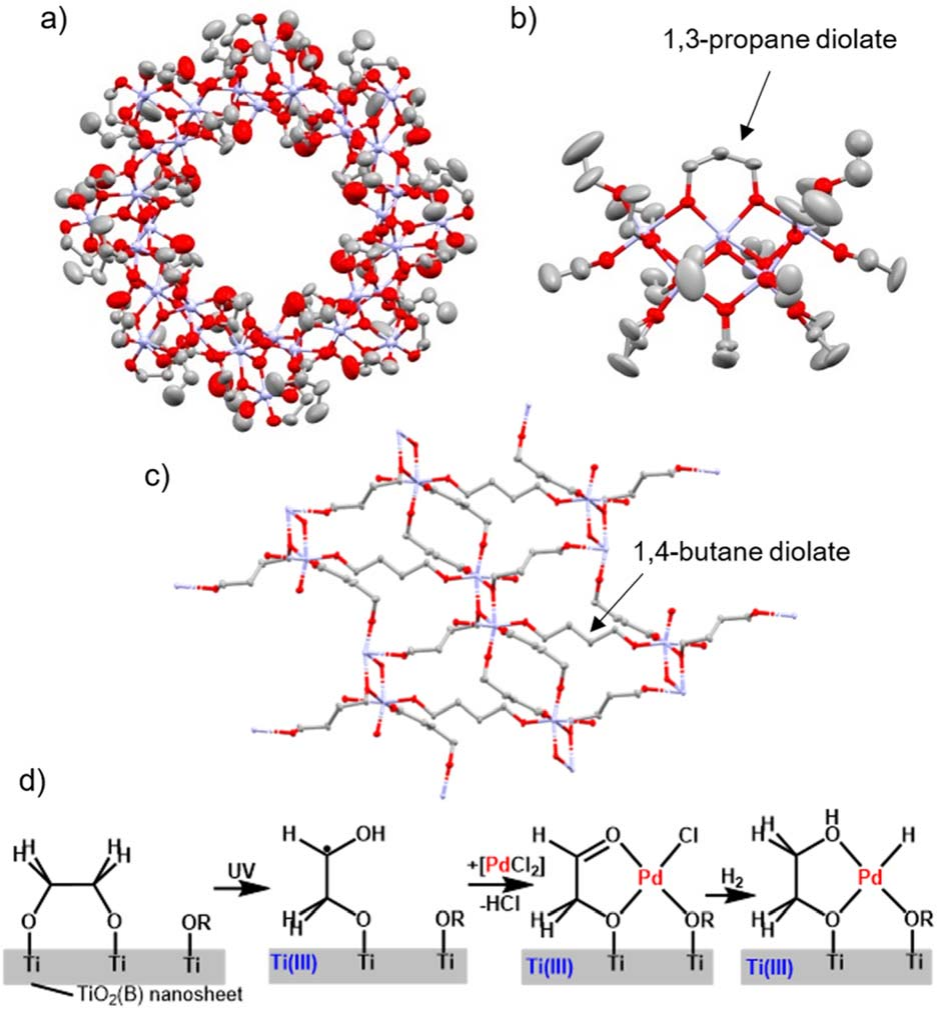
Titanium-oxo clusters, titanium-MOF and TiO_2_ nanosheets supported by alkane diolate ligands: (a) [Ti_32_O_16_(OCH_2_CH_2_O)_32_(EtCOO)_16_(OCH_2_CH_2_OH)_16_]; (b) [Ti_7_O_4_(O(CH_2_)_3_O)(OEt)_18_]; (c) [Ti_2_(O(CH_2_)_4_O)_3_(O(CH_2_)_4_OH)_2_]; (d) TiO_2_(B) nanosheet supported by 1,2-ethane diolate and it's subsequent photochemical and hydrogenation reactivity with a Pd precursor.

Multidentate bridging organic ligands, including aliphatic alkane di/triolates as well as catechols and calixarenes, have been utilised in a wide variety of metal-oxo cluster structures, with the binding arrangement of the ligand often affecting the shape of the resulting cluster.^13,24–29^ Ethane diolate (–OCH_2_CH_2_O–) has been used as a ligand to support doughnut-shaped Ti_32_-oxo clusters, which crystallise into a micro-porous material, evidencing the capacity of small bidentate bridging alkane diolates to enable the formation of large and complex shapes (Fig. 1a).^30,31^ Short diols, such as 1,3-propanediol, have been shown to exchange for two mono-alkoxide groups on neighbouring sites within a Ti-oxo cluster (Fig. 1b).^32^ Larger alkane diolate ligands with flexible backbones have the capacity to act as linkage ligands, and are typically found in this configuration. For example, the reaction of Ti(O^i^Pr)_4_ and excess 1,4-butanediol produces a MOF with the formula of [Ti_2_(O(CH_2_)_4_O)_3_(O(CH_2_)_4_OH)_2_]_∞_ where the butane diolate ligands act as linkers between Ti_2_ units (Fig. 1c), in contrast, use of 2*R*,3*R*-2,3-butanediol produces molecular clusters, [Ti_3_(O(CHMe)_2_O)_4_(O(CHMe)_2_OH)_4_], with alkane diolates bound in a bridging coordination mode.^33^ 1,5-Pentane diolate has also recently been used as a linker in a Ti-MOF.^34^

1,2-Ethane diolate is also a very useful ligand for producing ultrathin nanosheets of TiO_2_. In this process, TiCl_3_ (or TiCl_4_)^35^ reacts with ethylene glycol and upon heating to 140–180 °C forms 1.1 nm thick nanosheets of TiO_2_(B), an unusual polymorph of TiO_2_.^36^ These nanosheets have found uses for reversible lithium storage, surfaces for surface-enhanced infrared absorption spectroscopy, and as photocatalysts for the reduction of CO_2_ to alcohols.^35,37,38^ With a surface coverage of 1,2-ethane diolate ligands, these sheets can be photoactivated, reported to generate Ti(iii) sites and also 1,2-ethane diolate radicals 
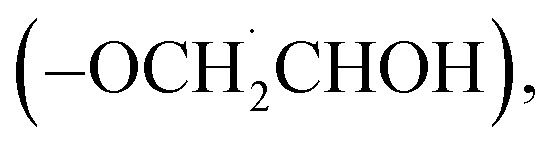
 which are stable under inert conditions once photogenerated.^22^ This photoactivated form can react with [PdCl_4_]^2−^, where PdCl_2_ units coordinate to the 1,2-ethane diolate radicals, with subsequent release of HCl. This process results in the formation of atomically dispersed Pd on a TiO_2_ support, which was highly active in hydrogenation catalysis (Fig. 1d).^22^ The importance of these photoactive TiO_2_ structures with surface diolates is a motivation for the detailed photochemical study of well-defined titanium-oxo clusters with these ligands.

**Fig. 2:**
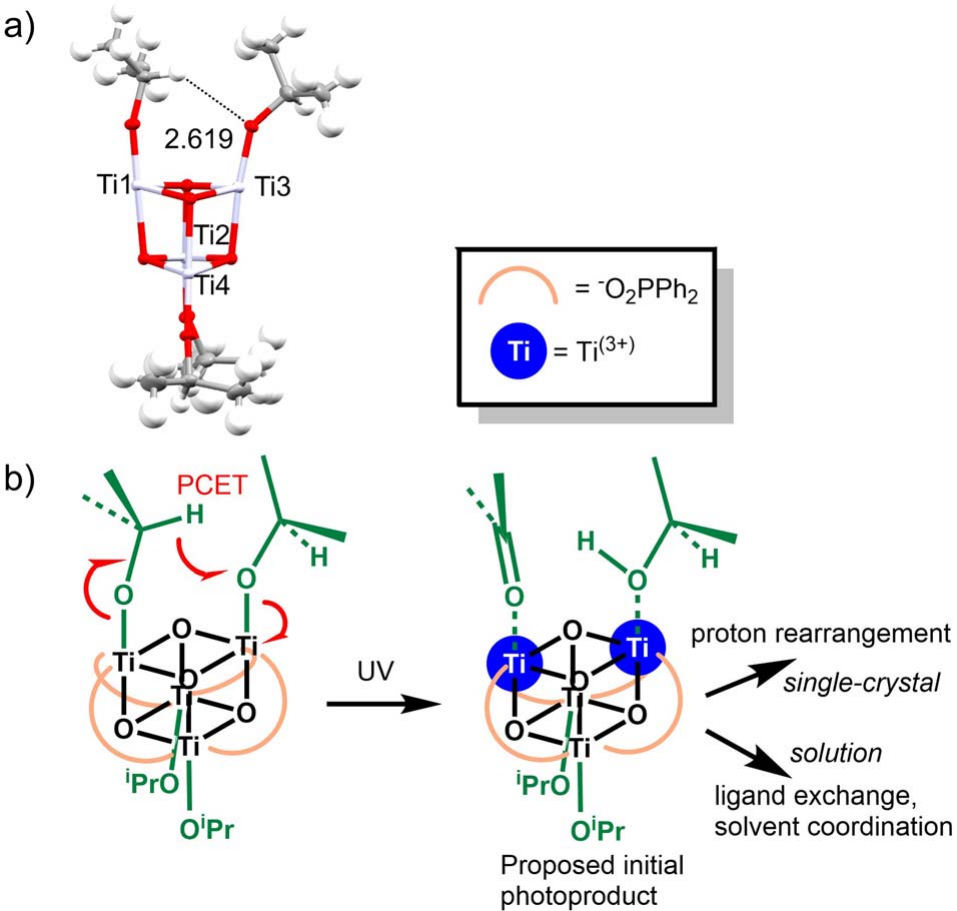
(a) Single-crystal structure of 1^Ph^ showing a close O–C–H⋯O distance between adjacent alkoxide ligands. Phosphinate ligands are omitted for clarity, displacement ellipsoids shown at 50%. (b) Proposed photochemical reaction pathway under UV light resulting in the formation of ketone and alcohol products.^23^

Our research team has previously reported on the photoredox reactivity of small Ti-oxo clusters [{TiO(O^i^Pr)(L)}_*n*_] (L = O_2_PPh_2_, *n* = 4 (1^Ph^); L = O_2_PCy_2_, *n* = 4 (1^Cy^); L = O_2_C^*t*^Bu, *n* = 6) with (mono)alkoxide ligands.^21,23,39^ In these studies photoactivation using UV light results in the formation of one equivalent of an (oxidised) ketone (or aldehyde for primary alkoxides), one equivalent of free alcohol, and a two-electron reduced Ti-oxo cluster which is stabilised by solvent interactions. This overall two-electron process is assigned to an intramolecular reaction, in which the ketone and alcohol are formed from the same cluster. Experiments suggest that generation of free alkoxide radicals is not a major reaction pathway, which may be important in selective photoredox catalysis.^21^ Studies of this reaction in the single-crystal phase indicate that site-selective photoredox reactivity occurs when a close interaction between two adjacent Ti–O^i^Pr (with a short OCMe**H**⋯**O**^i^Pr distance) enables an efficient proton-coupled electron transfer (PCET) step between the two alkoxide groups to generate the organic products (Fig. 2).^23^ Solution-based studies have shown the importance of solvents on the rate of photoreactivity, indicating that coordinating and protic solvent molecules help facilitate the photoreaction process, and ultimately coordinate to the Ti atoms in the reduced cluster.^21,39^ The photoreduced clusters can be easily re-oxidised with air, enabling a catalytic process for alcohol oxidation using Earth-abundant metals, air and light.^21^

With recent interest in upgrading plastic waste, *via* ethylene glycol intermediates, there is interest in selective oxidation processes for diols.^40^ We predicted that in the case of a 1,*n*-alkane diolate ligand, acting in a bridging bidentate manner, coordinated to adjacent Ti sites on one face of a cluster similar to 1^Ph^, the photoredox reaction could generate one equivalent of the corresponding *n*-hydroxyaldehyde directly, but only if the important PCET step was stereochemically accessible. This may provide structural design criteria for Ti/O materials suitable for selective photoredox reactivity. A further objective is to understand the prevalence of free radical species in the photoredox pathways of diolate ligands,^22^ and whether their reduced flexibility, relative to a monodentate alkoxide, causes differing photoreaction intermediates. In this article the photochemistry of Ti_4_-oxo-phosphinate clusters with 1,*n*-alkane diolate ligands is explored, uncovering the photoredox reactivity at the atomic level, and providing enhanced understanding of multi-electron intramolecular photoredox pathways in Ti-oxo clusters.

## Results and discussion

### Alkoxide exchange

Previous studies have reported that alkoxide exchange processes can occur in Ti-oxo clusters with retention of the core structure, for example, the replacement of O^i^Pr groups on 1^Ph^ with O^*t*^Bu, OEt or 4-pentene-1-oxide.^21,39^ Typically, alkoxide exchange occurs at elevated temperatures (70 °C), using an excess of the reacting alcohol to facilitate full exchange. The phosphinate ligands in 1^Ph^ or 1^Cy^ make for useful ^31^P NMR spectroscopic handles to follow these exchange processes. To prepare clusters with a alkane diolate ligand, 1^Ph^ or 1^Cy^ was reacted with two equiv. of diols (1,*n*-HO(CH_2_)_*n*_OH, *n* = 2, 3, 4, 5) in toluene at 70 °C for 4–6 days, releasing four equivalents of isopropanol and cleanly forming [Ti_4_(µ_3_-O_4_)(µ_2_-O_2_PR_2_)_4_(µ_2_-{κ^1^O(CH_2_)_*n*_κ^1^O})_2_] (R = Ph or Cy; *n* = 2, 2^Ph^ or 2^Cy^; *n* = 3, 3^Ph^ or 3^Cy^; *n* = 4, 4^Ph^ or 4^Cy^; *n* = 5, 5^Ph^, Fig. S1–S21). The products of these reactions are favoured entropically by the chelate effect (the incoming bidentate bridging ligand displaces two monodentate ligands), and their reduced solubility in toluene and/or removal of isopropanol by vacuum during workup.

**Scheme 1:**
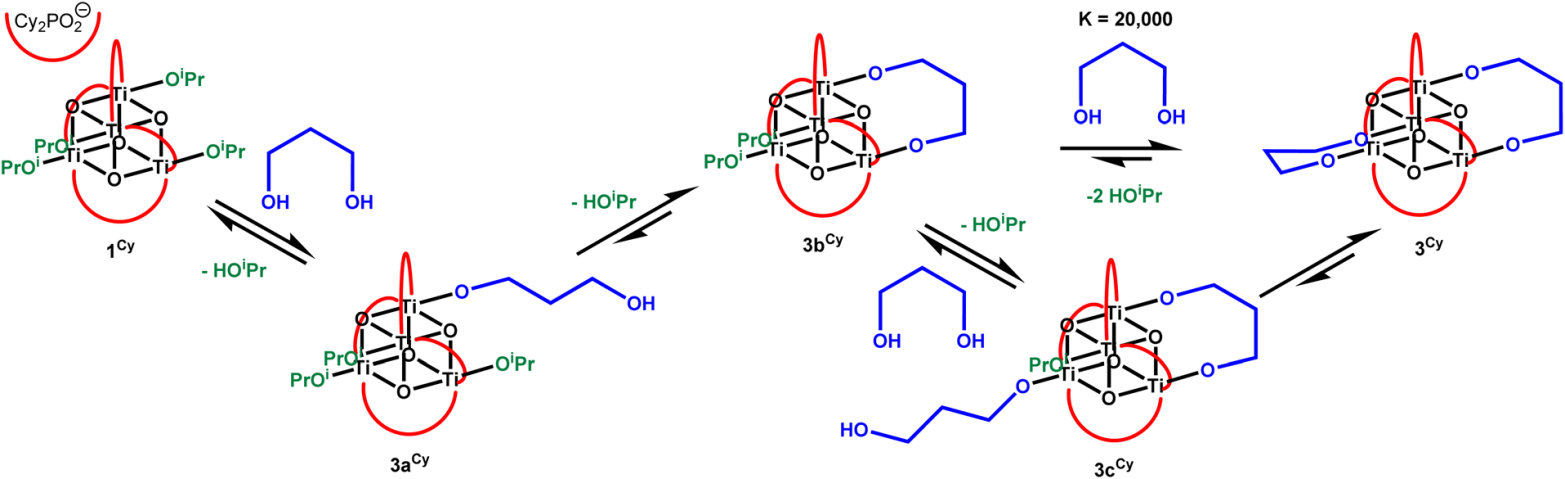
Steps of the alkoxide exchange process for the reaction of 1^Cy^ with 1,3-propanediol.

*In situ* ^1^H and ^31^P{^1^H} NMR analysis, supported by electrospray ionisation mass spectrometry (ESI-MS) studies, suggests that the alkoxide exchange occurs *via* intermediate species, with the diol initially adopting a mono-coordinated mode (*e.g.*3a^Cy^, [Ti_4_O_4_(O_2_PCy_2_)_4_(OR^1^)_3_(OR^2^)] (OR^1^ = O^i^Pr; OR^2^ = O(CH_2_)_3_–OH), Scheme 1, Fig. S22 and S23) before displacing a second isopropoxide to complete the bidentate bridging mode on one face of the cluster (*e.g.*3b^Cy^, [Ti_4_O_4_(O_2_PCy_2_)_4_(O^i^Pr)_2_{O(CH_2_)_3_O}] Scheme 1). Further alkoxide exchange proceeds *via*3c^Cy^, [Ti_4_O_4_(O_2_PCy_2_)_4_(O^i^Pr)µ-{O(CH_2_)_3_O}{κ^1^O(CH_2_)_3_OH}] (Fig. S27), and a final ring closing protonolysis completes the process.

3b^Cy^ can also be prepared by adding an excess of isopropanol to the fully exchanged cluster 3^Cy^. Addition of 30 equiv. of isopropanol to 3^Cy^ slowly generates 3b^Cy^ (Scheme 1 and Fig. S24–S26) with an equilibrium constant, *K*, of around 20 000 (formation of 3^Cy^ + 2 ^i^PrOH from 3b^Cy^ + HO(CH_2_)_3_OH), suggesting the compounds with two bridging diolate ligands are strongly favoured given sufficient opportunity to complete equilibration.

### Crystal structures

**Fig. 3:**
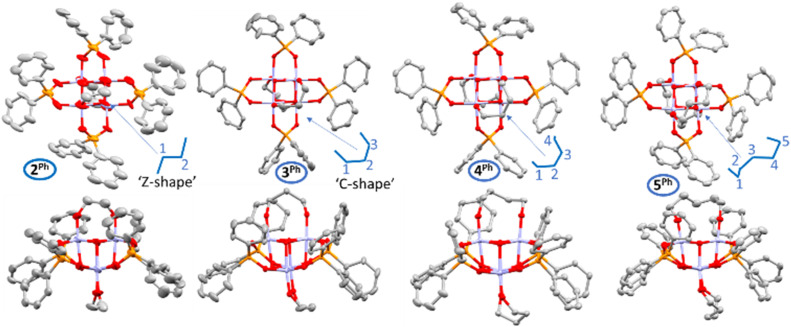
Single crystal solid-state structures of 2^Ph^, 3^Ph^, 4^Ph^, & 5^Ph^ displayed from two different angles, ellipsoids shown with 50% probability. Hydrogen atoms omitted for clarity. For analogous structures of 2^Cy^, 3^Cy^, 4^Cy^ see Fig. S28. For bond lengths and angles, see Tables S1 and S2.

The smaller diols (*n* = 2, 3) in combination with diphenylphosphinate ligands result in the direct precipitation of 2^Ph^ or 3^Ph^ from toluene, in contrast, the products with larger diols (*n* = 4, 5), or when using the dicyclohexylphosphinate ligand, retain solubility (Fig. S21), and these were isolated by concentrating and cooling the solution to facilitate crystallisation. Single crystals suitable for X-ray diffraction can be prepared by cooling toluene solutions (5^Ph^, 4^Ph^, 3^Cy^), recrystallisation from CH_2_Cl_2_ layered with pentane (3^Ph^), or slow evaporation of a CHCl_3_ solution (2^Ph^) or heptane solution (2^Cy^, 4^Cy^). Solid-state structures were collected and are displayed in Fig. 3 and S28. ^31^P and ^1^H NMR spectroscopy and ESI-MS confirm the solid-state structures are retained in solution (Fig. S1–S20). The solid-state structures confirm that each diolate coordinates to two adjacent Ti sites on an exposed Ti_2_O_2_ square face. It is noteworthy that 5^Ph^ is the first crystallographically analysed example of a 1,5-pentane diolate ligand acting in a bidentate manner to the face of a cluster, as opposed to linking between two clusters.

The Ti_4_O_4_ core and coordination of phosphinate ligands remains similar across the series and are closely related to those of starting clusters 1^Cy^ and 1^Ph^, noting a slightly wider variety of bond angles in 5^Ph^ (Tables S1 and S2). However, the binding geometry of the diolate ligand does show systematic variation. In 1^Ph^ the Ti⋯Ti distance on the square face of 1^Ph^ is ∼2.9 Å and the O⋯O distance of adjacent alkoxides is 3.3–3.4 Å, indicating a natural outward puckering of the Ti–O bonds. In the alkane diolate complexes, the O⋯O distances in the bidentate diolate vary from ∼3.1 Å to 3.5 Å across the series. The smallest 1,2-ethane diolate ligand allows an octahedral geometry at Ti, with the ligand forming close to 90° angles (89.8°–91.5°) to the perpendicular Ti⋯Ti vector. This angle increases as the backbone of the alkane diolate increases, reaching a range of values of 96°–102° for 5^Ph^ (which is similar to 1^Ph^) and indicating subtle changes to the coordination geometry at Ti across the series (Table S2 and Fig. S29). In 1,3-propane diolate complexes 3^Cy^ and 3^Ph^, the diolate shows very minor ring strain in this bridging position with expansion of the C–C–C angle to 111.7(2)° in 3^Cy^ and 114.2(4)° and 114.9(3)° in 3^Ph^. Subtle steric differences from the Cy_2_PO_2_ and Ph_2_PO_2_ ligands dictate two different ways for the alkane diolate to coordinate in these structures. In 3^Cy^ the diol coordinates with a planar C–C–C backbone with oxygen atoms projected above and below this plane, to take a ‘Z-shape’, resulting in overall *D*_2_ symmetry for the cluster. In contrast, 3^Ph^ adopts an asymmetrical geometry with the diols coordinating with a ‘C-shape’. The smaller 1,2-ethane diolate ligand adopts the ‘Z-shape’ coordination geometry in both 2^Ph^ and 2^Cy^, whilst the larger 1,*n*-alkane diolate ligands adopt geometries closer to the ‘C-shape’ (Fig. 3 and S28).

### Photochemistry

**Scheme 2:**
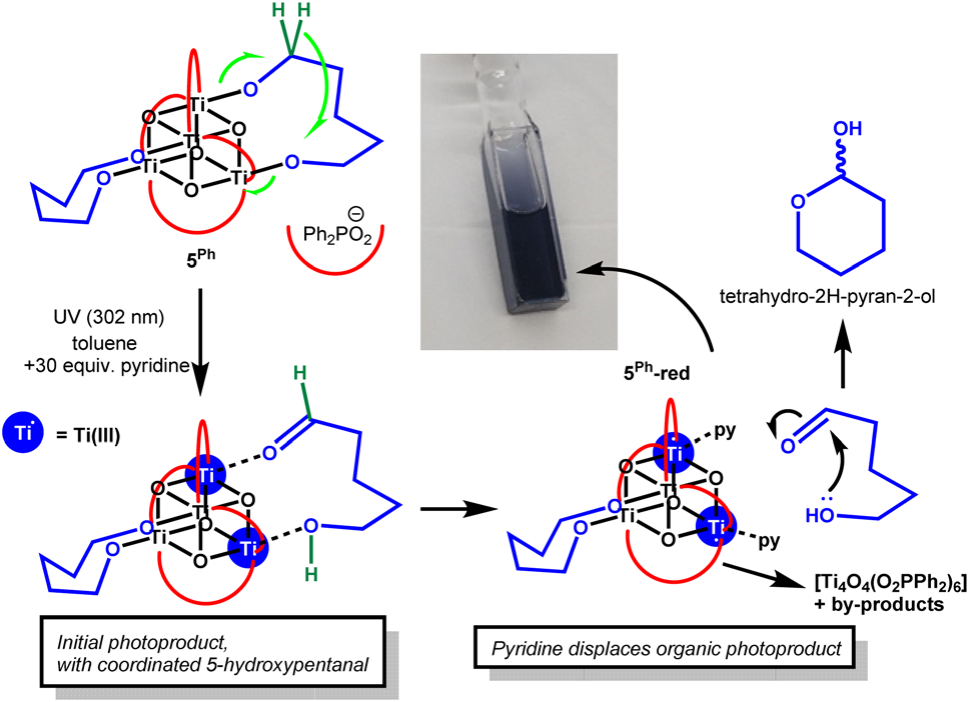
Photoredox reactivity of 5^Ph^ in toluene + 30 equiv. pyridine (green hydrogen atoms highlight H atom transfer in redox process) and subsequent cyclisation of oxidised organic product to its lactol form.

The solution UV absorption spectra of 3^Ph^ and 5^Ph^ were collected and were found to be very similar to 1^Ph^ (onset ∼3.5 eV), likewise the spectrum of 3^Cy^ was almost identical with that of 1^Cy^ (onset ∼3.6 eV, Fig. S30–S33).^39^ This indicates little effect from the inclusion of the alkane diolate on the electronic structure. To study the photoredox reactivity of these clusters, they were dissolved in d^8^-toluene (or CDCl_3_ or CD_2_Cl_2_ for 2^Ph^ and 3^Ph^, which are insoluble in toluene) under inert atmosphere and exposed to 302 or 365 nm UV irradiation (Analytik Jena UVLM-26 EL series UV lamp; 302 nm, 3 mW cm^−2^ @ 1 cm; 365 nm, 1.7 mW cm^−2^ @ 1 cm). Previous studies of the photoreaction of Ti-oxo clusters have indicated that inclusion of coordinating or protic solvents can promote photoredox reactivity,^21,39^ and therefore 30 equivalents of pyridine were added to the solutions.^39^ Compounds 2^Ph^, 3^Ph^ and 4^Ph^ do not undergo any colour change under UV light, and appear inactive towards productive photoredox reactivity (N.B. pyridine and CH_2_Cl_2_ do react under 302 nm light to produce 1,1′-methylenedipyridinium dichloride, see Note S1). In contrast, 5^Ph^ readily transforms to a deep blue colour under 365 or 302 nm irradiation, indicating the reduction of some Ti(iv) sites to Ti(iii) (Scheme 2 and Fig. S34–S38, similar photoreactivity is also observed in CDCl_3_ with pyridine). The rate of consumption of 5^Ph^ is comparable to that of 1^Ph^ under the same conditions.^39^ The ^1^H NMR spectra from these photoreactions indicate the oxidation of approximately one 1,5-pentane diolate ligand per 5^Ph^ into the lactol tetrahydro-2*H*-pyran-2-ol. This occurs *via* a two-electron intramolecular process on one side of the cluster that generates 5-hydroxypentanal, that, in turn, cyclises into the lactol form (Scheme 2). Small traces of a byproduct assigned as (uncoordinated) 4-pentene-1-ol,^21^ which is a dehydrated form of 1,5-pentanediol, are also observed by ^1^H NMR spectroscopy. When the photoreaction is repeated in THF solvent (without additives and with 365 or 302 nm light) significantly more pent-4-ene-1-ol is formed, with less tetrahydro-2*H*-pyran-2-ol (Fig. S39–S41 and Table S3). It appears that in THF the alkene byproduct is favoured, although, considering this is not formed by a formal redox reaction, it is unclear whether this is formed by a photoreaction or in a dark process potentially triggered by protic species (alcohols) that evolve from separate photoredox processes (see Fig. S42 for potential mechanisms). To explore any onward reactivity of the organic photoproducts, 30 equiv. of tetrahydro-2*H*-pyran-2-ol was added to 5^Ph^ (without UV light), no major changes were observed, although evidence of slow alcohol exchange was observed over several days (Fig. S43).

Following photoreaction of 5^Ph^, the resulting doubly reduced cluster, ‘5^Ph^-red’, is expected to be stabilised by coordinating solvent molecules to satisfy the coordination sphere of the Ti sites, and a formula of [Ti_4_O_4_(O_2_PPh_2_)_4_{O(CH_2_)_5_O}(sol)_2_] (sol = pyridine or THF) is suggested for the initial photoproduct(s).^21^ The UV-vis spectrum in toluene/pyridine shows a broad absorption with a maximum at 580 nm (Fig. S44 and S45), similar to photoreduced 1^Ph^, and slightly red-shifted compared to previously reported mixed-valence [Ti_6_O_6_(O^i^Pr)_4_(py)_2_(O_2_C^*t*^Bu)_6_].^21,39^ The strong colour is consistent with metal to ligand charge transfer involving the coordinated pyridine ligands.^39^ Clear NMR signals for 5^Ph^-red are not observed, however, a minor ^31^P signal at 32.3 ppm is observed in THF (Fig. S41). An Evans shift (*µ*_eff_ of ∼1) is observed in the NMR spectra (comparing the chemical shifts to a sealed capillary), consistent with the formation of paramagnetic clusters which are NMR silent (Fig. S46). Previous DFT calculations suggest that two-electron reduced Ti-oxo clusters of this type have very similar energies in triplet or singlet electronic states, possibly existing in different magnetic states depending on environments (*e.g.* coordinating ligands, solvent *etc.*).^21,39^ Bearing this in mind, the *µ*_eff_ calculated (giving an average of ∼0.5 unpaired electrons per cluster) may imply a mixture of photoproducts with differing magnetic properties. Over time, the solution becomes a paler grey/blue colour and dark precipitates or small cubic crystals form (Fig. S45). These observations are identical to those previously noted following photoreduction of 1^Ph^, which undergoes ligand rearrangement to generate crystals of homoleptic mixed-valence [Ti_4_O_4_(O_2_PPh_4_)_6_] (together with unidentified byproducts).^39^ In this process, when the pyridine ligands are displaced from 5-red, the deep blue colour is lost.

**Scheme 3:**
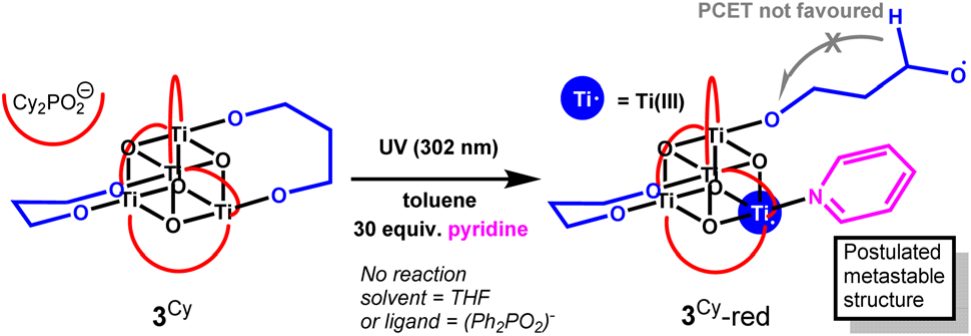
Proposed photoredox reactivity of 3^Cy^ in the presence of pyridine.

In contrast to 3^Ph^, 3^Cy^ does undergo photoreaction to a blue solution (Fig. S45), but at a slower rate than that for 5^Ph^. As expected, based on studies of 1^Cy^,^39^ 302 nm UV light is needed to excite 3^Cy^ and approximately 60% consumption of 3^Cy^ is observed by NMR spectroscopy under these conditions after five hours (Fig. S47). It is noted that 3^Cy^ only undergoes photoreaction in the presence of pyridine and that other activating solvents such as THF are not effective (despite THF being an effective activator for 1^Cy^, Fig. S48). The deep blue colour of the photoproduct (3^Cy^-red) in the presence of pyridine is consistent with 5^Ph^-red (Fig. S49 and S50).^21,39^ No NMR signal for 3^Cy^-red or for any organic photoproduct is observed by NMR spectroscopy, but the dicyclohexylphosphinate signals become broadened and a small Evans shift implies the introduction of paramagnetic photoproducts. The blue colour of the photoirradiated solution fades to a pale brown colour, even under strictly air-free conditions (Fig. S45 and S50), with partial recovery of the NMR signal of 3^Cy^ but no new NMR resonances. We hypothesize that upon photoreaction of 3^Cy^, a Ti–OR bond is homolytically cleaved to give a short-lived diradical state, which only has sufficient lifetime for onward reactivity when a strongly coordinating solvent (pyridine) blocks the reduced Ti site. This results in an unstable species which has a dangling alkoxide radical still attached to the cluster (Scheme 3). This arrangement may not be sufficiently flexible to facilitate PCET to eject 3-hydroxypropanal but may instead lead to other reactivity with the nearby sp^3^ C–H bonds on the dicyclohexylphosphinate ligand, or with solvent, leading to unknown paramagnetic side-products. Spectroscopic studies were not able to identify any intermediate species, due to their sensitive and short-lived nature and inability to produce reasonable quantities of 3^Cy^-red. Testing the photoreaction with 30 equiv. NEt_3_ added as a strong (but bulkier) Lewis base donor resulted in only very slow formation of a pale purple solution, suggesting that pyridine remains a better promoter in this case. These results emphasise that a coordinating solvent molecule can be very important for photochemical processes in these Ti-oxo clusters, and must play a role in the photoreaction mechanism, possibly blocking a charge recombination process to regenerate the starting cluster.

To probe solutions containing 3^Cy^-red, a solution of 3^Cy^ was irradiated with 302 nm light for 60 minutes, and an EPR spectrum of the resulting blue solution was collected after freezing at 150 K (Fig. S51). This reveals a typical signal for a Ti(iii) containing species with *g*_‖_ = 1.96, *g*_⊥_ = 1.86 and Gaussian line broadening of 8.6 G, consistent with a Ti(iii)-based paramagnetic species.^21,23^ However, no further organic radical signals were identified, implying any radical alkoxide forms are short-lived and react with surrounding solvent/ligand. Afterwards, the EPR tube was opened to air, resulting in a rapid colour change to yellow (Fig. S52) and thereafter no EPR signal could be detected, suggesting oxidation to a diamagnetic species. Previous studies have indicated that re-oxidation of two-electron-reduced clusters can result in either (paramagnetic) superoxide or (diamagnetic) peroxide species depending on the cluster structure.^21,39^

2^Cy^ and 4^Cy^ were also investigated, but these compounds do not undergo productive photochemistry under UV light (Fig. S53). Therefore, the photoreactivity of 3^Cy^ appears to be a special case among the other compounds with small bidentate bridging diolates studied, potentially due to the size of the diolate and the proximity of the cyclohexyl groups of the ligand (Fig. S54).

### Transient electronic absorption spectroscopy

**Fig. 4:**
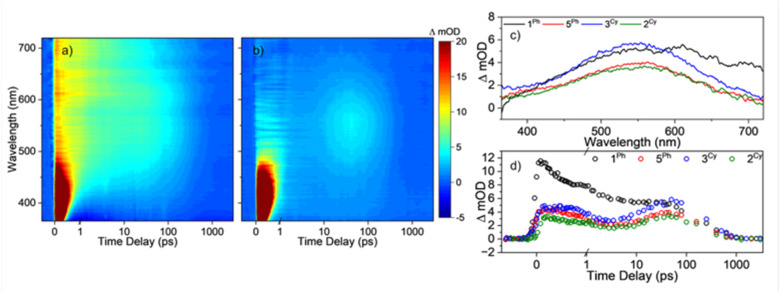
False-colour heatmaps of the transient absorption spectra of (a) 1^Ph^ and (b) 5^Ph^ following photoexcitation at 350 nm. Note that the strong signal <500 nm and <1 ps is from the solvents (Fig. S49). The time delay is plotted linearly up to 1 ps and then logarithmically to the maximum time delay of 3 ns. (c) Transient absorption spectra of the complexes at a pump-probe delay of 40 ps. (d) ΔmOD as a function of time for a probe wavelength of 550 nm for the complexes, the time delay is plotted linearly up to 1 ps and then logarithmically to the maximum time delay of 3 ns.

1^Ph^, 5^Ph^ (which forms stable mixed-valence products under UV light), 3^Cy^ (which gives a short-lived unstable photoreduced species), as well as 2^Cy^ (which does not generate an observable photoproduct), were dissolved in toluene (0.44 mM) with 30 equiv. pyridine and examined by transient electronic absorption spectroscopy (TEAS) over the first 3 ns after photoexcitation (350 nm). Photoexcitation of 1^Ph^ generates a broad absorption across the visible range centred at ∼550 nm that decays after ∼100 ps (Fig. 4a). All samples with alkane diolates show an initial weak absorption that evolves into a stronger broad absorption centred around 550 nm, which again decays after ∼100 ps (Fig. 4b, S55a and b; for solvent absorption see Fig. S56). In all cases the increased absorption in the visible region is consistent with photoreduction of a Ti site giving a coloured Ti(iii) containing species. The broad signal is indicative of intervalence charge transfer between Ti sites.^21,23^ In all cases the excited species decay to negligible levels after 1 ns, implying that the productive formation of stable redox products occurs in low yield, with radical recombination favoured. Considering the similar signals observed at short timescales for all these clusters, regardless of whether they visibly turn blue under UV light over longer timescales, it would appear that the initial photochemical steps, *e.g.* oxygen to metal charge transfer, are similar in all cases, but that rates of excited-state charge recombination and/or onward redox steps may be varied – leading to stable photoproducts in only some cases.

In contrast to the <1 ns lifetimes in toluene/pyridine, when a protic additive (30 equiv. ^i^PrOH) is used with 1^Ph^ instead of pyridine, excited species do not decay over the 3 ns of measurement, indicating a more favourable transformation into the stable photoproducts, and perhaps hinting that protic solvents can help facilitate the important PCET step in the mechanism. This finding is consistent with faster photoreaction rate reported in this solvent mixture (Fig. S55c).^21,39^ N.B. adding ^i^PrOH to 3^Cy^ leads to an equilibrium with 3b^Cy^ – irradiation of this mixture results in consumption of both species, whilst broadly maintaining the equilibrium, with evidence that 3b^Cy^ generates acetone from preferential oxidation of an O^i^Pr group over the alkane diolate species (Fig. S57 and S58).

### DFT analysis

**Fig. 5:**
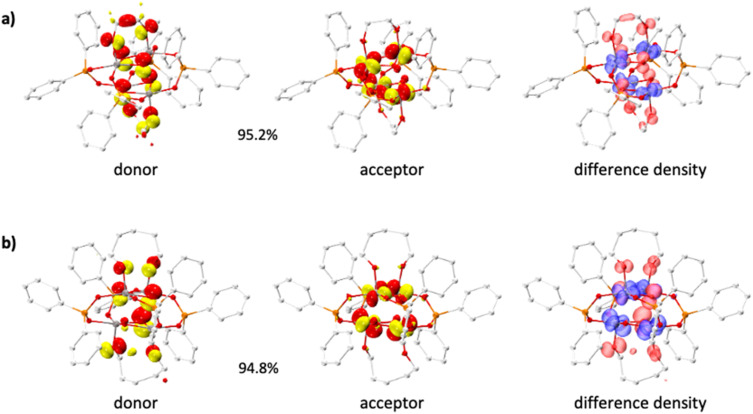
Dominant natural transition orbital (NTO) pairs (isosurface value = ±0.05 au) and electron-difference-density maps (isosurface value = ±0.003 au) for the lowest excitation of (a) 2^Ph^ and (b) 5^Ph^ at the PBE/TZVP//PBE/def2-SVP level of theory. Percentages indicate NTO pair contributions. In difference-density plots, red indicates electron depletion and blue indicates accumulation of electrons during excitation.

Considering the different photoreactivity observed with 5^Ph^ compared to the other compounds, the electronic structure of 5^Ph^ was probed using DFT calculations and compared to that of (photoinactive) 2^Ph^. As expected, and in line with reported calculations for 1^Ph^,^39^ the HOMO of these compounds is comprised of oxygen atoms on the Ti_4_O_4_ core and alkoxide oxygens, with some contribution from diphenylphosphinate π orbitals (Fig. S59 and S60). In 2^Ph^ there is also a contribution from the carbons in the OCH_2_CH_2_O ethane diolate fragment to the HOMO. The LUMO states are comprised of Ti 3d orbitals (Fig. S59 and S60). Time-dependent density functional theory (TDDFT) calculations and natural transition orbital (NTO) analysis were performed to investigate the nature of the lowest-energy excitations in 2^Ph^ and 5^Ph^. In both cases, the excitation is dominated by a single NTO pair (>90% contribution), confirming a well-defined transition. The hole orbital is primarily localised on the oxo and alkane diolate oxygens; in 2^Ph^, there is also a minor but distinct contribution from the C–C bonding orbital of the ethane diolate backbone. The acceptor orbital is centred on the Ti atoms in both complexes, consistent with a ligand-to-metal charge transfer (LMCT) process. The corresponding electron-density difference maps further support this agreement, showing charge depletion from the ligand framework and accumulation on the titanium centres (Fig. 5). These results align with the transient absorption features observed experimentally, particularly the broad absorption at ∼550 nm, characteristic of transient Ti(iii) formation. The similarity in LMCT character of the initial excitations in 2^Ph^ and 5^Ph^ suggests that the differences in their photochemical behaviour do not arise from the nature of the excited-state electronic structure, but rather from differences in excited state relaxation dynamics or downstream chemical reactivity.

### Mechanistic discussion

**Scheme 4:**
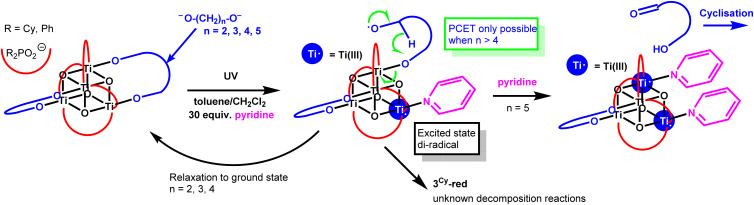
Proposed mechanism for photoactivation of Ti-oxo clusters with bridging alkane diolate ligands.

The photoreaction of 5^Ph^ to produce 1 equiv. of tetrahydro-2*H*-pyran-2-ol, provides further evidence for the photoactivation of small Ti-oxo-alkoxide clusters to occur *via* a two-electron mechanism in which an aldehyde/ketone and alcohol product can be released from the same face of the cluster without accessing long-lived radical intermediate states. This is important for considering reaction selectivity in photocatalysis based on Earth-abundant first-row transition metals, which would typically direct free-radical based photoprocesses if only a single metal centre undergoing ±1 oxidation-state is involved. By decreasing the size of the 1,*n*-alkane diolate ligand, this photoreaction is essentially stopped. The lack of productive reactivity is best explained by a short-lived intermediate (diradical) excited state, which preferentially relaxes back to the ground state if a suitable geometry for PCET to the other end of the diolate is not available (Scheme 4). Whilst 3^Ph^ does not undergo photoredox reaction, in the case of the related 3^Cy^, with a subtly different steric environment and an aliphatic substituent on the ligand, the excited-state diradical may survive long enough to react with its surroundings, but only if pyridine is present, perhaps *via* a PCET from a proximal cyclohexyl group (noting that sp^3^ C–H bonds in 3^Cy^ are more easily broken than sp^2^ C–H bonds in 3^Ph^).

**Fig. 6:**
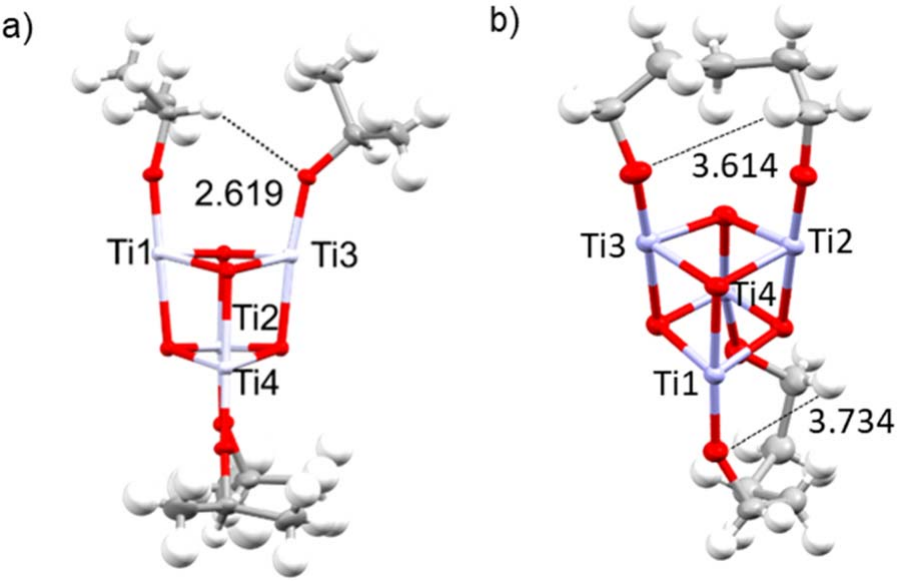
X-ray crystal structures of (a) 1^Ph^ and (b) 5^Ph^ (phosphinate ligands removed for clarity), showing the shortest O–C–H⋯O distances.

To further test these concepts, crystals of 5^Ph^ were irradiated with a 305 nm laser whilst mounted on an X-ray diffractometer at Diamond Light Source. Diffraction data was collected before and after 3 s of irradiation (laser power 55 mW). In contrast to the photoreactivity of 5^Ph^ in solution, no colour change was observed in the solid-state upon laser irradiation, and no changes to the structure were detected (Fig. S61). This implies that the photoredox reaction of 5^Ph^ is not productive in the crystal phase, contrasting with crystalline 1^Ph^, which turns blue and undergoes two-electron photoredox reactivity under a comparable dose of 305 nm laser light.^23^ This difference in reactivity may be attributed to the difficulty of 5^Ph^ to adopt a transition state to facilitate PCET within the confines of the crystal lattice, noting that the shortest O–C–H⋯O distances are >3.6 Å compared to ∼2.6 Å in 1^Ph^ (Fig. 6).

## Conclusion

Ti_4_-oxo clusters with bidentate bridging alkane diolate ligands ({O(CH_2_)_*n*_O}_2_, *n* = 2–5) were prepared and characterised. In comparison to 1^Ph^, which readily undergoes photoredox reactivity under UV light, the clusters with bridging alkane diolate ligands have differing photochemical reactivities. 5^Ph^ undergoes similar photoredox reactivity to 1^Ph^ at a similar rate, consistent with a two-electron intramolecular process on one face of the cluster, in which one end of an alkane diolate ligand is oxidised to an aldehyde and the other end becomes protonated, whilst the cluster becomes reduced by two electrons. The overall process selectively oxidises the alkane diolate to form a *n*-hydroxyaldehyde (which, in turn, cyclises to the lactol form). However, in contrast to 1^Ph^, photoreactivity of 5^Ph^ does not occur in the single-crystal phase, demonstrating that the PCET step is not sterically accessible, and suggesting that the solution phase mechanism progresses *via* a pendant alkoxide radical that requires sufficient space to sample a suitable transition state for intramolecular PCET. For clusters with smaller alkane diolate ligands, TEAS reveals a similar initial photoexcitation process to 5^Ph^ over a 1 ns time period, however, no productive photochemistry is observed, except for 3^Cy^, which is an outlier and produces only unidentified metastable redox products. These findings suggest that whilst initial photoexcitation occurs similarly across these clusters, in most cases the photoexcited state relaxes back to the initial compound, by radical recombination, and only in some cases, when the alkane diolate is large and flexible, the PCET step can occur to give stable photoredox products. Solvents also play important roles in photoreaction pathways, with coordinating solvents, such as pyridine, helping to block radical recombination pathways, and protic species improving photoyield by aiding PCET steps. These studies demonstrate the importance of atomic-level understanding when designing selective photoredox catalysts, where ligand coordination structure can influence excited-state lifetime and reaction products. The study indicates the potential of adjacent (O-bridged) Ti sites in clusters, MOFs, or other materials for coordinating to diolate species and emphasises the importance of PCET steps in photoredox mechanisms. The findings provide a structural design concept which could be incorporated into MOFs, or other framework structures to contribute to efforts to selectively oxidise low value polyols into higher value species using air, sunlight and Earth-abundant catalysts.

## Author contributions

SDP conceived and supervised the project. AC, ARV & SDP conducted synthesis, characterisation and photochemistry. SEB completed EPR studies. SDP and SEB conducted X-ray crystallography. AC and JMW conducted TEAS measurements. AC, supervised by FDP, conducted DFT calculations. SDP and AC wrote the manuscript with assistance from all other authors.

## Conflicts of interest

There are no conflicts to declare.

## Supplementary Material

SC-OLF-D5SC08522E-s001

SC-OLF-D5SC08522E-s002

## Data Availability

All raw data is deposited in the University of Warwick's database, WRAP at https://wrap.warwick.ac.uk/194815/. CCDC 2468340–2468346 contain the supplementary crystallographic data for this paper.^41*a*–*g*^ Supplementary information (SI): experimental and computational methods, supporting characterisation and spectra, X-ray crystallography details and computational data. See DOI: https://doi.org/10.1039/d5sc08522e.
