# Supplementary material for: Switching on photoreactivity in Ti4-oxo clusters by increasing the size of 1,n-alkane diolate bridging ligands
Source: Chem Sci. 2025 Nov 27;17(3):1870–9. doi: 10.1039/d5sc08522e (PMC12658559; doi:10.1039/d5sc08522e)
Supplement: SC-017-D5SC08522E-s001 [file SC-017-D5SC08522E-s001.pdf]

Supplementary Information (SI) for Chemical Science.
This journal is © The Royal Society of Chemistry 2025

Switching on photoreactivity in Ti_4-oxo clusters by increasing the size of 1,n-alkane diolate bridging ligands.

Ashwani Chikara,[1,2] Alexander R. Veale,[1] Stephen E. Brown,[1] Jack M. Woolley,[3,4] Frank De Proft,[2] Sebastian D. Pike*[1]

1. Department of Chemistry, University of Warwick, Coventry, UK.
2. Research Group of General Chemistry (ALGC), Vrije Universiteit Brussel (VUB), Brussels, Belgium.
3. Warwick Centre for Ultrafast Spectroscopy, Research Technology Platforms, University of Warwick, Coventry, UK.
4. Department of Physics, University of Warwick, Coventry, UK.

Supporting Information.

Contents **Page**

Contents	Page
Experimental Methods	1
Synthetic Procedures	3
Computational Methods	5
Supporting Figures	6
Supporting Note 1	48
Crystallography Details	48
Crystallography Table	50
Computational Data	51
References	59

Experimental

All manipulations were undertaken using a nitrogen filled glovebox or using a Schlenk line. Ph_2PO_2H and $Ti(O^iPr)_4$ were used directly from suppliers, Cy_2PO_2H was synthesised following a reported procedure.[1] THF and toluene were purchased as anhydrous grade. Pentane, dichloromethane and d^8-toluene were dried by refluxing over CaH_2 under nitrogen. These and all other dry solvents and reagents were degassed by bubbling with N_2 for 30 minutes or freeze pump thaw cycles and stored over 4 Å molecular sieves under nitrogen.

NMR spectra were recorded on Bruker 400 MHz instruments at 298 K and all chemical shifts reported in parts per million (ppm). Solution EPR experiments were performed in air-tight quartz EPR tubes with a Bruker EMX spectrometer operating at X-band (9.846 GHz) fitted with a variable temperature cryostat. EPR data was modelled using the EasySpin toolbox for MATLAB. Elemental Analysis were conducted by O. McCullough at London Metropolitan University. ESI-Mass spectra were collected using a Bruker Compact Q-TOF, using direct infusion in CH_2Cl_2 at 4µl/min. UV/vis spectra were recorded using a Shimadzu 2600i spectrometer. Absorption onsets were determined using Tauc's method, using n = 3.

Photoirradiation was undertaken using an Analytik Jena UVLM-26 EL series UV lamp (output 302 or 365 nm, 6 W). Experiments were conducted under a plastic cover to avoid exposure to UV. According to the manufacturers available data we anticipate the output for long wave UV irradiation to range from 325-400 nm, with a maximum output at 365 nm, and for the medium wave UV irradiation to range from 280-375 nm, with a maximum output at 302 nm. It is noteworthy that glass flasks begin to absorb photons <335 nm and will reduce the flux of any high energy photons from medium wave UV irradiation. For *in-situ* analysis of photoreactions by NMR spectroscopy the starting material (11 mg, ~8 µmol) and, in some cases, a sealed capillary containing PPh_3 and CH_2Cl_2 dissolved in $CDCl_3$, was loaded into a Young's tap NMR tube inside a glovebox. Solvent, typically d_8-toluene, 0.5 mL with 30 equiv. of pyridine, were added to the tube via syringe and micropipette respectively under a flow of N_2 using Schlenk line apparatus. The tube was sealed and then frozen with liquid nitrogen before exposure to vacuum, this cooling/vacuum procedure was repeated once more to ensure an air free environment before photoirradiation. If used, the capillary was tipped to the top of the tube and the tube laid in front of the UV lamp so that the solution (but not the capillary) was in the path of UV light, ~1 cm from the lamp.

Irradiation of single crystals of $\mathbf{5}^{\mathbf{Ph}}$ was undertaken at I19-EH2 at Diamond Light Source using a wavelength of 0.48590 Å and a four-circle Newport diffractometer equipped with a Dectris Eiger CdTe X 4M detector.[2] The single crystal was mounted on a glass loop and positioned on the diffractometer under a 100 K stream of N_2 gas and a diffraction dataset collected. The laser and X-ray spot were overlapped on the sample position. The crystal was then rotated under *in-situ* irradiation using the PORTO laser at 305 nm with a laser power of 55 mW (50 kHz repeat rate) for up to 3 s before re-collecting the diffraction pattern (note this is equivalent to 127 s under the irradiation conditions previously used for $\mathbf{1}^{\mathbf{Ph}}$)[2]. A further experiment for 180 s at 11 mW laser power (5 kHz repeat rate) also did not cause a colour change to the crystal. The PORTO is a femtosecond pulsed laser system comprising of a PHAROS Laser 20 W Ytterbium system from Light Conversion with an Orpheus HP optical parametric amplifier and 2nd and 4th harmonics options.

Transient electronic absorption spectroscopy (TEAS) measurements were performed at the Warwick centre for Ultrafast Spectroscopy (https://warwick.ac.uk/research/rtp/wcus/). The TEAS set-up used to collect the presented ultrafast spectroscopy data has been described previously;[3] hence only specific details about the reported experiments are given here: samples were held in an airtight 1 mm cuvette, placed at the focus of the probe beam and translated in the XY plane to ensure fresh sample was interrogated for each transient absorption spectrum. Sample degradation in the samples was monitored by comparing the ΔOD between each scan cycle; if drops in the signal were observed after a scan cycle, the samples were replenished before continuing. The excitation wavelength was chosen to be 350 nm and the 'pump' beam was focussed past the sample to ensure the probe interacted with a uniformly excited area. Pump-probe delays were generated through translating a hollow gold retroreflector on a motorized delay stage. For all measurements the relative polarisation between the pump and probe was held at magic angle (54.7°). Collected TAS were chirp corrected using the KOALA package.[4]

Synthetic Procedures.

All yields are recorded from the isolated microcrystalline powders following reaction in a Schlenk flask.

2^{Ph}. 100 mg (0.069 mmol) of **1^{Ph}** was placed in a Schlenk flask with 8 µL (0.14 mmol) 1,2-ethylene glycol and 10 mL toluene. A stirrer bar was added, and this sample was heated to 70 °C for a period of 6 days. The solution becomes cloudy once heated and the product forms as a fine precipitate. The soluble fraction was decanted and the product dried under vacuum. ~20 mg of product was isolated (24% yield).

$^{31}P\{^1H\}$ NMR spectroscopy ($CDCl_3$, 162 MHz): δ 34.3. ^1H NMR spectroscopy ($CDCl_3$, 400 MHz): δ 7.77 (16H, dd (J_{HH} = 7, J_{HP} 12 Hz), Ph), 7.39 (8H, m, Ph), 7.19 (16H, apparent td (J_{HH} = 7 & 7 Hz, J_{HP} = 3 Hz), Ph), 4.24 (8H, s, Ti-O-C**H$_2$**C**H$_2$**-O-Ti)

ESI-MS (CH_2Cl_2): No signal observed for **2^{Ph}** possibly due to difficulty forming protonated cation. Very weak signal for impurity '**2b^{Ph}**' observed, $[Ti_4O_4(O_2PPh_2)_4(O^iPr)_2(O(CH_2)_2O)]$; $[M-H]^+$ m/z = 1303.25, calc. 1303.07

Elemental Analysis (predicted): % C, 50.18 (48.94); % H, 3.16 (3.79).

2^{Cy}. 75 mg (0.053 mmol) of **1^{Cy}** was placed in a Schlenk flask with 6 µL (0.11 mmol) 1,2-ethylene glycol and 7.5 mL toluene. A stirrer bar was added, and this sample was heated to 70 °C for a period of 6 days. The reaction mixture remains dissolved throughout. The reaction solution was concentrated and placed in the freezer to crystallise. 25 mg (38% yield) of white powder isolated.

$^{31}P\{^1H\}$ NMR spectroscopy (d_8-toluene, 162 MHz): δ 56.1. ^1H NMR spectroscopy (d_8-toluene, 400 MHz): δ 4.55 (8H, t (J_{HH} = 5 Hz), Ti-O-C**H$_2$**C**H$_2$**-O-Ti), 2.18 (16H, m, Cy), 1.84 (24H, m, Cy), 1.67 (8H, m Cy), 1.60 (16H, m, Cy), 1.26 (24H, m, Cy).

ESI-MS (CH_2Cl_2): $[M-H]^+$ m/z = 1293.37, calc. 1293.37

3^{Ph}. 100 mg (0.069 mmol) of **1^{Ph}** was placed in a Schlenk flask with 10 µL (0.138 mmol) 1,3-propanediol and 10.5 mL toluene. A stirrer bar was added, and this sample was heated to 70 °C for a period of 4 days. The solution becomes cloudy after 4 hours and the product forms as a precipitate. 66 mg of precipitate was isolated (75% yield).

$^{31}P\{^1H\}$ NMR spectroscopy ($CDCl_3$, 162 MHz): δ 34.1. ^1H NMR spectroscopy ($CDCl_3$, 400 MHz): δ 7.83 (16H, dd, Ph), 7.39 (8H, t, Ph), 7.19 (16H, m, Ph), 4.28 (8H, t (J_{HH} = 5 Hz), Ti-O-C**H$_2$**CH$_2$C**H$_2$**-O-Ti), 1.61 (4H, quintet (J_{HH} = 5 Hz), Ti-O-CH$_2$C**H$_2$**CH$_2$-O-Ti).

ESI-MS (CH_2Cl_2): $[M-H]^+$ m/z = 1273.0, calc. 1273.0.

Elemental Analysis (predicted): % C, 49.10 (50.98); % H, 3.81 (4.12). N.B. adding 0.8 equivalents of CH_2Cl_2 to the formula gives a good representation of the collected data (%C, 49.11; %H, 4.03).

3^{Cy}. 430 mg (0.305 mmol) of **1^{Cy}** was placed in a Schlenk flask with 44 µL (0.610 mmol) 1,3-propanediol and 12 mL toluene. A stirrer bar was added, and this sample was heated to 70 °C for a period of 6 days. The reaction mixture remains dissolved throughout. All volatiles were then removed under vacuum. The product is then washed with pentane to remove any impurities or starting material. 200 mg white powder isolated (50% yield).

$^{31}P\{^1H\}$ NMR spectroscopy (d_8-toluene, 162 MHz): δ 55.9. ^1H NMR spectroscopy (d_8-toluene, 400 MHz): δ 4.80 (8H, t (J_{HH} = 5 Hz), Ti-O-C**H₂**CH₂C**H₂**-O-Ti), 2.19 (16H, m, Cy), 1.83 (28H, m, Cy + Ti-O-CH₂C**H₂**CH₂-O-Ti), 1.68 (8H, m, Cy), 1.61 (16H, m, Cy), 1.27 (24H, m, Cy).

ESI-MS (CH_2Cl_2): $[M-H]^+$ m/z = 1321.4, calc. 1321.4.

Elemental Analysis (predicted): % C, 48.20 (49.10); % H, 7.62 (7.63).

4^{Ph}. 100 mg (0.069 mmol) of **1^{Ph}** was placed in a Schlenk flask with 12.3 µL (0.138 mmol) 1,4-butanediol and 10 mL toluene. A stirrer bar was added, and this sample was heated to 70 °C for a period of 6 days. The reaction mixture remains dissolved throughout. The solution was concentrated under vacuum and then cooled to –20° to crystallise. 40 mg of microcrystalline product was isolated (45% yield).

$^{31}P\{^1H\}$ NMR spectroscopy ($CDCl_3$, 162 MHz): δ 34.0. ^1H NMR spectroscopy ($CDCl_3$, 400 MHz): δ 7.85 (16H, dd, Ph), 7.40 (8H, t, Ph), 7.19 (16H, m, Ph), 4.32 (8H, br, Ti-O-C**H₂**CH₂CH₂C**H₂**-O-Ti), 1.42 (8H, br, Ti-O-CH₂C**H₂**C**H₂**CH₂-O-Ti).

ESI-MS (CH_2Cl_2): $[M-H]^+$ m/z = 1301.05, calc. 1301.05

Elemental Analysis (predicted): % C, 51.82 (51.72); % H, 4.01 (4.34).

4^{Cy}. 140 mg (0.099 mmol) of **1^{Cy}** was placed in a Schlenk flask with 18 µL (0.20 mmol) 1,4-butanediol and 6 mL toluene. A stirrer bar was added, and this sample was heated to 70 °C for a period of 10 days. The reaction mixture remains dissolved throughout. All volatiles were then removed under vacuum. The product was redissolved in hot heptane and then cooled to –20°C overnight. The soluble fraction was decanted and the precipitate was dried under vacuum. 25 mg (20% yield) of white powder was isolated. Crystals can be prepared by suspending in heptane and adding a few drops of dichloromethane, then allowing the solution to slowly evaporate.

$^{31}P\{^1H\}$ NMR spectroscopy (d_8-toluene, 162 MHz): δ 55.2. ^1H NMR spectroscopy (d_8-toluene, 400 MHz): δ 4.72 (8H, br, Ti-O-C**H₂**CH₂CH₂C**H₂**-O-Ti), 2.18 (16H, m, Cy), 1.87 (8H, br, Ti-O-CH₂C**H₂**C**H₂**CH₂-O-Ti), 1.84 (24H, m, Cy +), 1.68 (8H, m, Cy), 1.61 (16H, m, Cy), 1.27 (24H, m, Cy).

Elemental Analysis (predicted): % C, 49.40 (49.90); % H, 7.80 (7.80).

ESI-MS (CH_2Cl_2): $[M-H]^+$ m/z = 1349.44, calc. 1349.43

5^{Ph}. 100 mg (0.069 mmol) of **1^{Ph}** was placed in a Schlenk flask with 14.5 µL (0.138 mmol) 1,5-pentanediol and 10 mL toluene. A stirrer bar was added, and this sample was heated to 70 °C for a period of 6 days. The reaction mixture remains dissolved throughout. The solution was concentrated under vacuum and then cooled to –20° to crystallise. ~30mg mg of precipitate was isolated (31% yield).

$^{31}P\{^1H\}$ NMR spectroscopy (d^8-toluene, 162 MHz): δ 32.8. ^1H NMR spectroscopy (d^8-toluene, 400 MHz): δ 7.98 (16H, dd, Ph), 7.08 (8H, m, Ph), 6.97 (16H, m, Ph), 4.44 (8H, t (J_{HH} = 5 Hz), Ti-O-C**H₂**CH₂CH₂CH₂C**H₂**-O-Ti), 1.49 (4H, m, Ti-O-CH₂CH₂C**H₂**CH₂CH₂-O-Ti), 1.35 (8H, m, Ti-O-CH₂C**H₂**CH₂C**H₂**CH₂-O-Ti).

ESI-MS (CH_2Cl_2): $[M-H]^+$ m/z = 1329.08, calc. 1329.08

Elemental Analysis (predicted for **5^{Ph}**·(C_7H_8)): % C, 53.57 (53.75); % H, 4.49 (4.72). Note partial loss of co-crystallised toluene during drying process.

3b^{Cy}. This can be prepared as a mixture with **3^{Cy}** by adding an excess (30 equivalents) of ^{i}PrOH to a toluene solution of **3^{Cy}** and allowing to stir for three days at room temperature.

$^{31}P\{^{1}H\}$ NMR spectroscopy (d_8-toluene, 400 MHz): δ 55.4. ^{1}H NMR spectroscopy (d_8-toluene, 400 MHz, Cy peaks overlapping with **3^{Cy}**): δ 5.17 (2H, sept (J_{HH} = 6 Hz), Ti-O^{i}Pr), 4.80 (4H, t (JHH = 5.5 Hz), Ti-O-C**H$_2$**CH$_2$C**H$_2$**-O-Ti), 2.17 (16H, m, Cy), 1.81 (24H, m, Cy + Ti-O-CH$_2$C**H$_2$**CH$_2$-O-Ti), 1.66 (8H, m Cy), 1.60 (16H, m, Cy), 1.45 (12H, d (J_{HH} = 6 Hz), Ti-O^{i}Pr), 1.26 (24H, m, Cy).

ESI-MS (CH_2Cl_2): $[M-H]^+$ m/z = 1365.5, calc. 1365.5.

Computational Methods

All DFT and TDDFT computations were performed using Gaussian 16 (Revision C.01).[5] All geometry optimisations and frequency analysis were carried out in the gas phase at the PBE/def2-SVP level of theory with Grimme's D3(BJ) dispersion correction.[6-9] Starting coordinates were taken from the corresponding X-ray CIF files. All stationary points were verified as minima (no imaginary frequencies).

Single-point energies were then computed with the SMD chloroform continuum model, retaining PBE and a mixed basis set (SDD + ECP on Ti; def2-TZVP on O, P; def2-SVP on C, H).[6, 7, 10, 11] Electronic structures were interpreted through canonical molecular orbitals and Mulliken population analysis as implemented in Gaussian 16.

Excited-state properties were evaluated via time-dependent density-functional theory within the Tamm-Dancoff approximation (TD-DFT/TDA, 160 singlet states) at the PBE0 level, using the same solvation model, dispersion correction, and basis-set scheme.[12-15]

To interpret the TDDFT excitation results more clearly, we employed Natural Transition Orbitals (NTOs).[16] These orbitals offer a simplified representation of electronic transitions, as most excitations can be described by a small number of dominant NTO pairs. Unlike conventional molecular orbital-based descriptions, NTOs provide a more concise picture by highlighting the key donor and acceptor orbitals involved in the excitation, weighted by their contribution to the transition.

Electron difference-density plots (excited - ground state) were produced with Multiwfn v3.8 and visualised in VMD 1.9.4, contoured at ±0.003 a.u.; red and blue surfaces denote electron depletion and accumulation, respectively.[17, 18]

Supporting Figures.

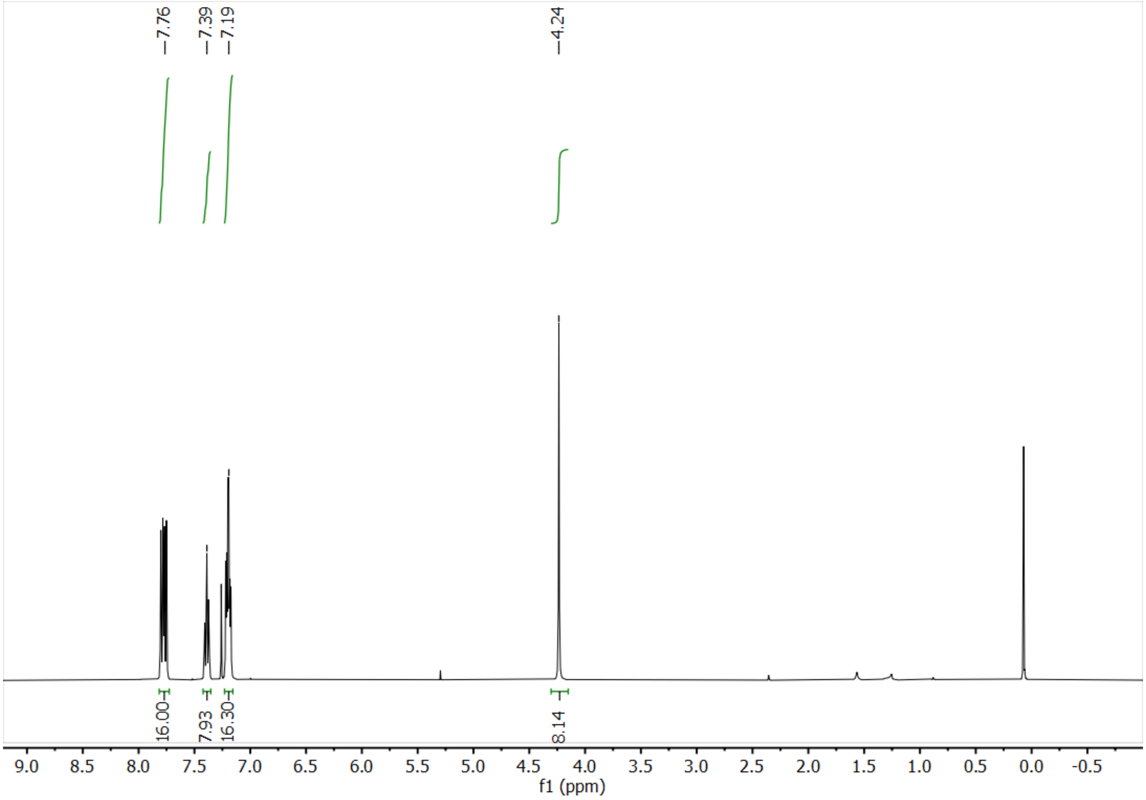

Figure S1. ^{1}H NMR spectrum of **2^{Ph}** in $CDCl_3$.

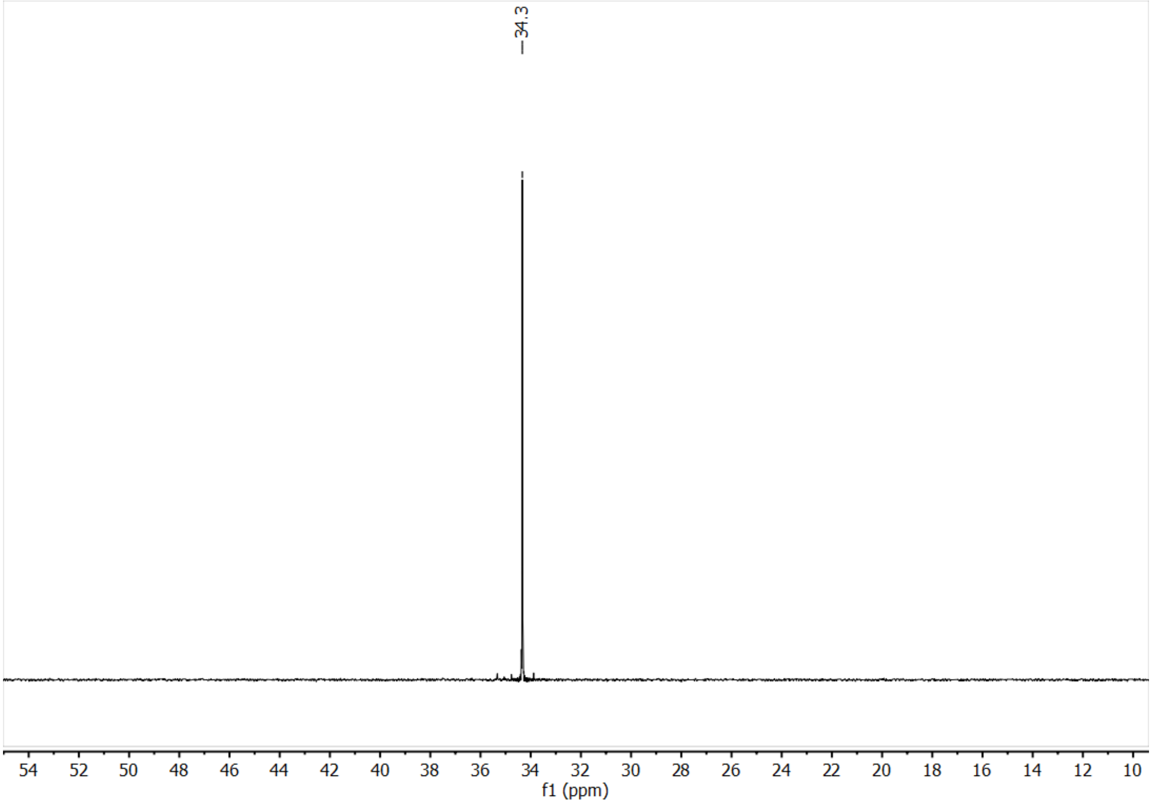

Figure S2. $^{31}P\{^{1}H\}$ NMR spectrum of **2^{Ph}** in $CDCl_3$.

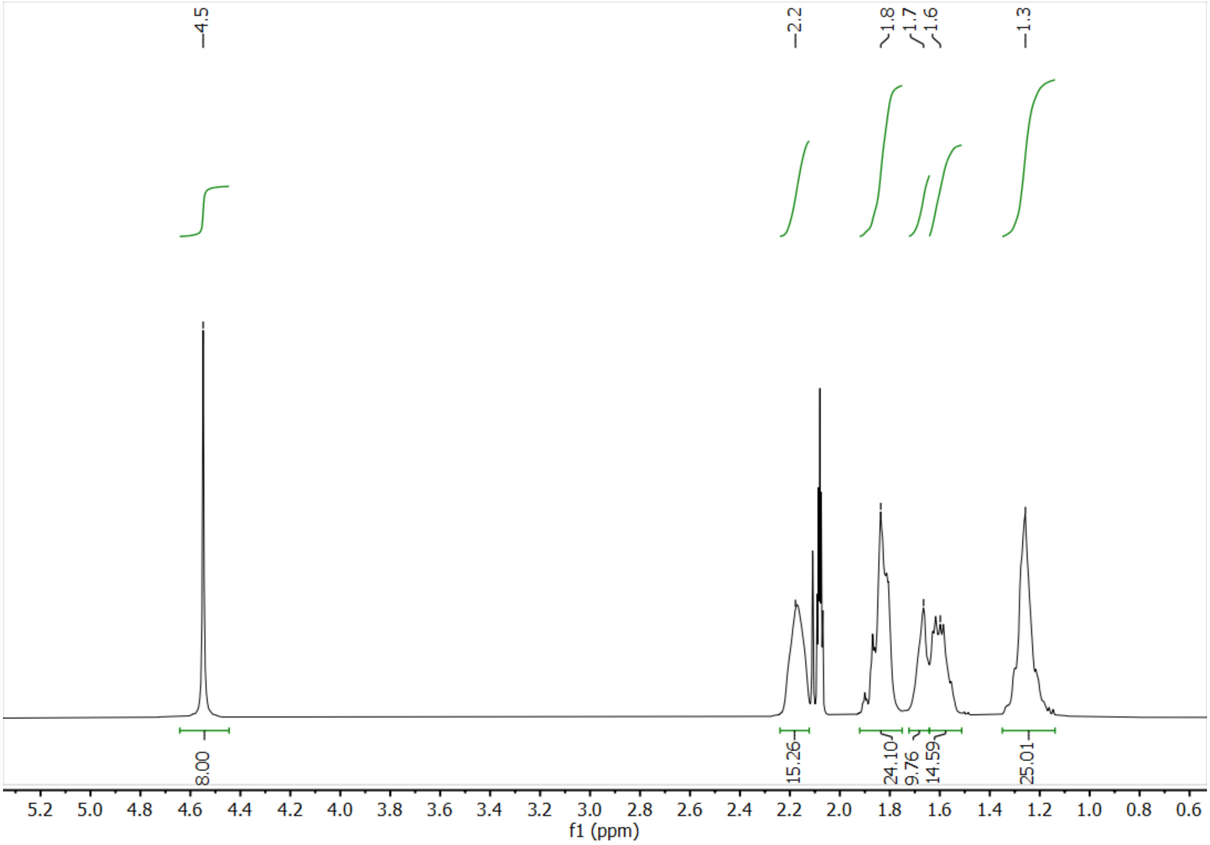

Figure S3. ^{1}H NMR spectrum of $\mathbf{2}^{Cy}$ in d^{8}-toluene.

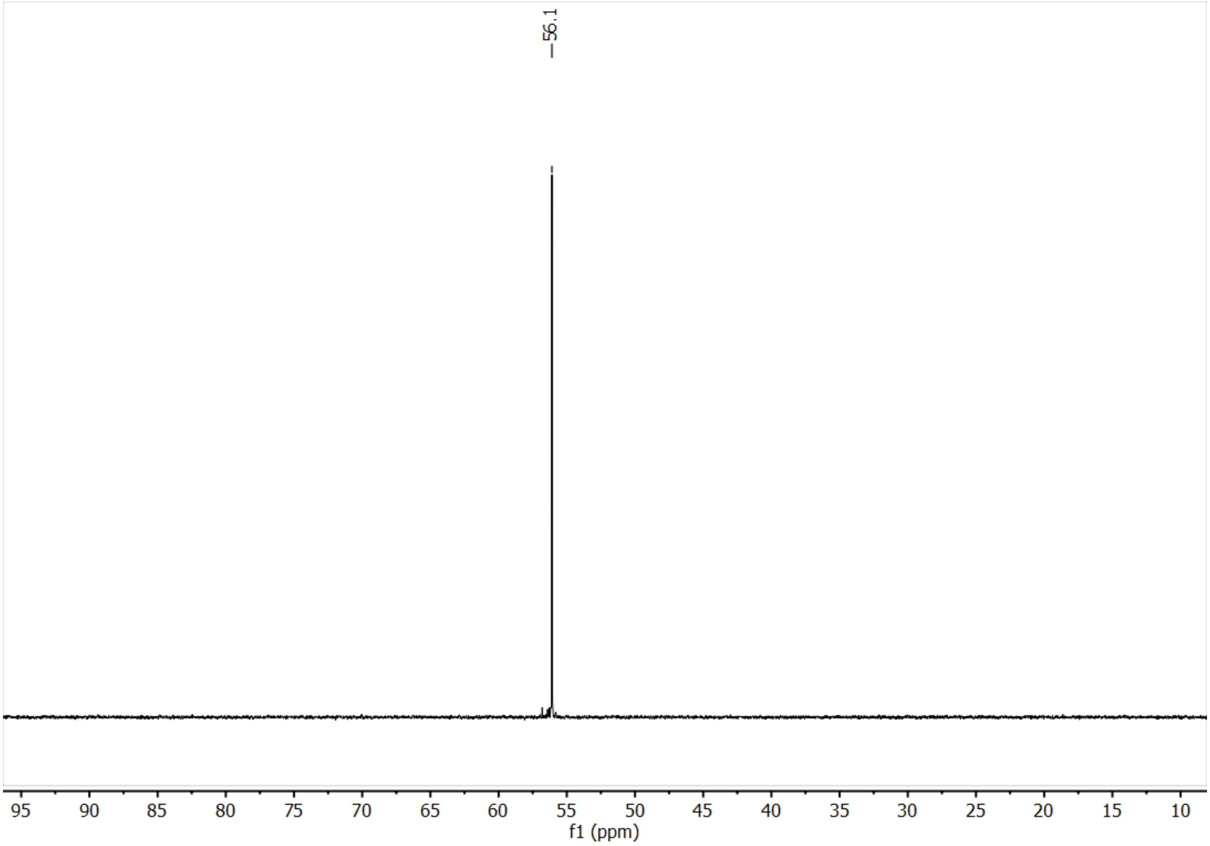

Figure S4. $^{31}P\{^{1}H\}$ NMR spectrum of $\mathbf{2}^{Cy}$ in d^{8}-toluene.

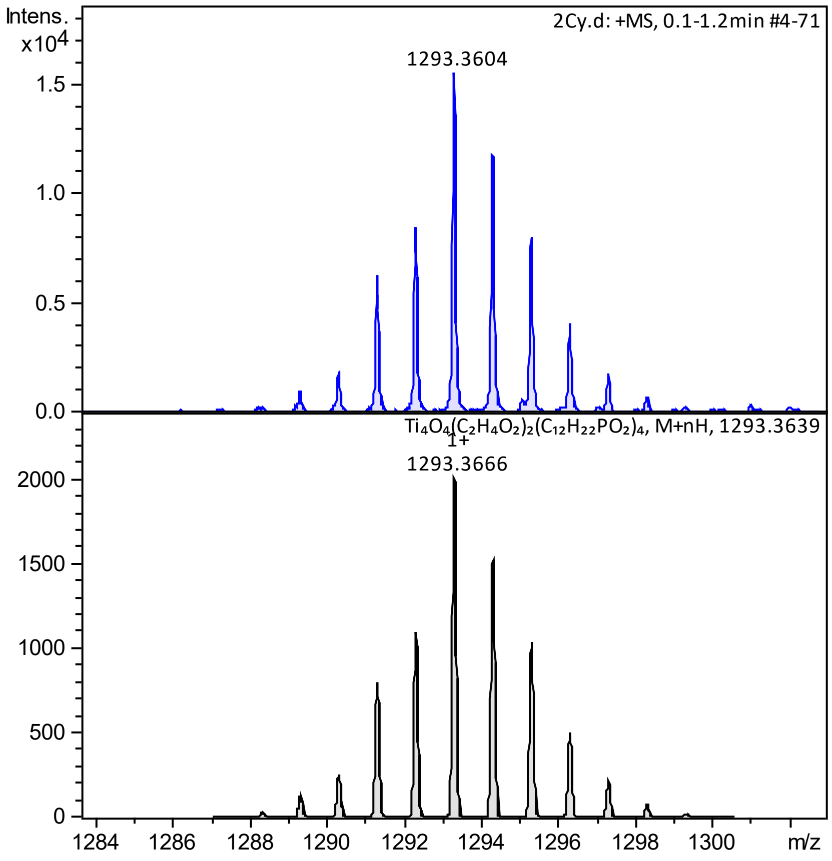

Figure S5. ESI mass spectrometry data for $\mathbf{2}^{\mathbf{Cy}}$+H^+ in CH_2Cl_2

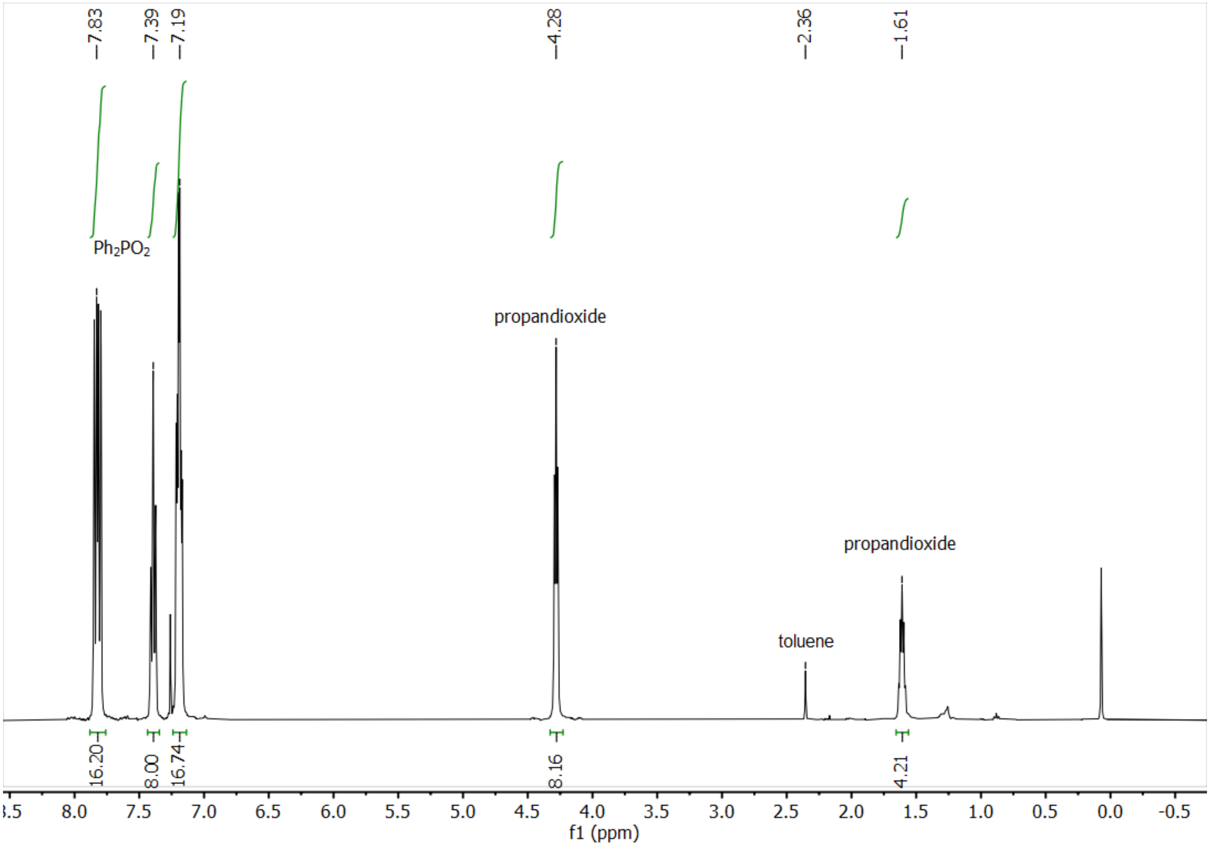

Figure S6. ^{1}H NMR spectrum of $\mathbf{3}^{\mathbf{Ph}}$ in $CDCl_3$

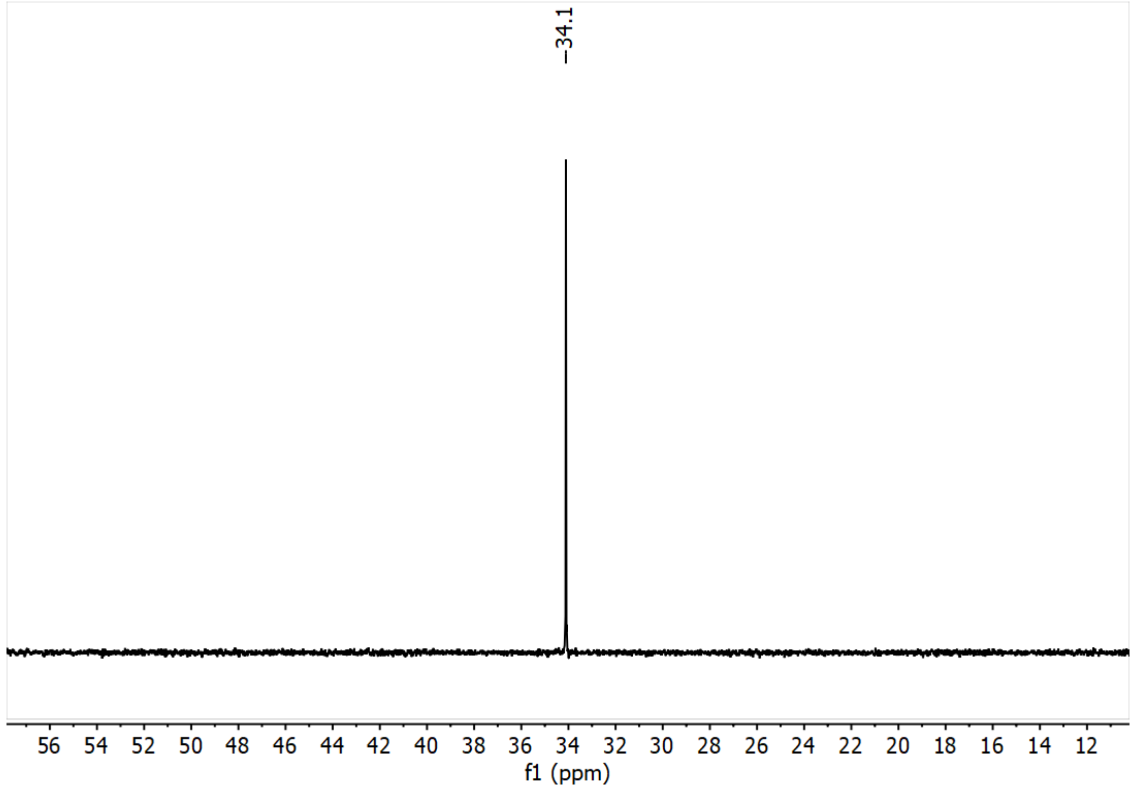

Figure S7. $^{31}P\{^{1}H\}$ NMR spectrum of **3**$^{\mathbf{Ph}}$ in $CDCl_3$.

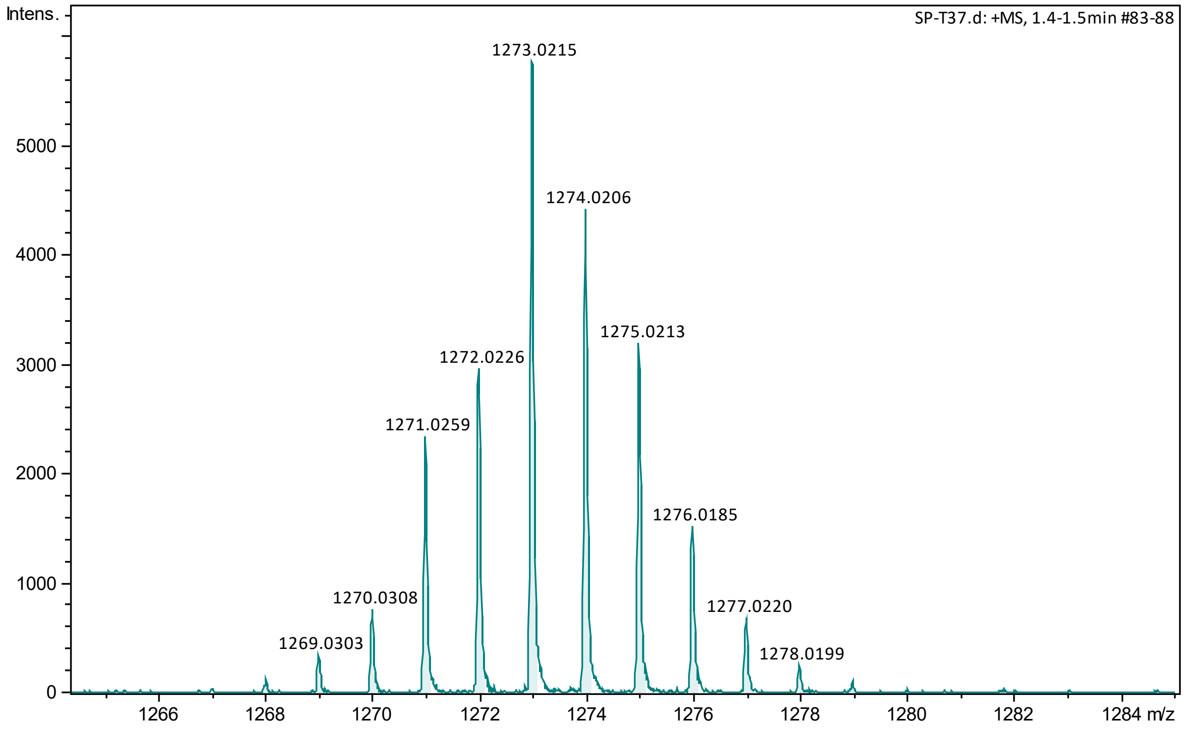

Figure S8. ESI mass spectrometry data for **3**$^{\mathbf{Ph}}$+H^+ in CH_2Cl_2

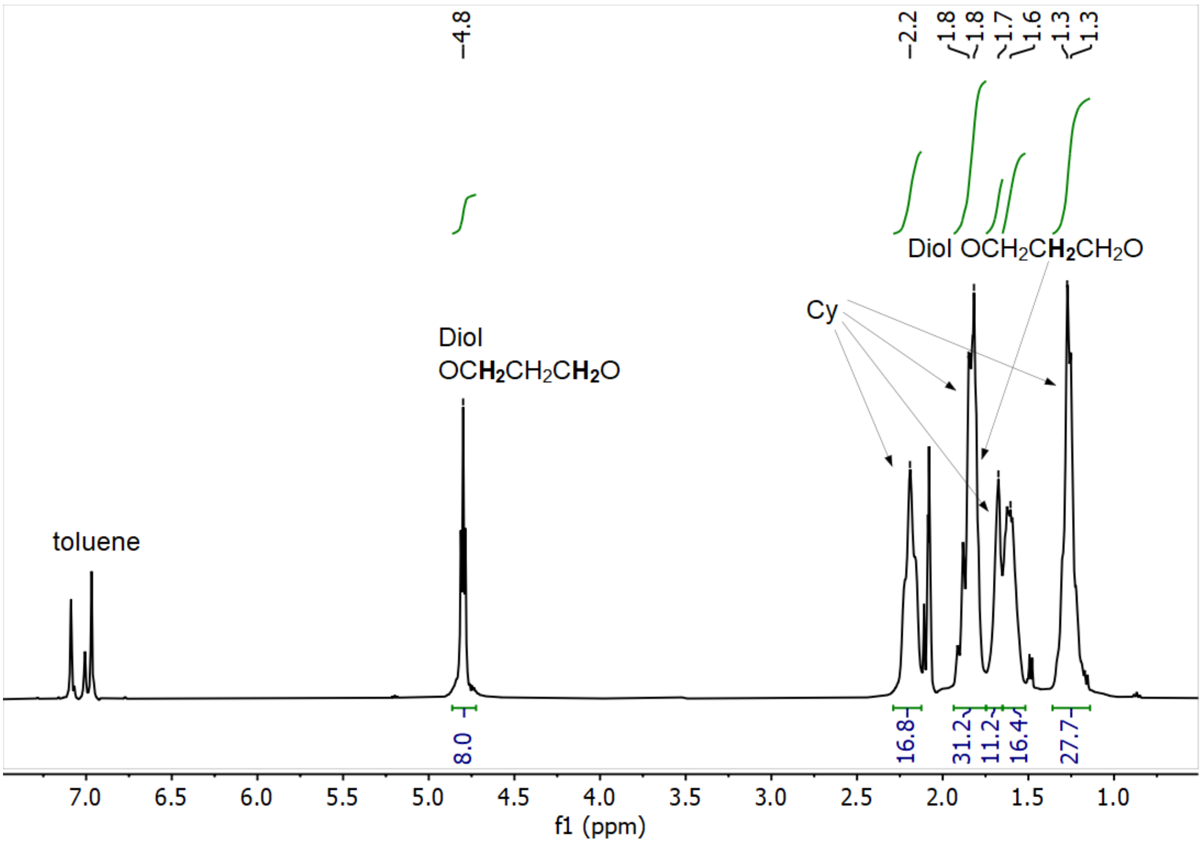

Figure S9. ^{1}H NMR spectrum of $\mathbf{3^{Cy}}$ in d^{8}-toluene

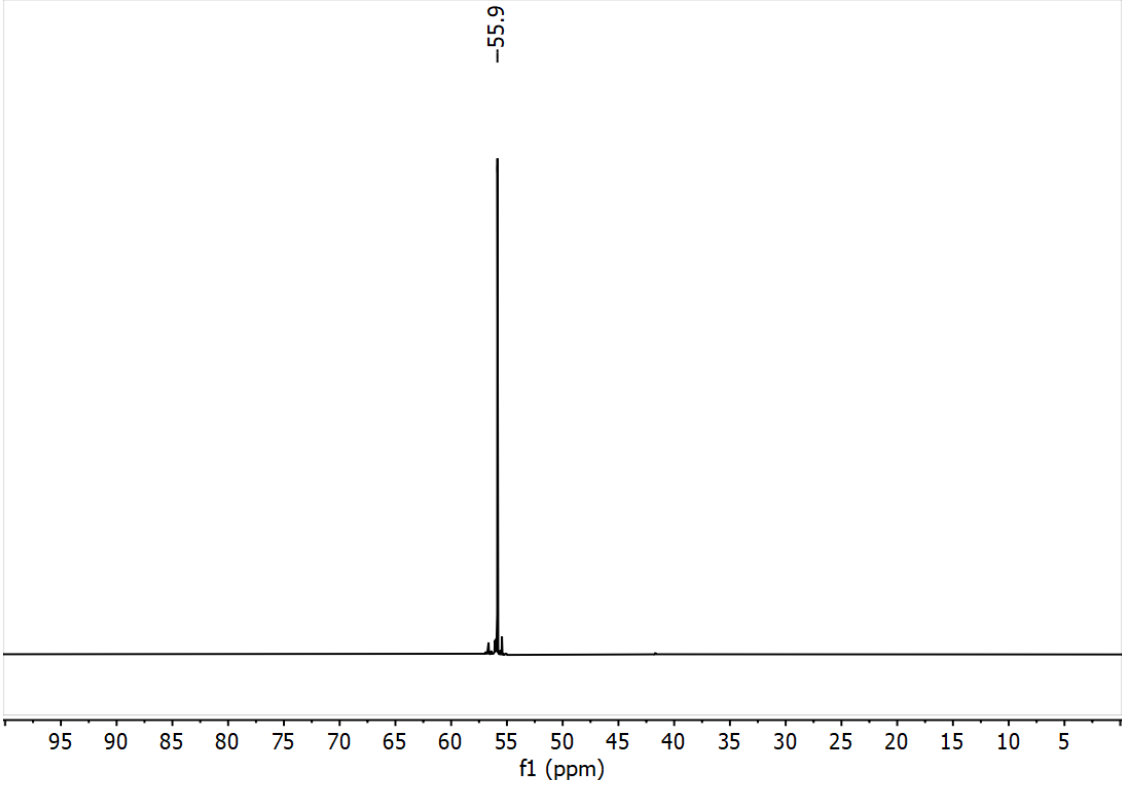

Figure S10. $^{31}P\{^{1}H\}$ NMR spectrum of $\mathbf{3^{Cy}}$ in d^{8}-toluene

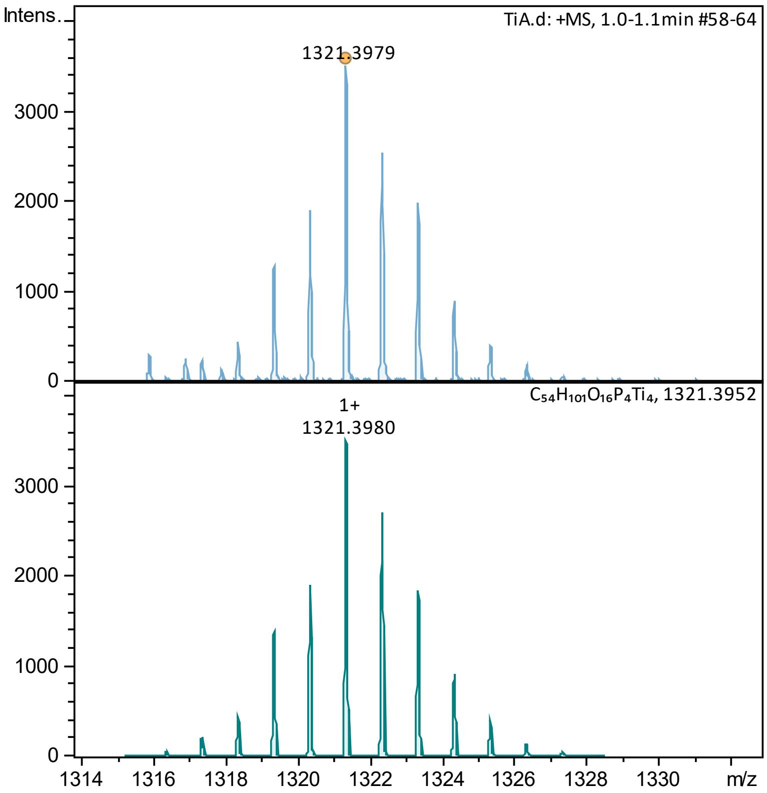

Figure S11. ESI mass spectrometry data for **3**^{Cy}+H$^+$ in CH_2Cl_2

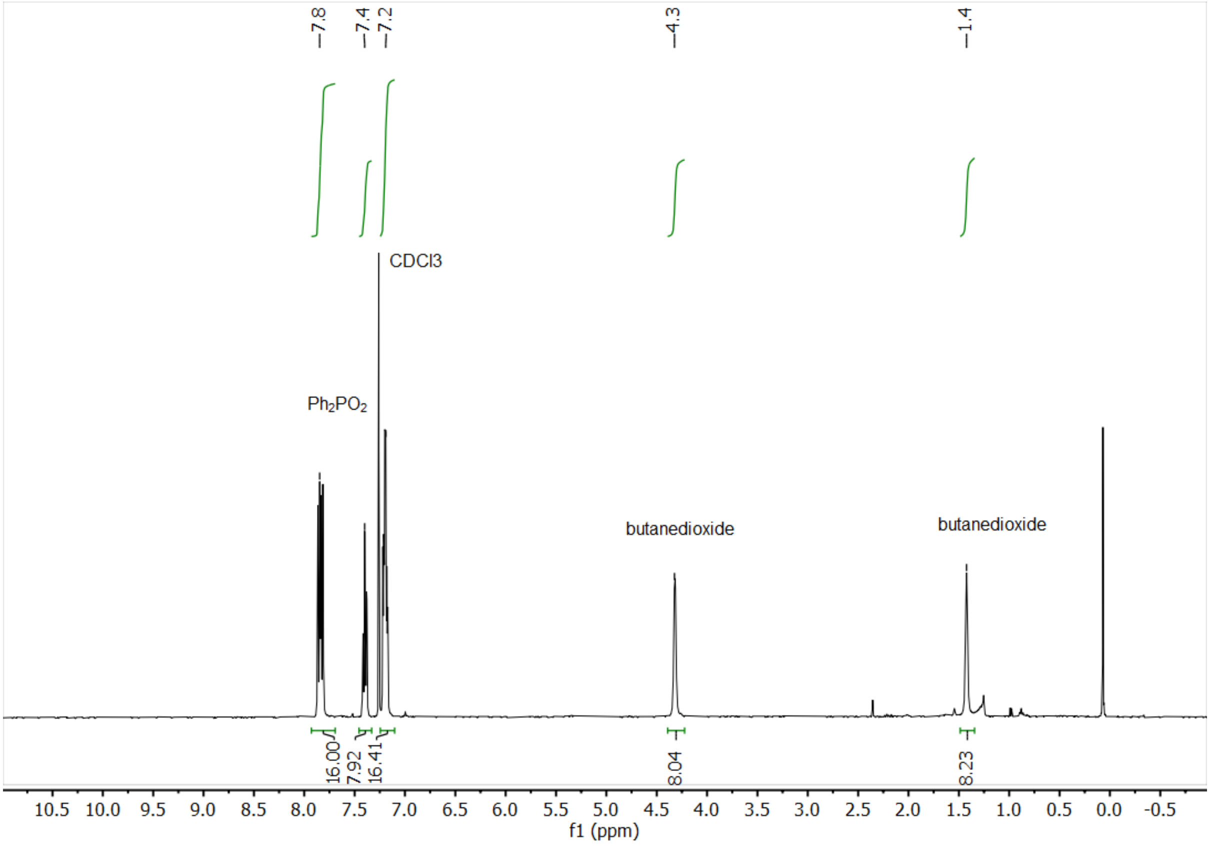

Figure S12. ^{1}H NMR spectrum of **4**^{Ph} in $CDCl_3$

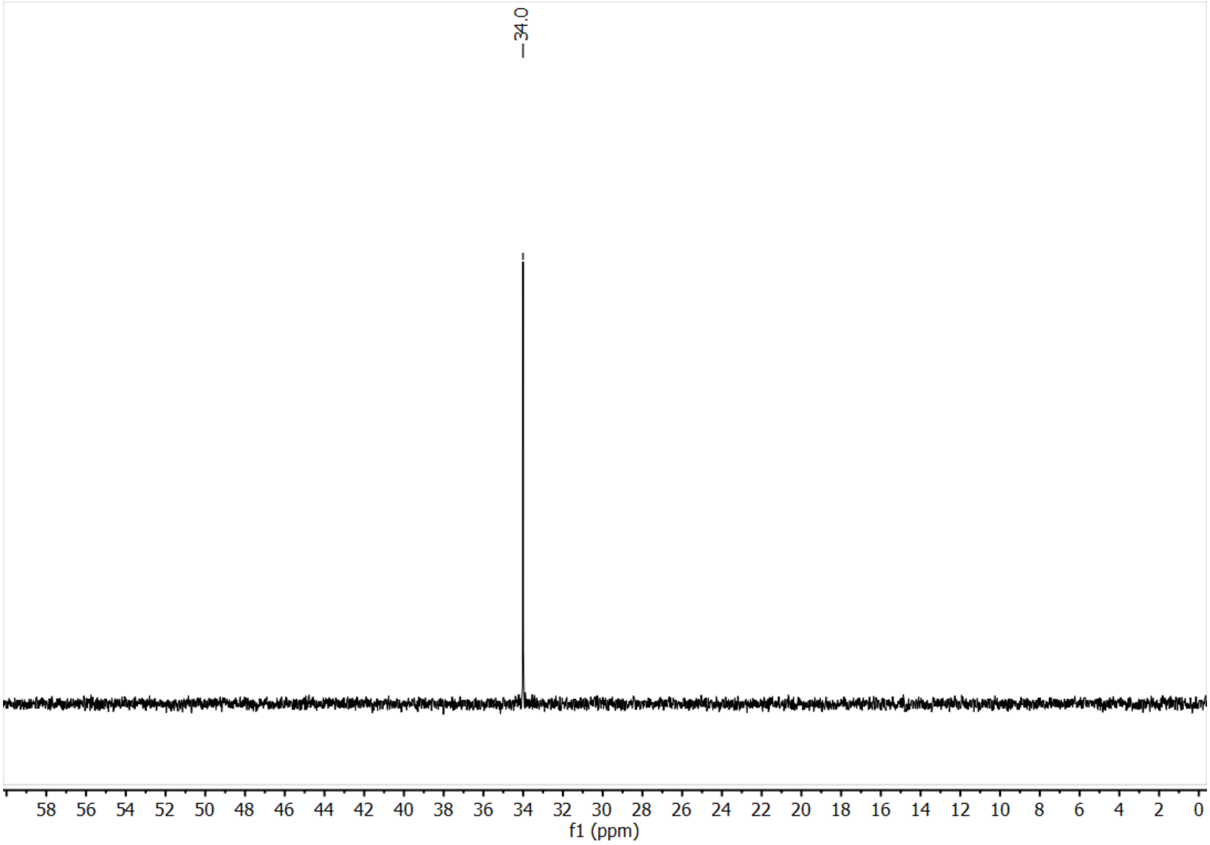

Figure S13. $^{31}P\{^{1}H\}$ NMR spectrum of $\mathbf{4^{Ph}}$ in $CDCl_3$.

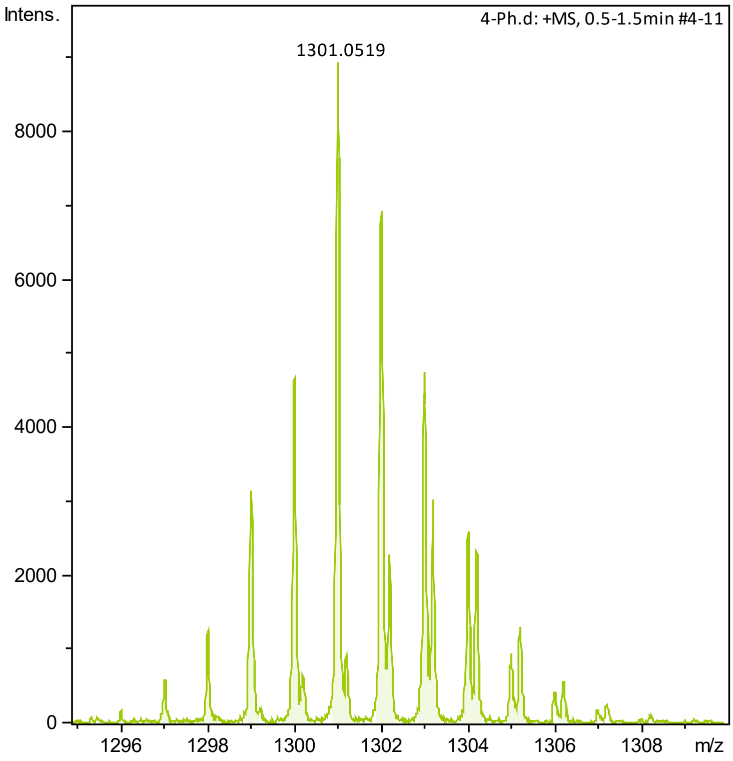

Figure S14. ESI mass spectrometry data for $\mathbf{4^{Ph}}+H^{+}$ in CH_2Cl_2

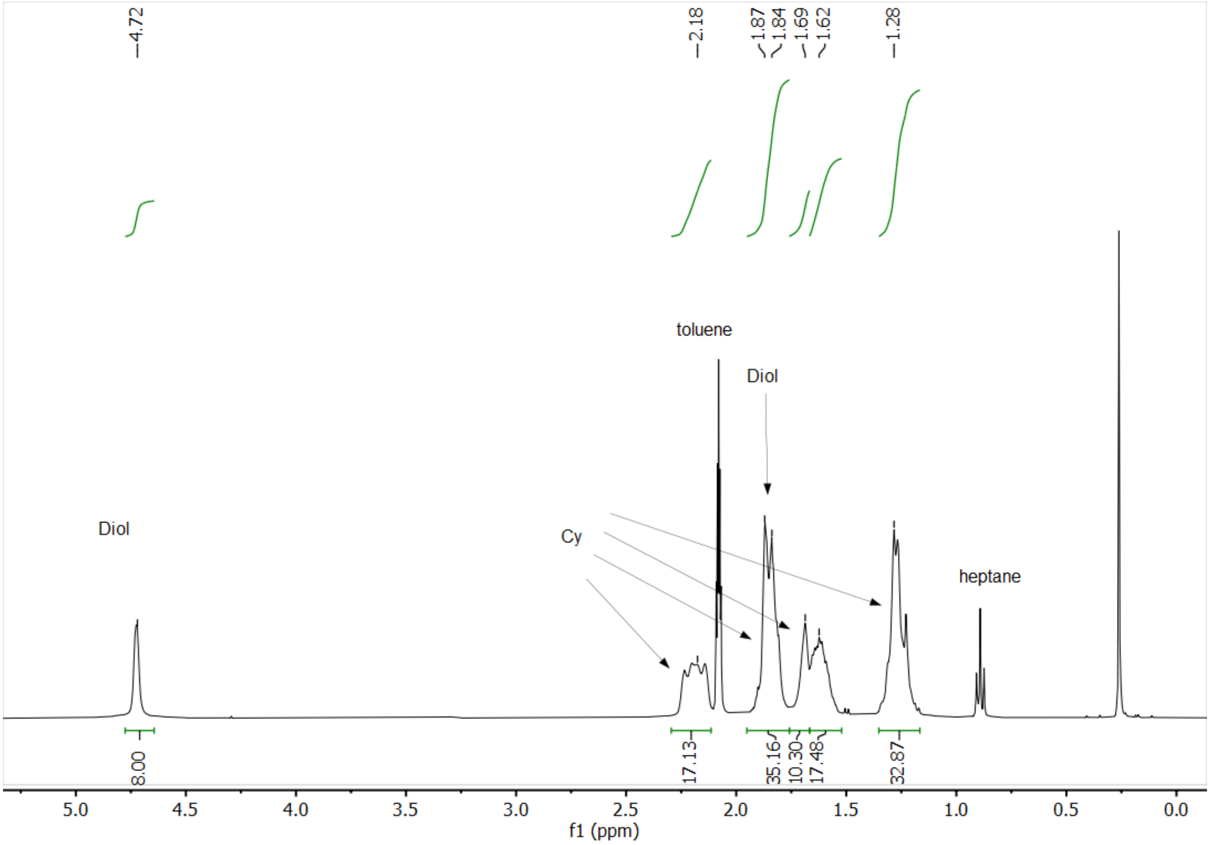

Figure S15. ^{1}H NMR spectrum of **4**^{Cy} in d^{8}-toluene.

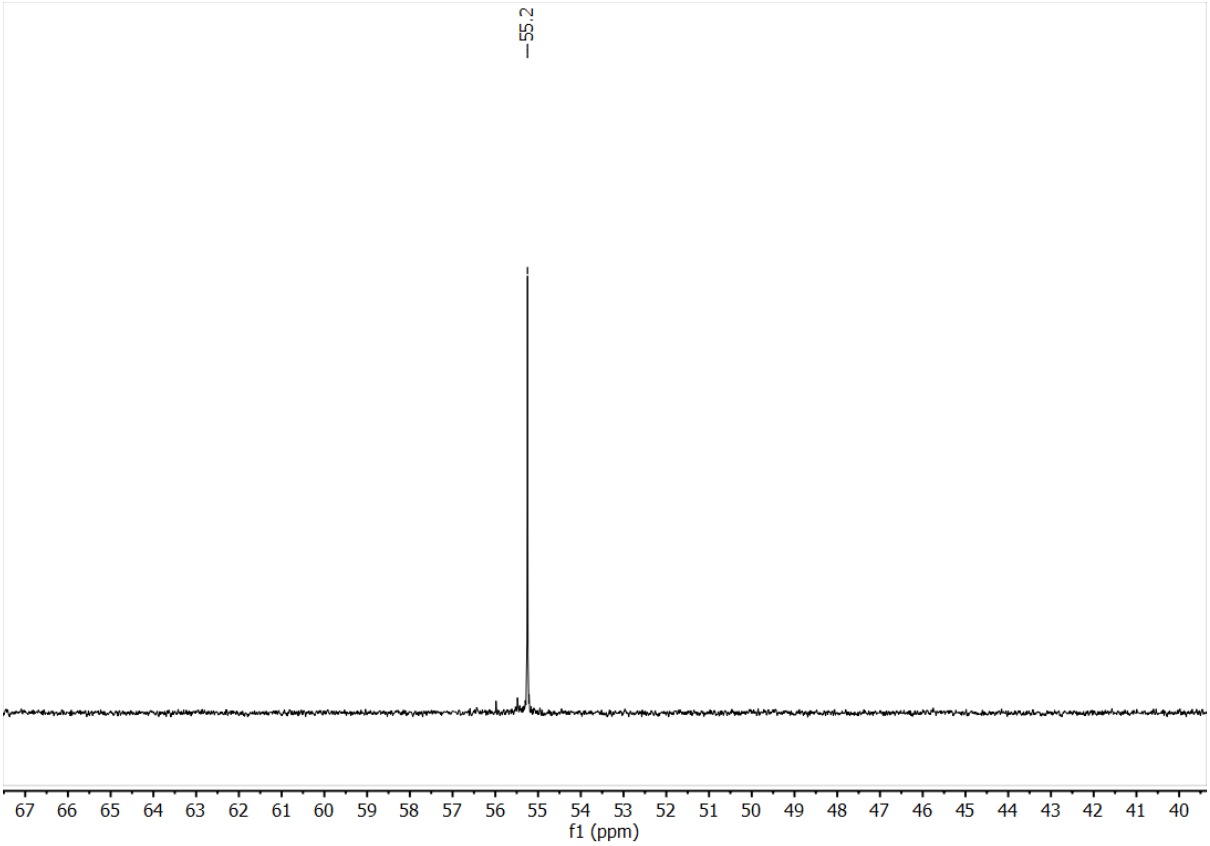

Figure S16. $^{31}P\{^{1}H\}$ NMR spectrum of **4**^{Cy} in d^{8}-toluene.

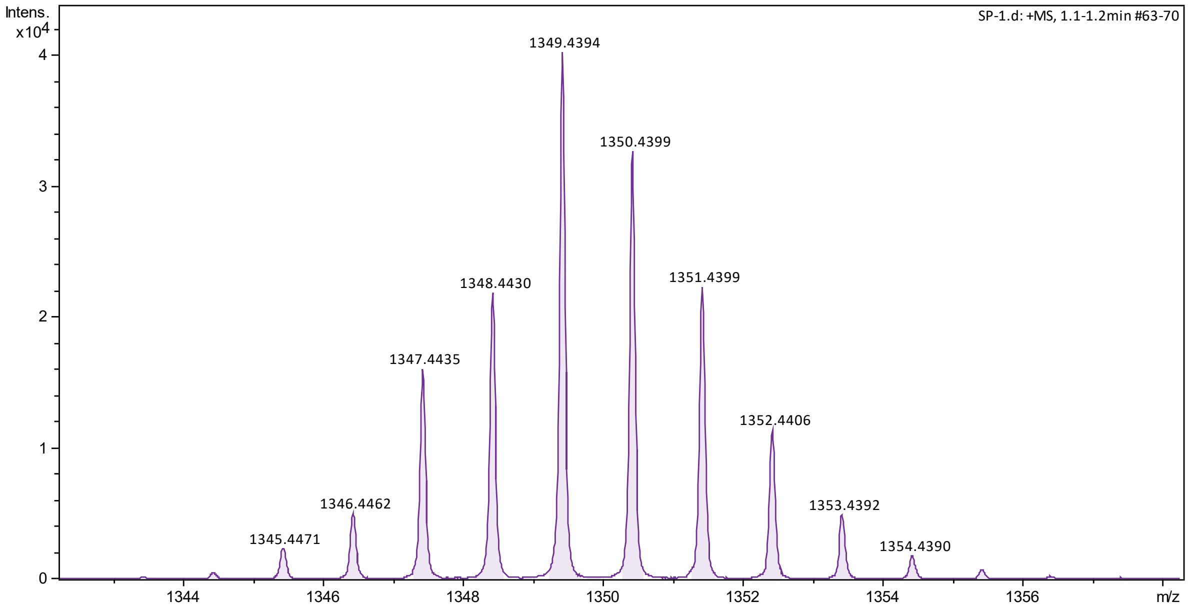

Figure S17. ESI mass spectrometry data for $\mathbf{4}^{\mathbf{Cy}}$+H^+ in CH_2Cl_2

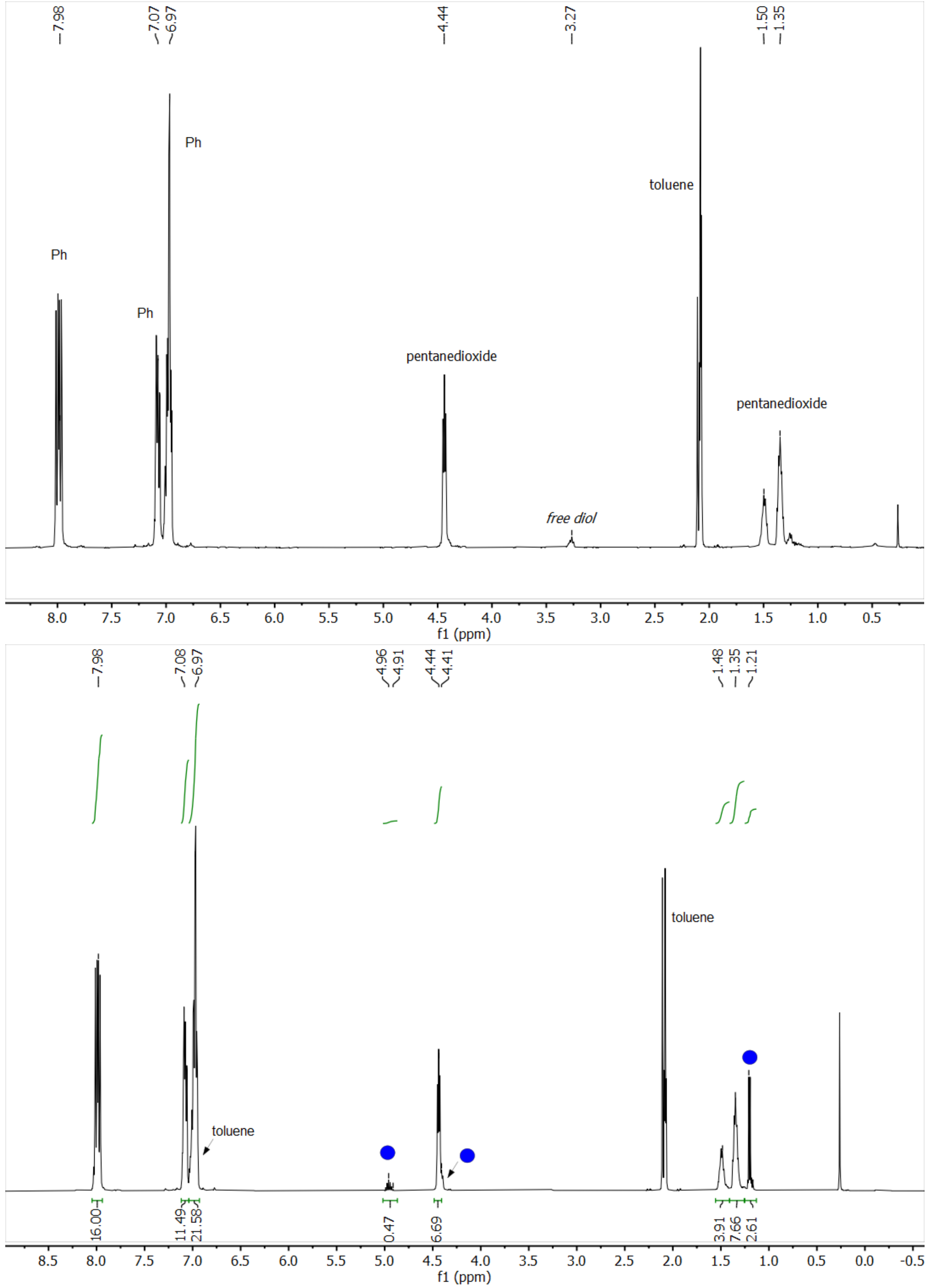

Figure S18. ^{1}H NMR spectra of two batches of **5^{Ph}** in d^8-toluene. Signals marked with blue spot are $[Ti_4O_4(O(CH_2)_5O)(O^iPr)_2(O_2PPh_2)_4]$ (**$5b^{Ph}$**) which forms as an intermediate during alkoxide exchange.

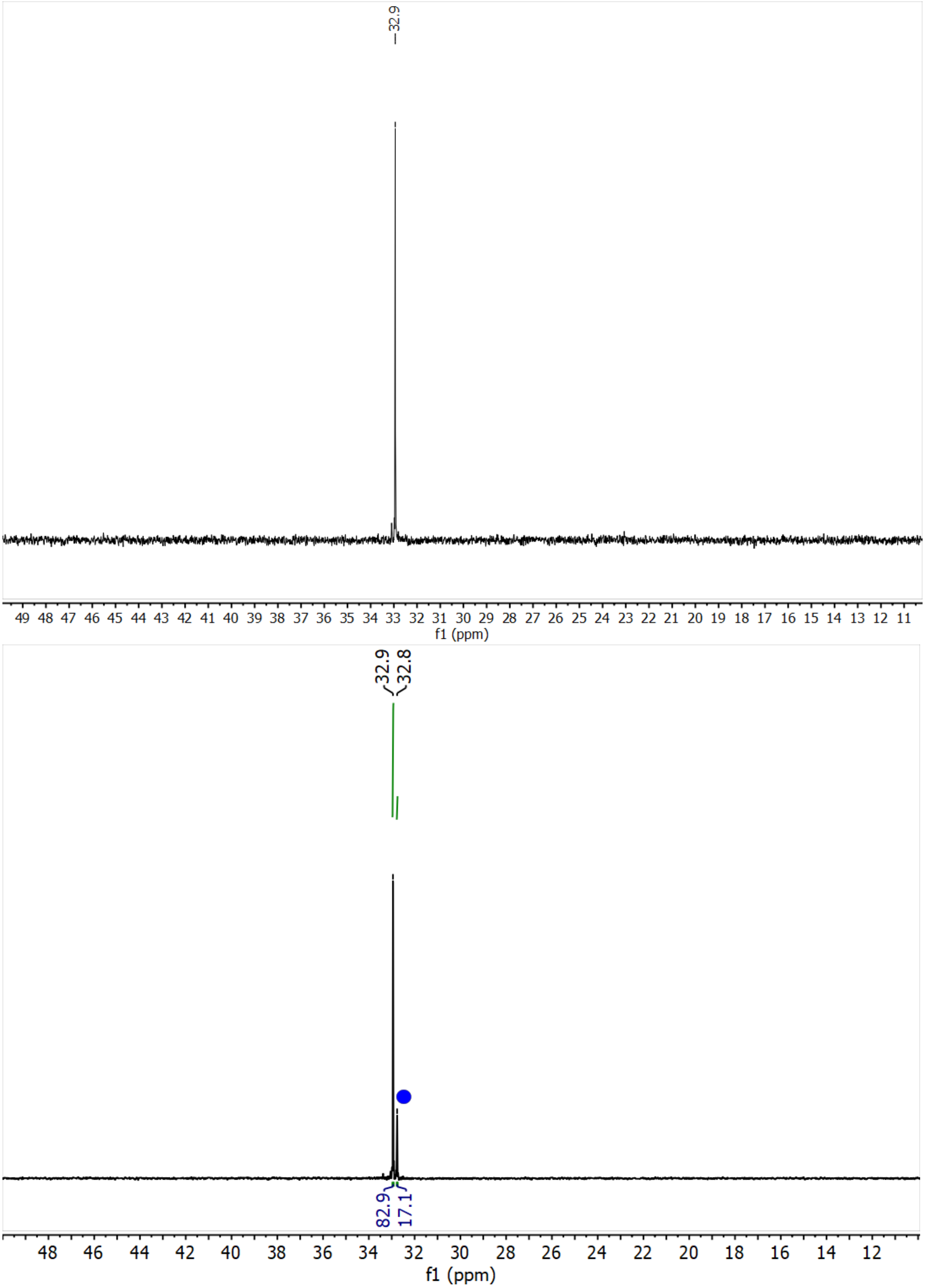

Figure S19. $^{31}P\{^{1}H\}$ NMR spectra of two batches of **5^{Ph}** in d^8-toluene. Signal marked with blue spot is $[Ti_4O_4(O(CH_2)_5O)(O^iPr)_2(O_2PPh_2)_4]$ (**$5b^{Ph}$**) which forms as an intermediate during alkoxide exchange.

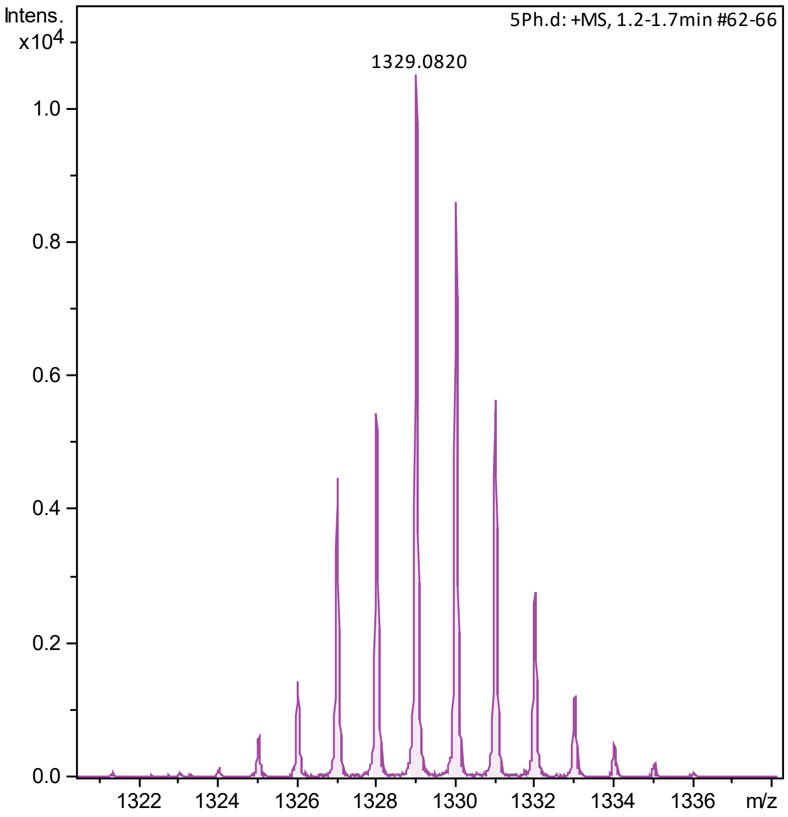

Figure S20. ESI mass spectrometry data for **5**^{Ph}+H$^+$ in CH_2Cl_2

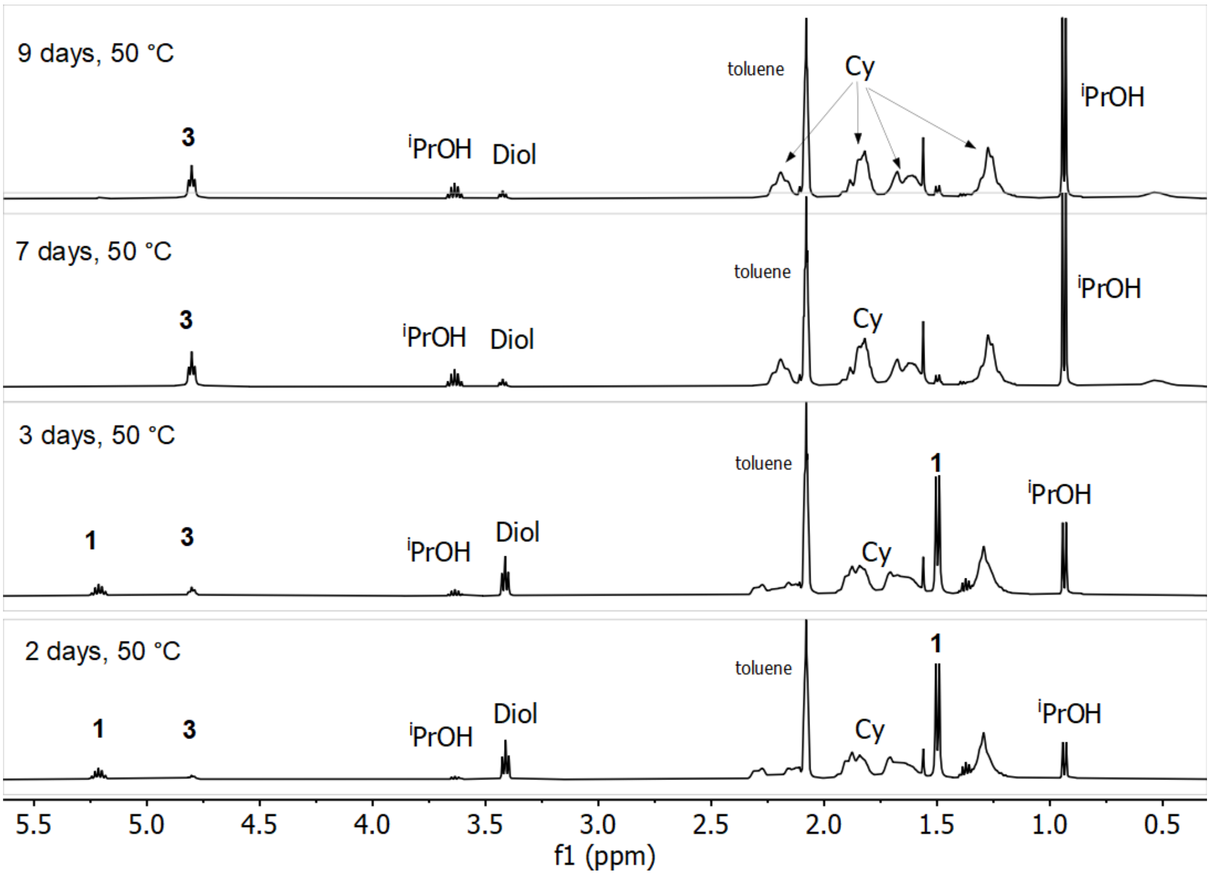

Figure S21. In-situ ^{1}H NMR spectra following reaction of **1**^{Cy} + 2 propanediol to form **3**^{Cy} + 4 ^{i}PrOH by alkoxide exchange

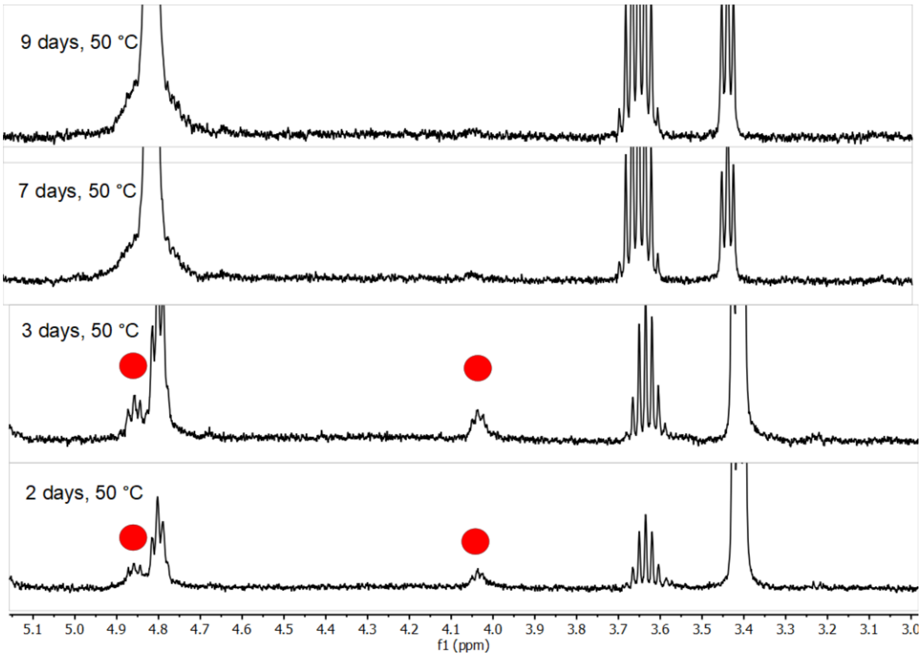

Figure S22. Zoomed section of in-situ ^{1}H NMR spectra following reaction of $\mathbf{1}^{Cy}$ + 2 propanediol to form $\mathbf{3}^{Cy}$ + 4 ^{i}PrOH by alkoxide exchange. Red dots indicate triplet signals (4.03 and 4.86) consistent with a monocoordinated Ti-OC**H**$_2$CH$_2$C**H**$_2$-OH unit.

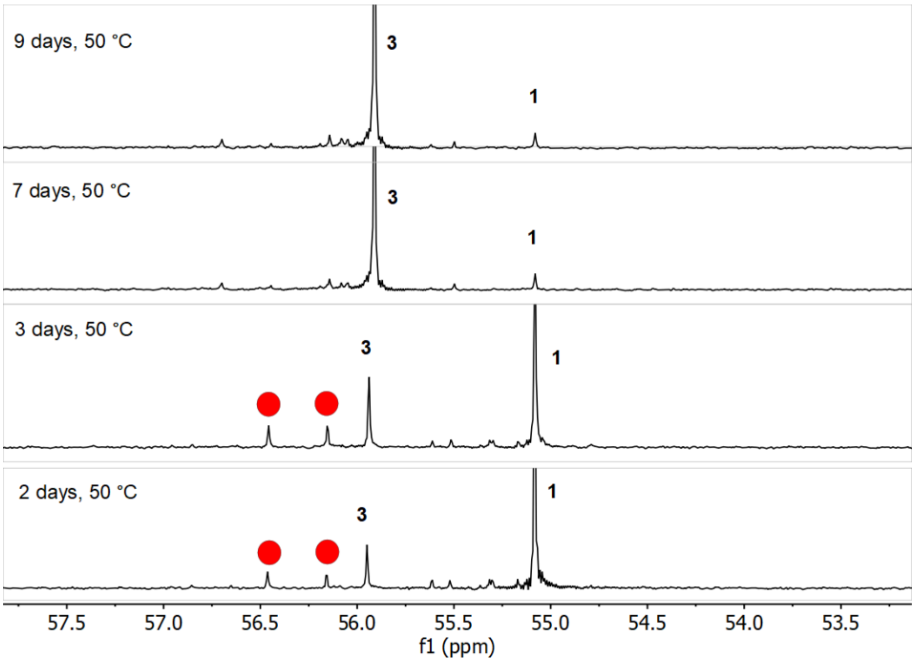

Figure S23. In-situ ^{31}P {^{1}H} NMR spectra following reaction of $\mathbf{1}^{Cy}$ + 2 propanediol to form $\mathbf{3}^{Cy}$ + 4 ^{i}PrOH by alkoxide exchange. Red dots (56.1 & 56.4 ppm) are consistent with the symmetry of a tetrameric cluster with one unique alkoxide, and thus with a formula of $[Ti_4O_4(O_2PCy_2)_4(OR^1)_3(OR^2)]$ (i.e. OR^1 = O^{i}Pr; OR^2 = O(CH$_2$)$_3$-OH, $\mathbf{3a}^{Cy}$).

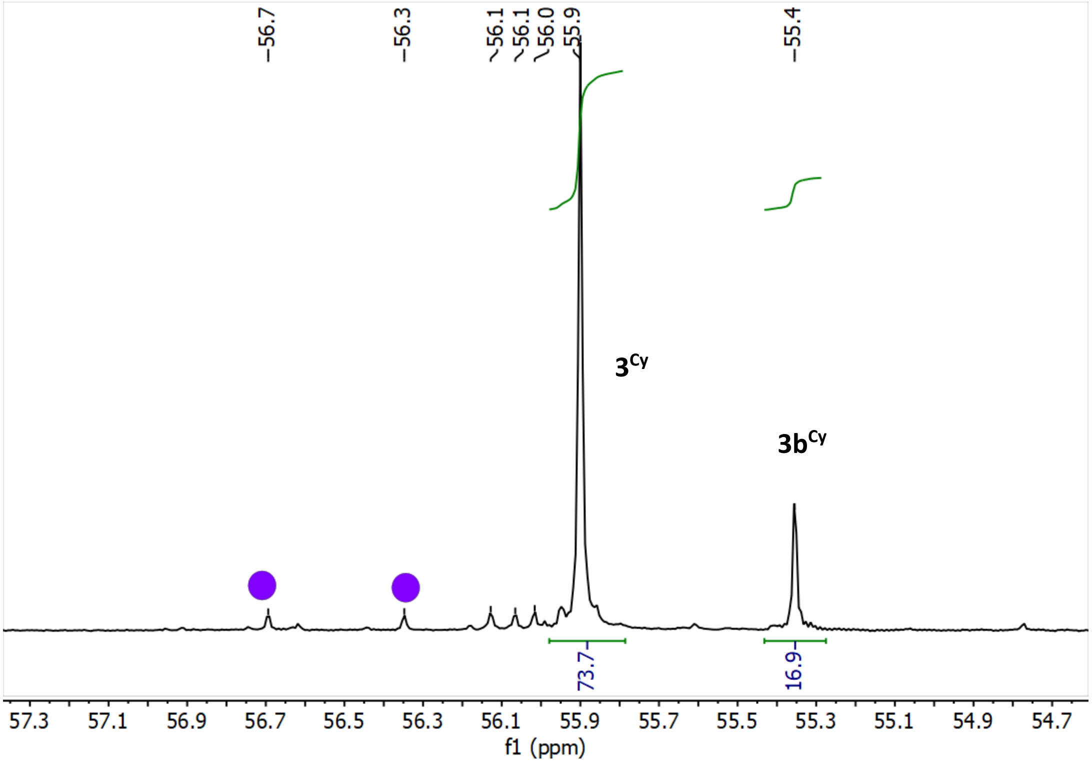

Figure S24. ^{31}P {^{1}H} NMR spectra of reaction of **3**^{Cy} with 30 equivalents ^{i}PrOH after three days. The Signal labelled **3b**^{Cy} is distinct from **1**^{Cy} and **3**^{Cy} and is consistent with a cluster such as $[Ti_4O_4(O_2PCy_2)_4\{O(CH_2)_3O\}(O^iPr)_2]$. The purple dots are consistent with a Ti_4 cluster with two different alkoxides on one face, perhaps **3c**^{Cy}, expected to be an intermediary species in this process.

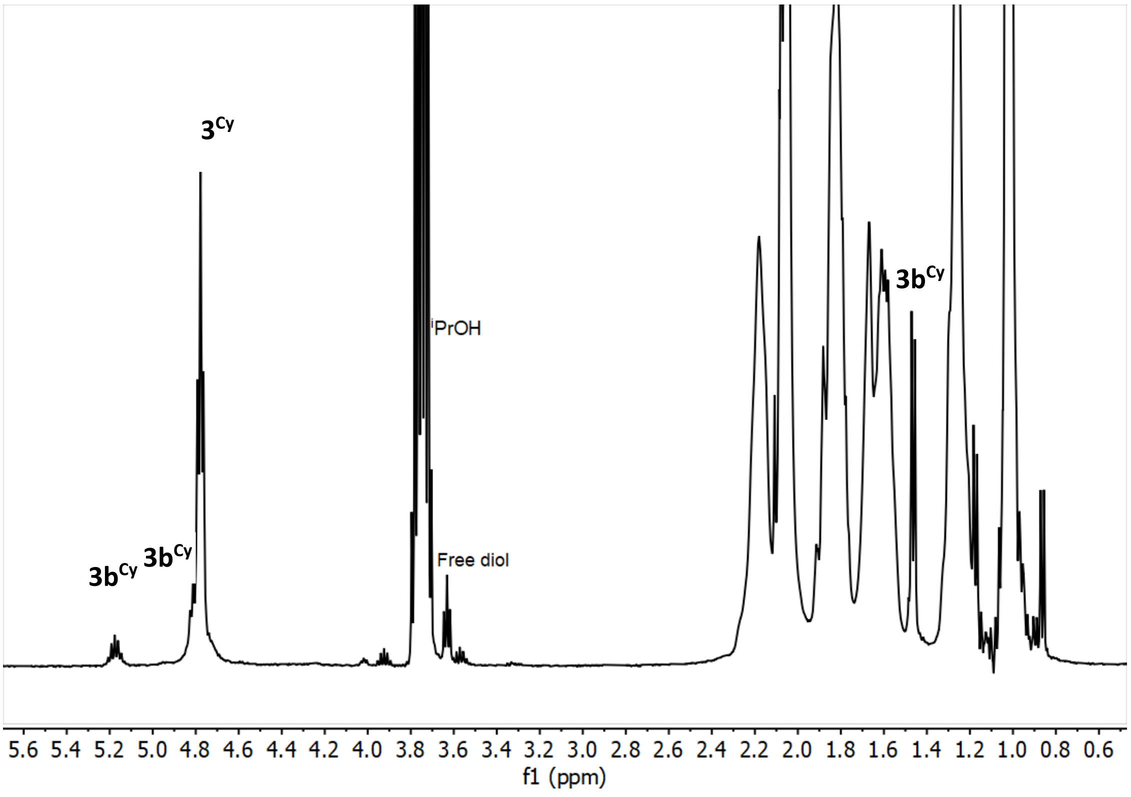

Figure S25. ^{1}H NMR spectra of reaction of **3**^{Cy} with 30 equivalents ^{i}PrOH after three days. Signals labelled for **3b**^{Cy} are consistent with a cluster $[Ti_4O_4(O_2PCy_2)_4\{O(CH_2)_3O\}(O^iPr)_2]$.

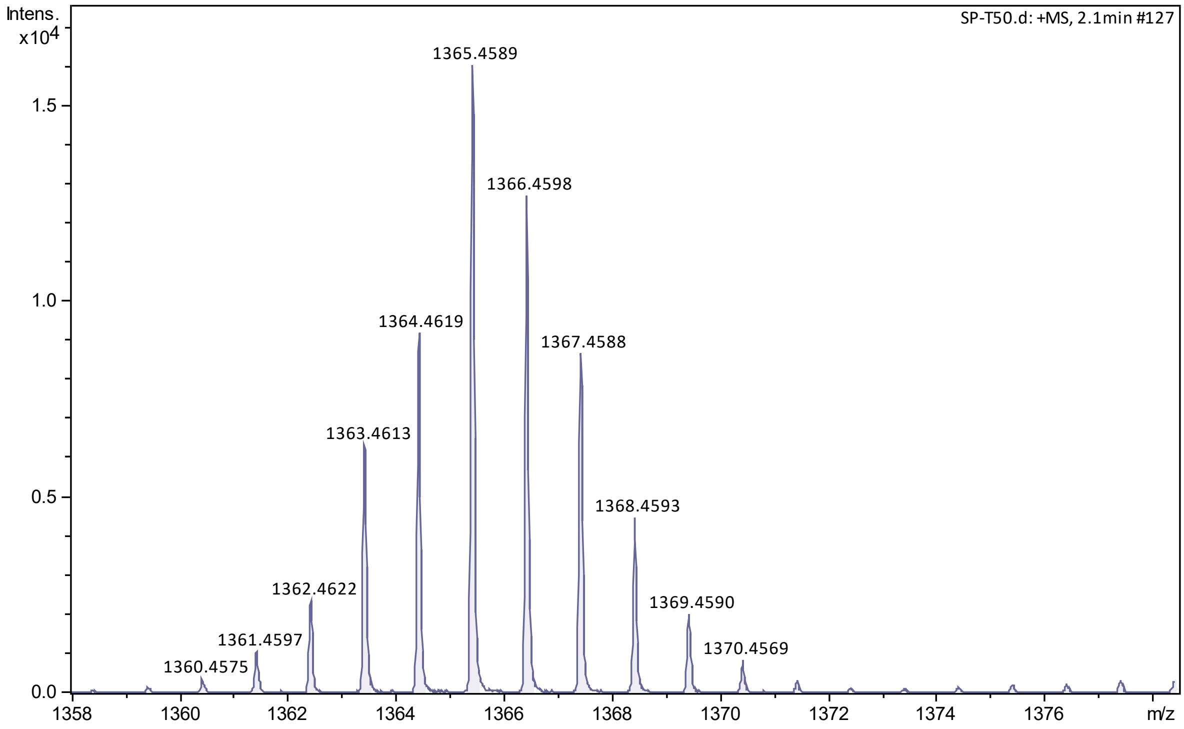

Figure S26. ESI mass spectrometry data for **3b**^{Cy}+H$^+$ in CH_2Cl_2 (as a mixture with **3**^{Cy}). Solution was prepared by mixing **3**^{Cy} (3 mg, 2.3 µM) with 57 equiv. HO^{i}Pr (10 µL, 0.13 mM) in 1.5 mL CH_2Cl_2.

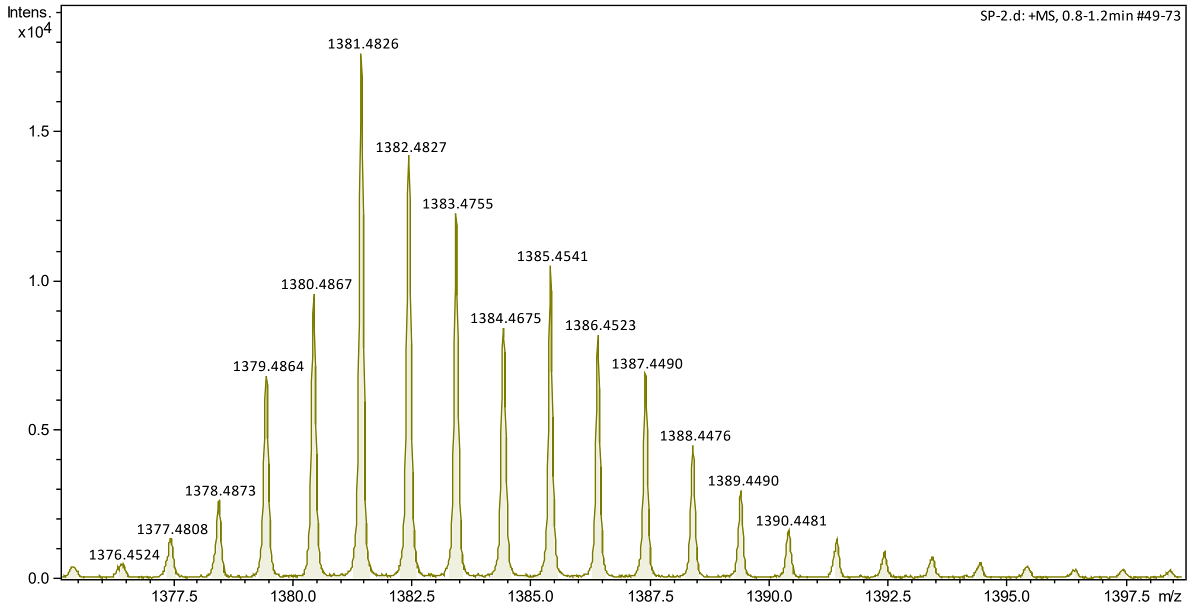

Figure S27. ESI mass spectrometry data for **3c**^{cy}+H$^+$ observed (predicted = 1381.45) from analysis of a solution of **1**^{cy} + 1~equiv. 1,3-propanediol after three days mixing in CH_2Cl_2 at room temperature. A second isotope pattern of an unknown complex overlaps with this signal.

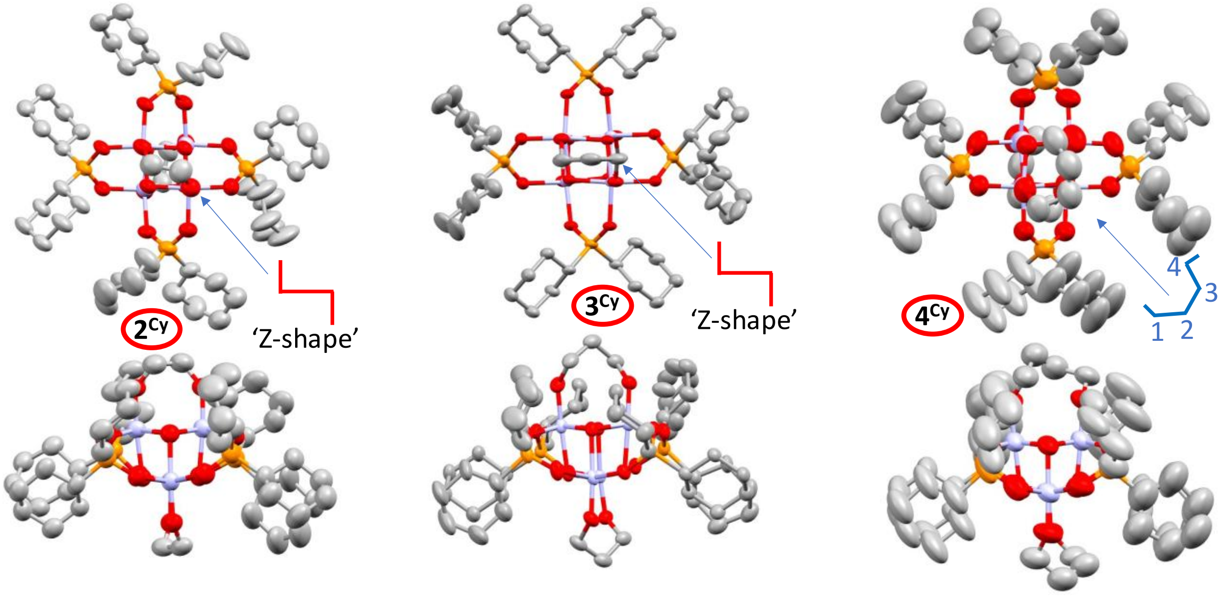

Figure S28. Single crystal solid-state structures of $\mathbf{2^{Cy}}$, $\mathbf{3^{Cy}}$ & $\mathbf{4^{Cy}}$ displayed from two different angles, ellipsoids shown with 50% probability. Hydrogen atoms omitted for clarity.

Table S1. Bond lengths from X-ray crystal structures comparison table. Key to show bonds above. *X-ray diffraction data for **2**^Ph^ is low quality and the accuracy of bond distances should be considered accordingly.

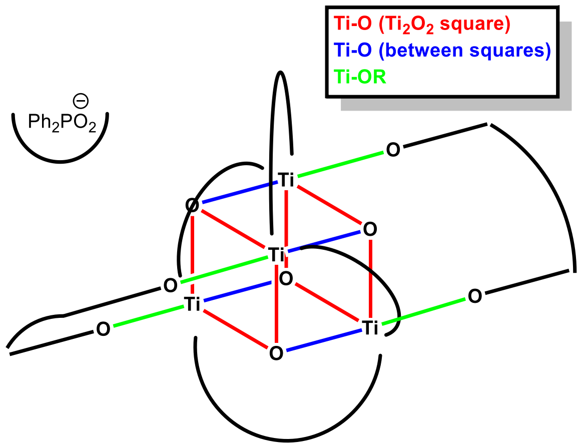

Bond length (Å)	$\mathbf{1}^{Ph}$ [1] (monodentate alkoxides)	$\mathbf{2}^{Ph}$ *	$\mathbf{3}^{Ph}$	$\mathbf{4}^{Ph}$	$\mathbf{5}^{Ph}$
Ti-O (Ti_2O_2 square)	Range: 1.9083(19) - 1.9448(18)	Range: 1.878(1) – 1.967(2)	Range: 1.910(2) - 1.9412(19)	Range: 1.893(2) – 1.951(2)	Range: 1.926(2) – 1.977(2)
Ti-O (between squares)	Range: 2.121(2)-2.159(2)	Range: 2.027(1) – 2.131(1)	Range: 2.0898(19) - 2.1350(19)	Range: 2.104(1) – 2.161(1)	Range: 2.063(2) – 2.145(2)
Ti-OP	Range: 2.014(2)-2.057(2)	Range: 1.974(2) – 2.070(2)	Range: 2.002(2) - 2.038(2)	Range: 1.997(2) – 2.067(2)	Range: 1.997(2) – 2.067(2)
Ti-OR	Range: 1.773(2) - 1.794(2)	Range: 1.792(1) – 1.827(1)	Range: 1.785(2) - 1.808(2)	Range: 1.768(2) – 1.801(1)	Range: 1.760(3) – 1.781(2)

Bond length (Å)	$\mathbf{1}^{Cy}$ [1] (monodentate alkoxides)	$\mathbf{2}^{Cy}$	$\mathbf{3}^{Cy}$	$\mathbf{4}^{Cy}$
Ti-O (Ti_2O_2 square)	1.912(2) and 1.937(2)	Range: 1.909(4) – 1.964(4)	1.924(2) and 1.932(2)	Range: 1.915(5) – 1.946(4)
Ti-O (between squares)	2.121(2)	Range: 2.103(5) – 2.129(5)	2.100(2) and 2.1273(18)	2.097(5) and 2.104(5)
Ti-OP	2.011(3) and 2.021(3)	Range: 1.982(6) – 2.033(6)	Range: 1.9962(16) - 2.0266(18)	Range: 2.001(6) – 2.021(6)
Ti-OR	1.790(2)	Range: 1.810(5) – 1.818(5)	1.804(2) and 1.8158(18)	1.784(7) and 1.812(7)

Table S2. Bond angles comparison table. Key to show angles above. *X-ray diffraction data for **2**^{Ph} is low quality and the accuracy of bond angles should be considered accordingly.

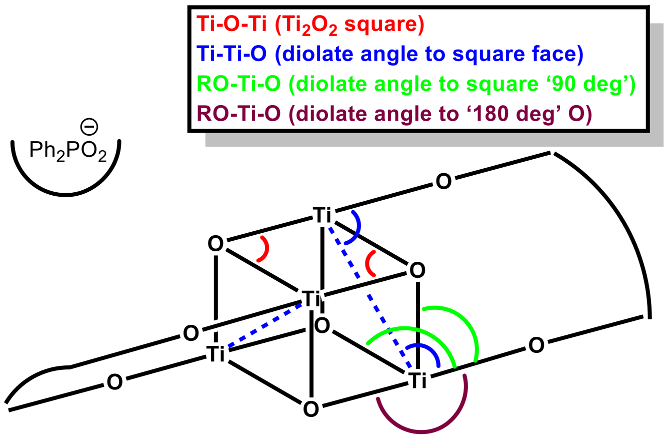

Bond angle (°)	**1**^{Ph} [1] (monodentate alkoxides)	**2**^{Ph} *	**3**^{Ph}	**4**^{Ph}	**5**^{Ph}
Ti-O-Ti (Ti_2O_2 square)	Range: 97.01(1) - 98.33(1)	Range: 95.99(6) - 99.26(6)	Range: 96.39(8) - 97.66(8)	Range: 96.48(7) – 98.00(7)	Range: 94.72(1) – 101.01(1)
Ti-Ti-O (diolate/alkoxide angle to square face)	Range: 95.77(7) – 99.97(7)	Range: 89.76(4) – 91.50(5)	Range: 91.20(8) – 94.44(8)	Range: 93.48(6) – 96.14(6)	Range: 96.48(8) – 101.83(8)
RO-Ti-O (diolate/alkoxide angle to square '90 deg')	Range: 97.63(9) – 103.48(9)	Range: 94.46(6) – 99.23(6)	Range: 92.76(1) – 102.72(1)	Range: 97.41(7) – 103.26(7)	Range: 99.08(1) – 104.40(1)
RO-Ti-O (diolate/alkoxide angle to '180 deg' O)	Range: 174.73(9) – 176.46(9)	Range: 174.50(6) – 175.80(6) 'inwards'	Range: 172.36(1) – 174.98 'sideways'	Range: 175.50(7) – 178.73(7) 'sideways'	Range: 170.66(1) – 176.70(1) 'sideways'

Bond angle (°)	**1**^{Cy} [1] (monodentate alkoxides)	**2**^{Cy}	**3**^{Cy}	**4**^{Cy}
Ti-O-Ti (Ti_2O_2 square)	Range: 98.25(10) – 99.16(10	Range: 9.811(2) – 9.875(2)	98.30(7) and 98.61(7)	98.58(2) – 98.69(2)
Ti-Ti-O (diolate/alkoxide angle to square face)	97.82(9)	Range: 90.31(2) – 91.51(2)	92.78(6) and 93.09(6)	93.85(2) and 94.84(2)
RO-Ti-O (diolate/alkoxide angle to square '90 deg')	Range: 101.17(1) – 101.32(1)	Range: 94.67(2) – 97.70(2)	Range: 93.90(8) – 100.92(8)	Range: 96.49(3) - 99.97(2)
RO-Ti-O (diolate/alkoxide angle to '180 deg' O)	177.82(1)	Range: 174.17(2) – 175.88(2) 'inwards'	Range: 173.27(8) and 175.39(8) 'sideways'	Range: 176.18(3) – 178.33(3) 'sideways'

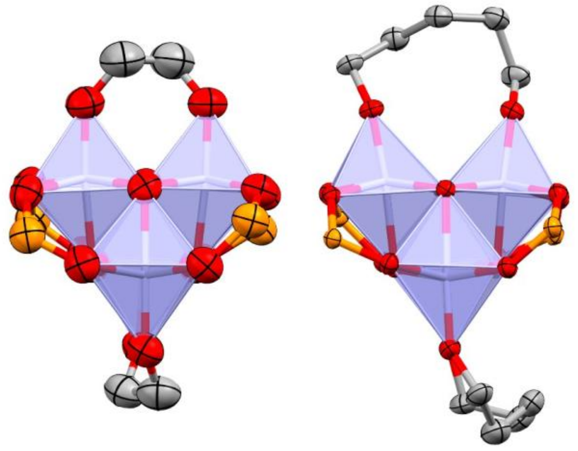

Figure S29. Crystal structures of 2^{Cy} and 5^{Ph} (phosphinate C and all H atoms omitted for clarity), displaying the Ti octahedral geometries.

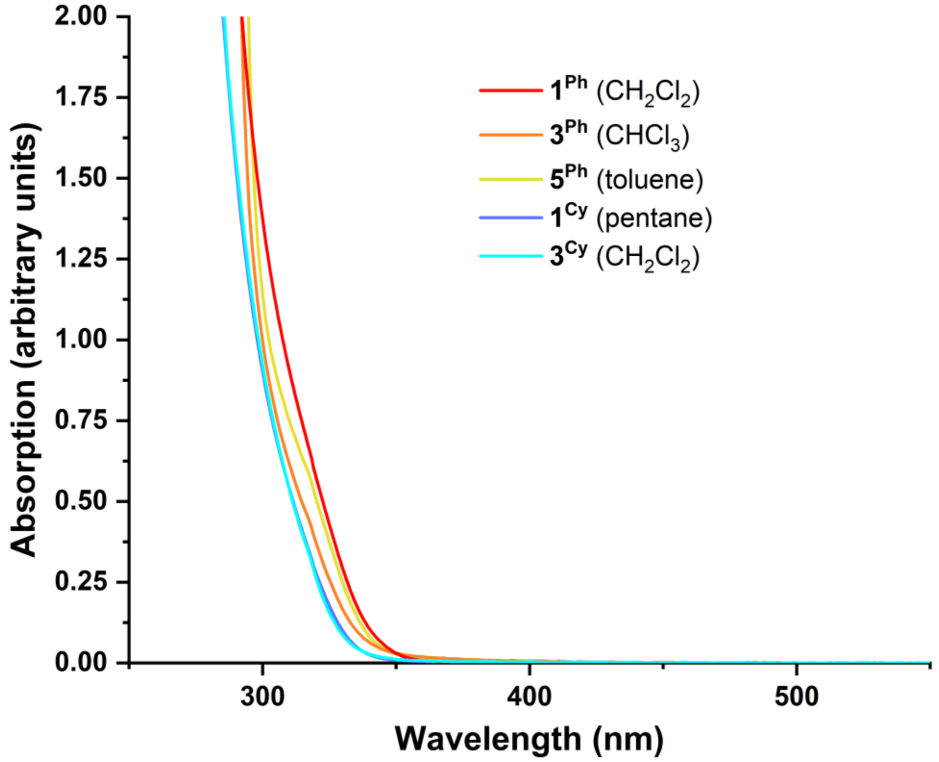

Figure S30. UV spectra of bidentate alkane diolate compounds 1^{Ph}, 3^{Ph} and 5^{Ph} in comparison to monodentate alkoxide complexes 1^{Cy} and 3^{Cy}, concentration 0.44 mM ([Ti] = 1.75 mM).

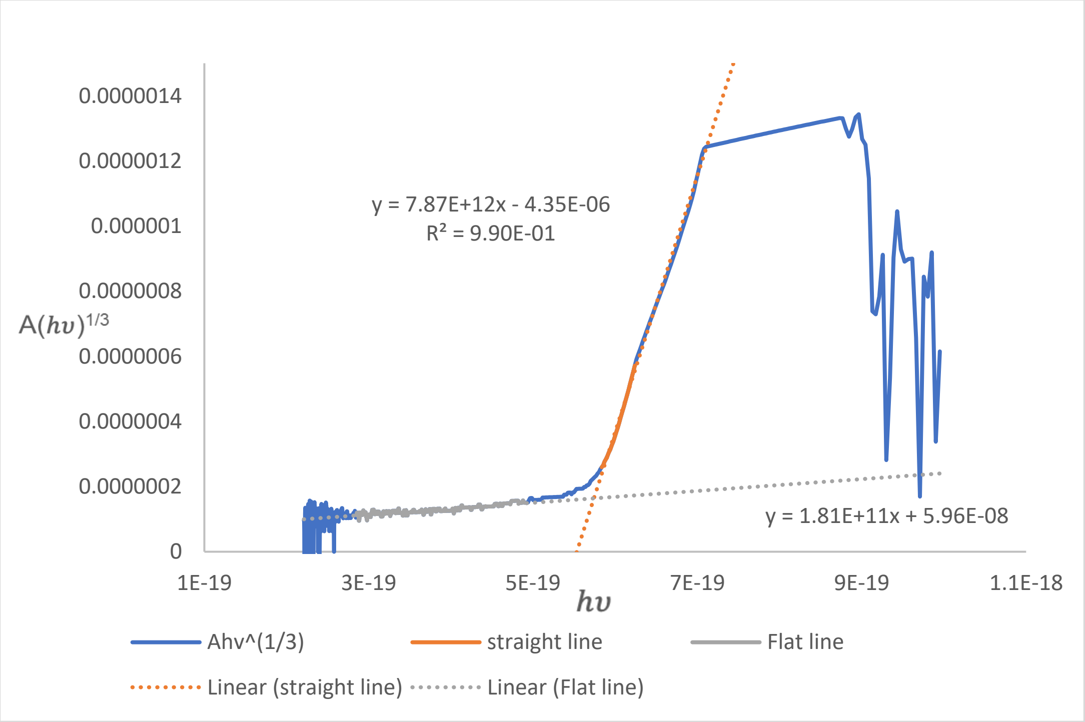

Figure S31. Tauc's plots of absorption onset for **3**^{Cy} at 0.44 mM. X-value of intercept of linear section with linear baseline extrapolation used for value of absorption onset. Intercepts correspond to energies of 3.58 eV.

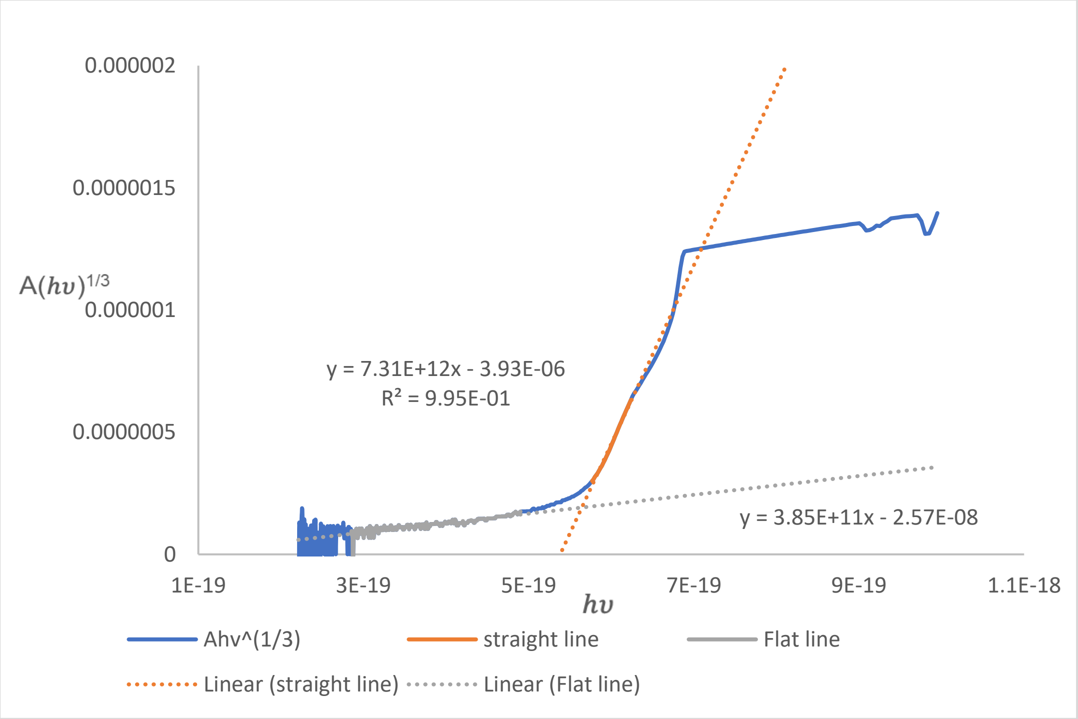

Figure S32. Tauc's plots of absorption onset for **3**^{Ph} at 0.44 mM. X-value of intercept of linear section with linear baseline extrapolation used for value of absorption onset. Intercepts correspond to energies of 3.52 eV.

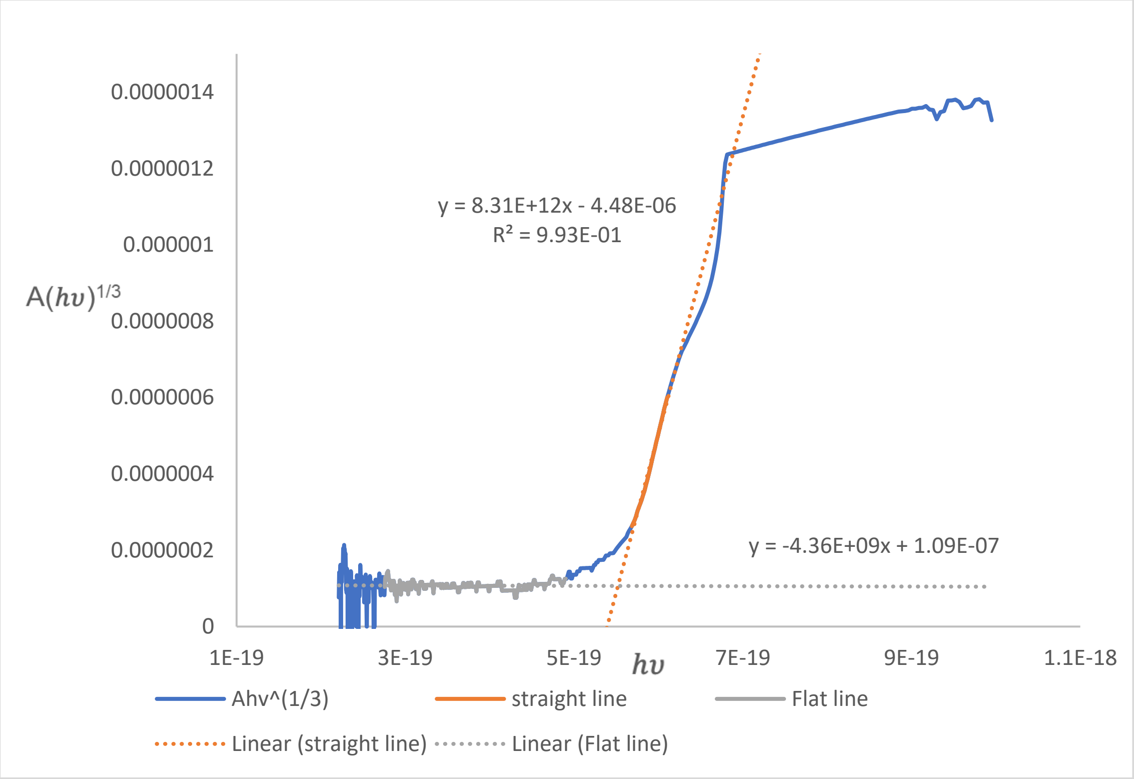

Figure S33. Tauc's plots of absorption onset for **5**^{Ph} at 0.44 mM. X-value of intercept of linear section with linear baseline extrapolation used for value of absorption onset. Intercepts correspond to energies of 3.45 eV.

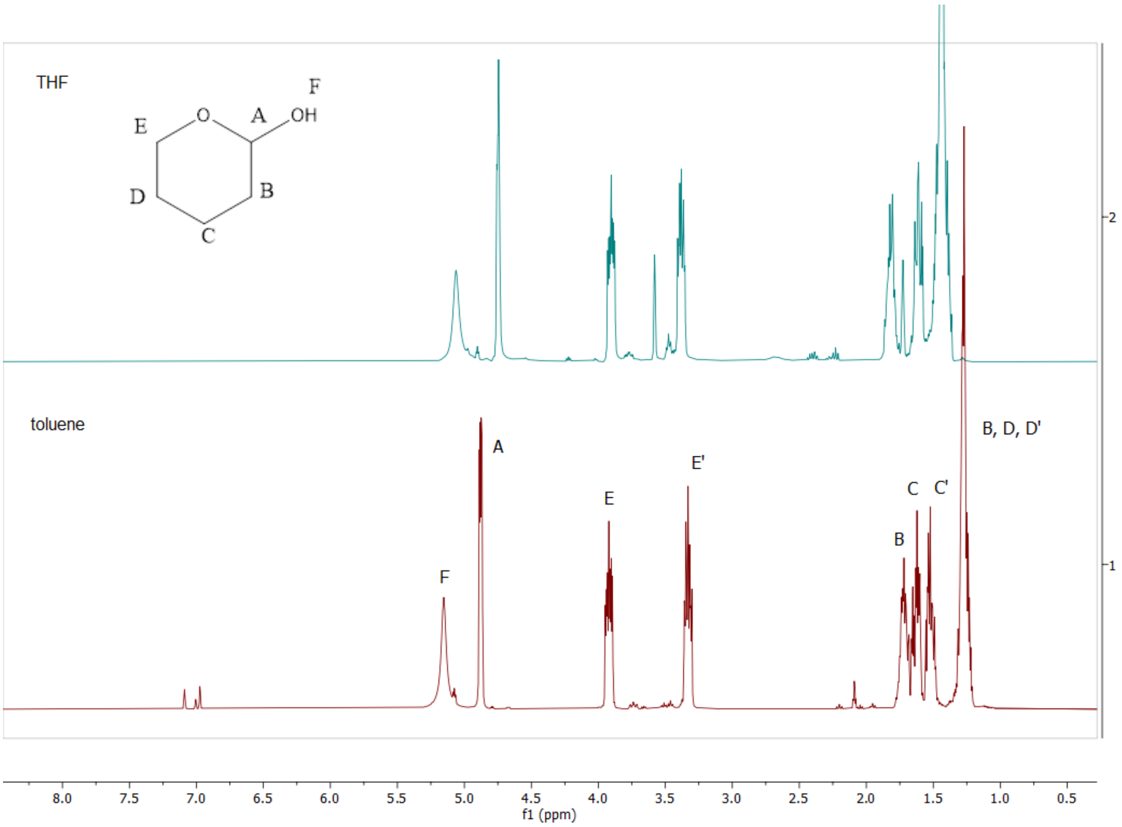

Figure S34. ^{1}H NMR spectra of commercial sample of tetrahydro-2H-pyran-2-ol in d_8-THF (above) and d_8-toluene (below)

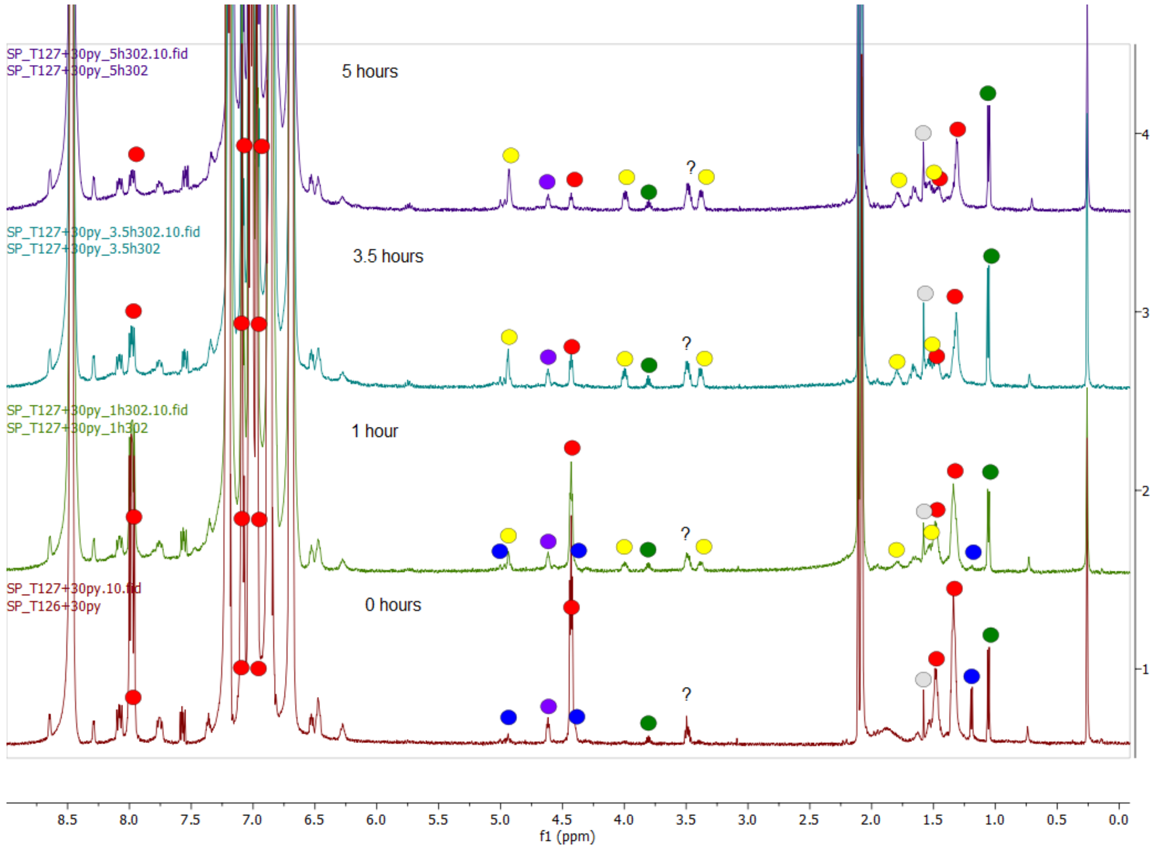

Fig S35. ^{1}H NMR spectra from a d^{8}-toluene solution of $\mathbf{5^{Ph}}$ (red dots) with 30 equiv. of pyridine under 302 nm UV light (time = 0, 1, 3.5, 5 hours bottom to top) showing the formation of ~1 equiv. of tetrahydro-2H-pyran-2-ol (yellow dots). Traces of 4-pentene-1-ol are observed as indicated by multiplet peak at 5.7 ppm. A small quantity of byproduct $\mathbf{5b^{Ph}}$ was also present (blue dots) and also underwent photoredox reaction to generate 1:1:1 equiv. of $\mathbf{5^{Ph}}$**-red**, ^{i}PrOH (green dots) and acetone (grey dots) (N.B. small quantities of acetone and ^{i}PrOH were present in the solution before photoreaction). A minor unknown impurity with an associated ^{31}P signal is labelled with a purple dot, this signal decreased slowly (~33% loss) over 5 hours. An unknown organic signal is labelled with a '?', this likely overlaps with the enol product after the photoreaction. Other peaks are accounted for from solvents, grease or internal standard (capillary of PPh_3 in C_6D_6).

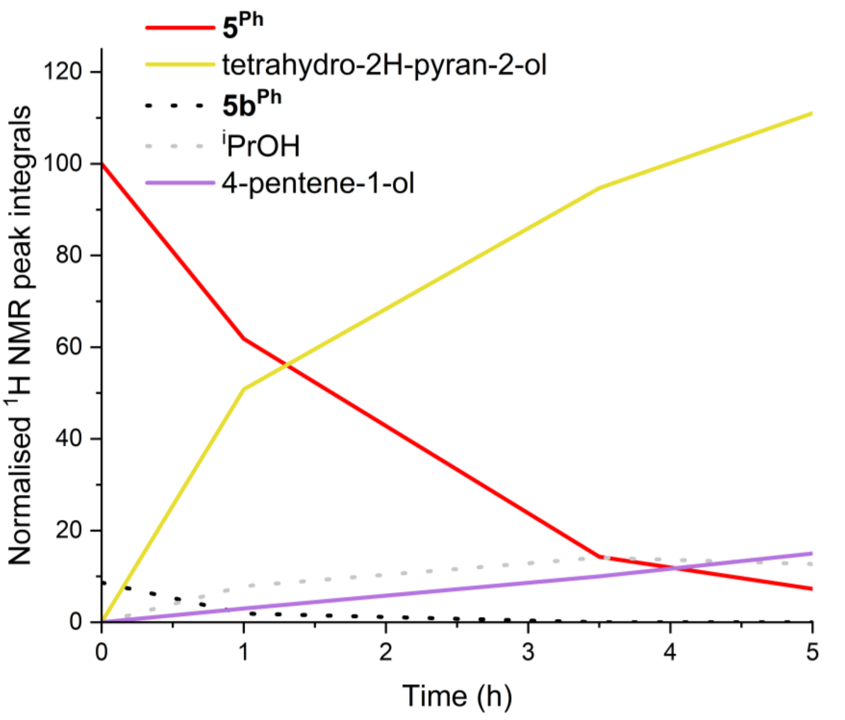

Fig S36. Normalised ^{1}H NMR peak integrals from a d^{8}-toluene solution of $\mathbf{5^{Ph}}$ under 302 nm UV light (least obscured peaks were used for integral comparison). A small quantity of byproduct $\mathbf{5b^{Ph}}$ was also present and also underwent photoredox reaction to generate 1:1:1 equiv. of $\mathbf{5^{Ph}}$**-red**, $^{i}PrOH$ and acetone (N.B. acetone integral was obscured by overlapping signals).

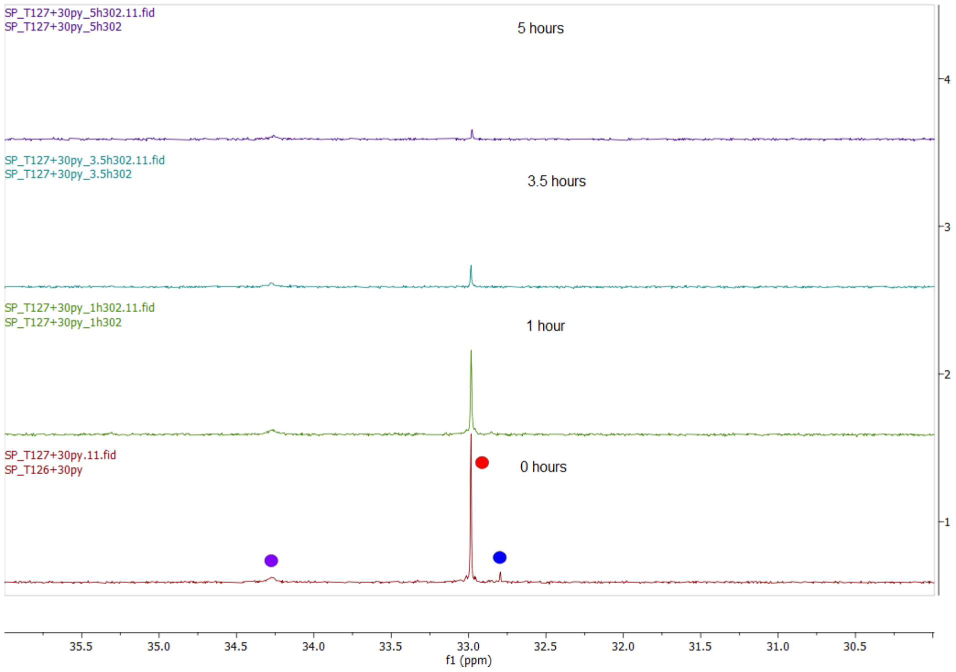

Figure S37. $^{31}P\{^{1}H\}$ NMR spectra from a d^{8}-toluene solution of $\mathbf{5^{Ph}}$ (red dot) with 30 equiv. of pyridine under 302 nm UV light (time = 0, 1, 3.5, 5 hours bottom to top). A small quantity of byproduct $\mathbf{5b^{Ph}}$ was also present (blue dot) and also underwent photoredox reaction. A minor unknown impurity is labelled with a purple dot, this signal decreased slowly (~33% loss) over 5 hours. Integrals collected against an internal standard (capillary of PPh_3 in C_6D_6).

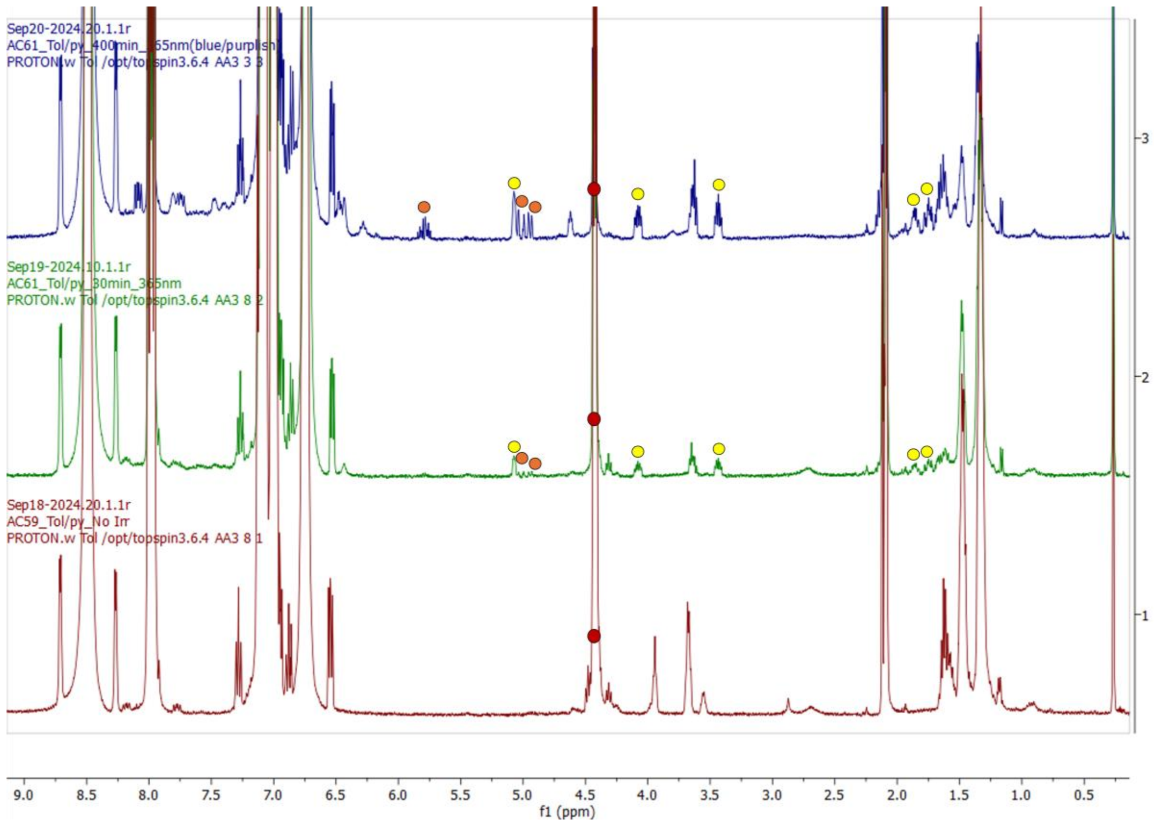

Figure S38: ^{1}H NMR spectra from a d^{8}-toluene solution of **5^{Ph}** (red dots) with 30 equiv. of pyridine under 365 nm UV light (time = 0, 30, 400 minutes bottom to top) showing the formation of 4-pentene-1-ol (orange dots) and tetrahydro-2H-pyran-2-ol (yellow dots).

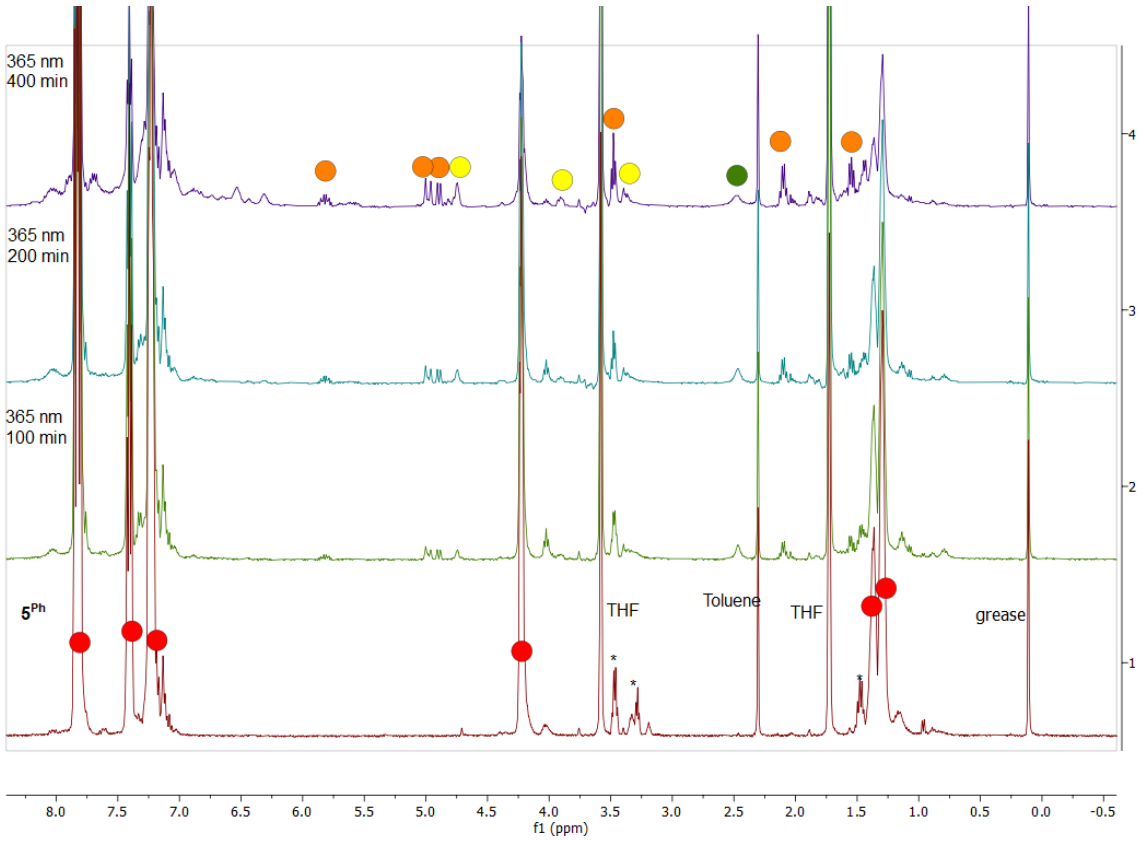

Figure S39. ¹H NMR spectra from a d⁸-THF solution of **5^Ph^** (red dots) under 365 nm UV light (time = 0, 100, 200, 400 minutes, bottom to top) showing the formation of 4-pentene-1-ol (orange dots) and tetrahydro-2H-pyran-2-ol (yellow dots), broad signal indicative of OH environment shown with green dot. Minor unknown impurities labelled with a '*'. Other peaks are accounted for from solvents and grease. Increased signals around phenyl region, at end of process may correlate with photoproduct clusters, which may include $[Ti_4O_4(O_2PPh)_6]$.

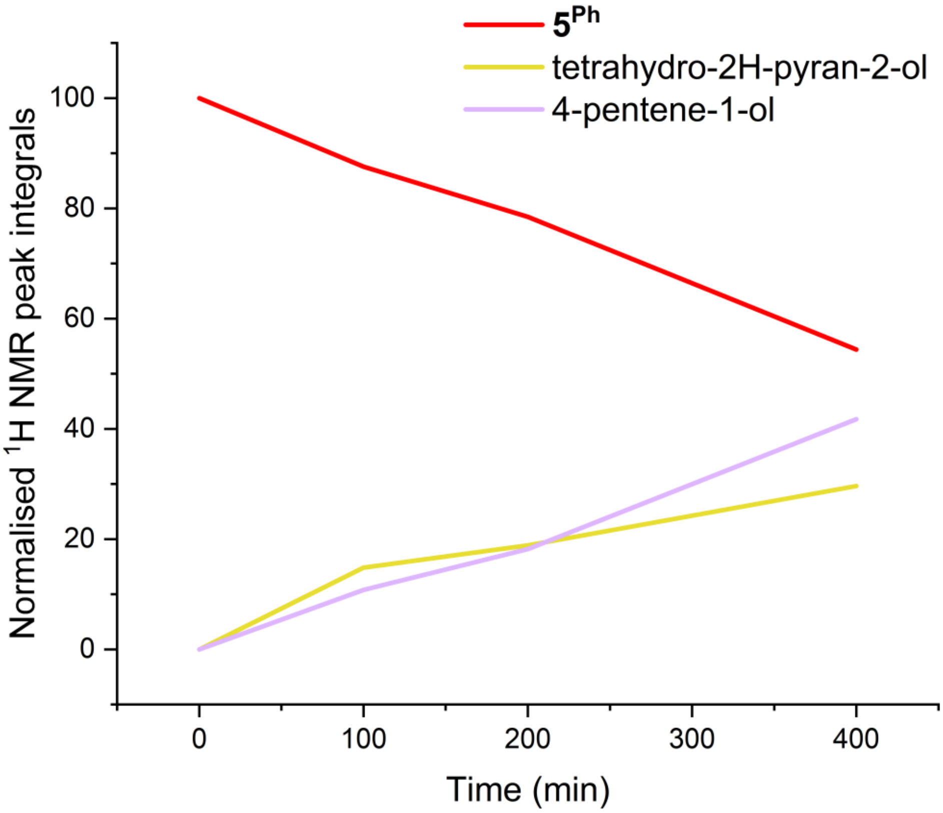

Fig S40. Normalised ^{1}H NMR peak integrals from a d^{8}-THF solution of **5^{Ph}** under 365 nm UV light (least obscured peaks were used for integral comparison).

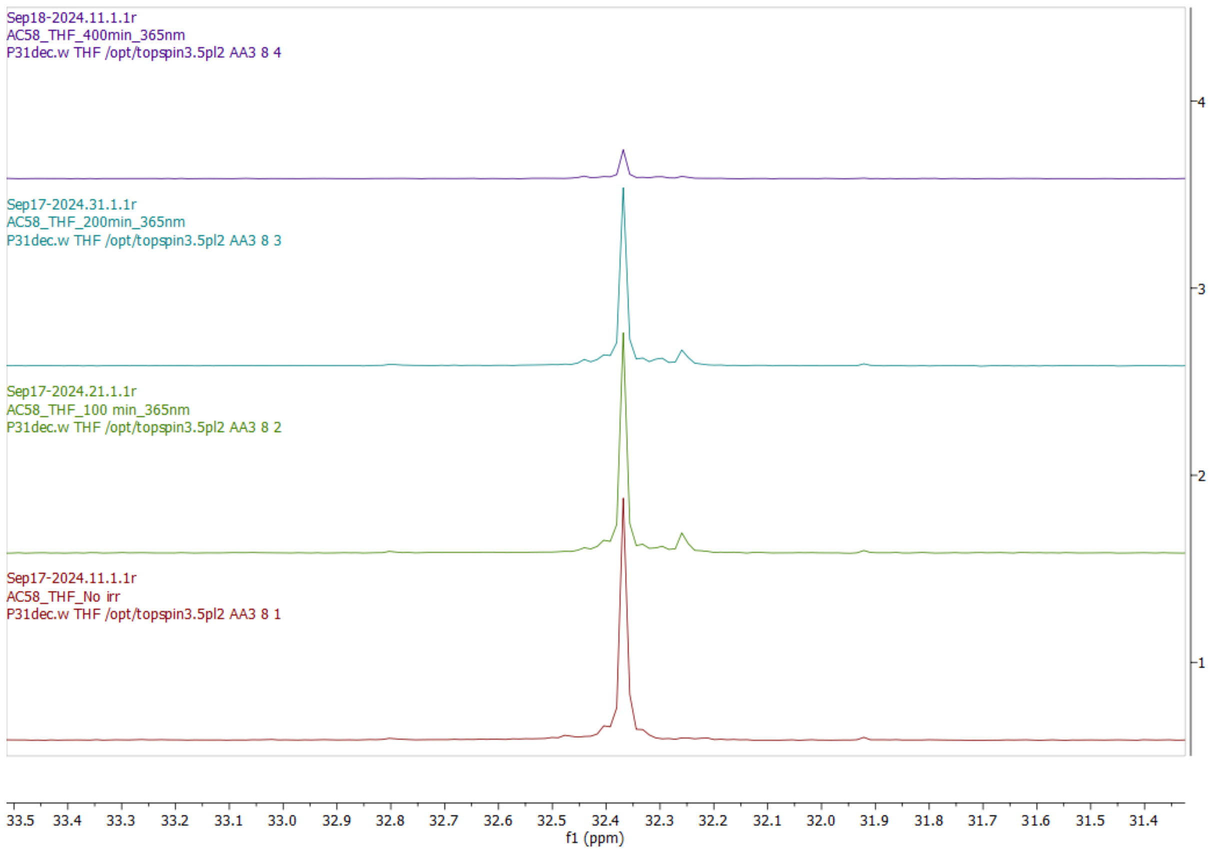

Figure S41. Progress of reaction of $\mathbf{5}^{\mathbf{Ph}}$ dissolved in THF under 365 nm light monitored by $^{31}P\{^1H\}$ NMR spectroscopy.

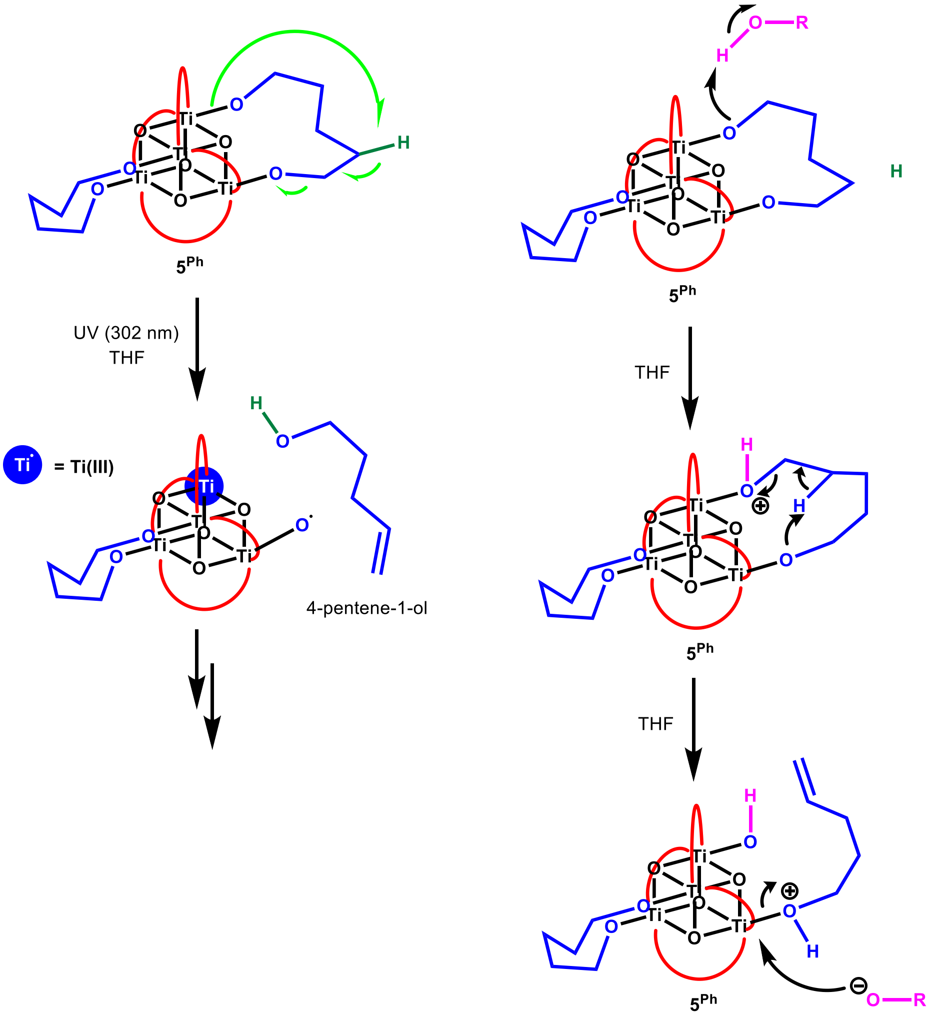

Figure S42. Possible photoreaction mechanisms to generate 4-pentene-1-ol from **5**^{Ph}. Left hand side, photochemical pathway leading to diradical cluster prone to onward reactions. Right hand side, non-redox pathway facilitated by protic species. Note that alcohols (lactols) are produced via the photoredox pathway for **5**^{Ph} under UV light (see Fig S40).

Table S3. Photoproducts of the irradiation of **5**^{Ph} in different solvents with 365 or 302 nm light by analysis of ^{1}H NMR integrals. Note that estimated equivalents of organic photoproducts gives values slightly above 100% mass balance, likely due to error of integral analysis.

Solvent	Irradiation (nm)	Time (min)	Consumption **5**^{Ph} %	Lactol % equiv.	4-pentene-1-ol % equiv.
Toluene + 30 equiv. pyridine	365	400	64	51	14
Toluene + 30 equiv. pyridine	302	400	72	70	16
Toluene + 30 equiv. pyridine (repeat)	302	300	93	119	16
THF	365	400	46	30	43
THF	302	720	78	45	63

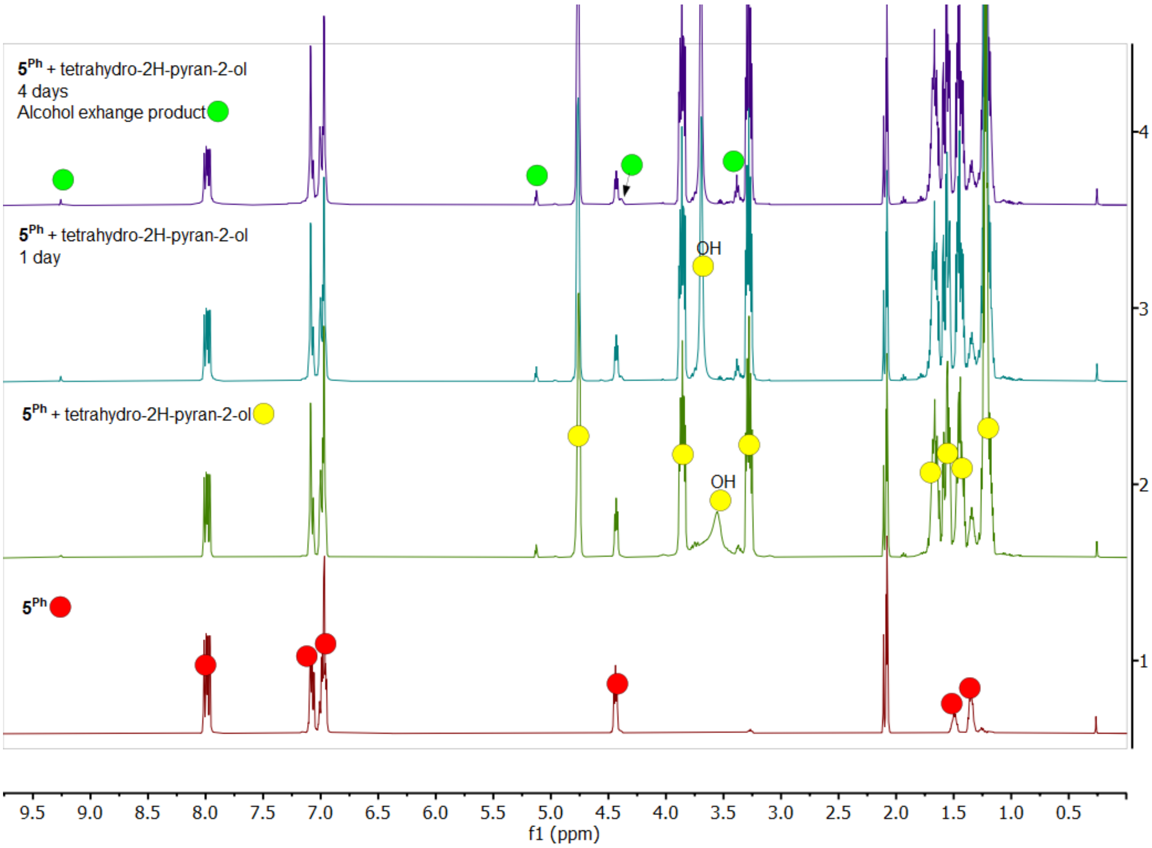

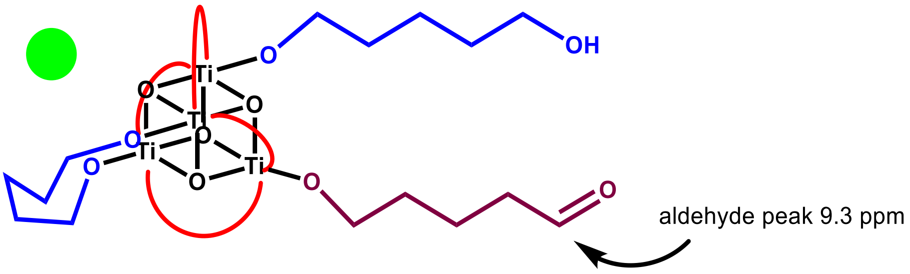

Fig S43. ^{1}H NMR spectra of **5**^{Ph} (red dots) and after addition of 30 equiv. of tetrahydro-2H-pyran-2-ol (yellow dots). Evidence of minor species (green dots) growing in slowly, with suggested structure shown below, likely via alcohol exchange of the open form of the lactol.

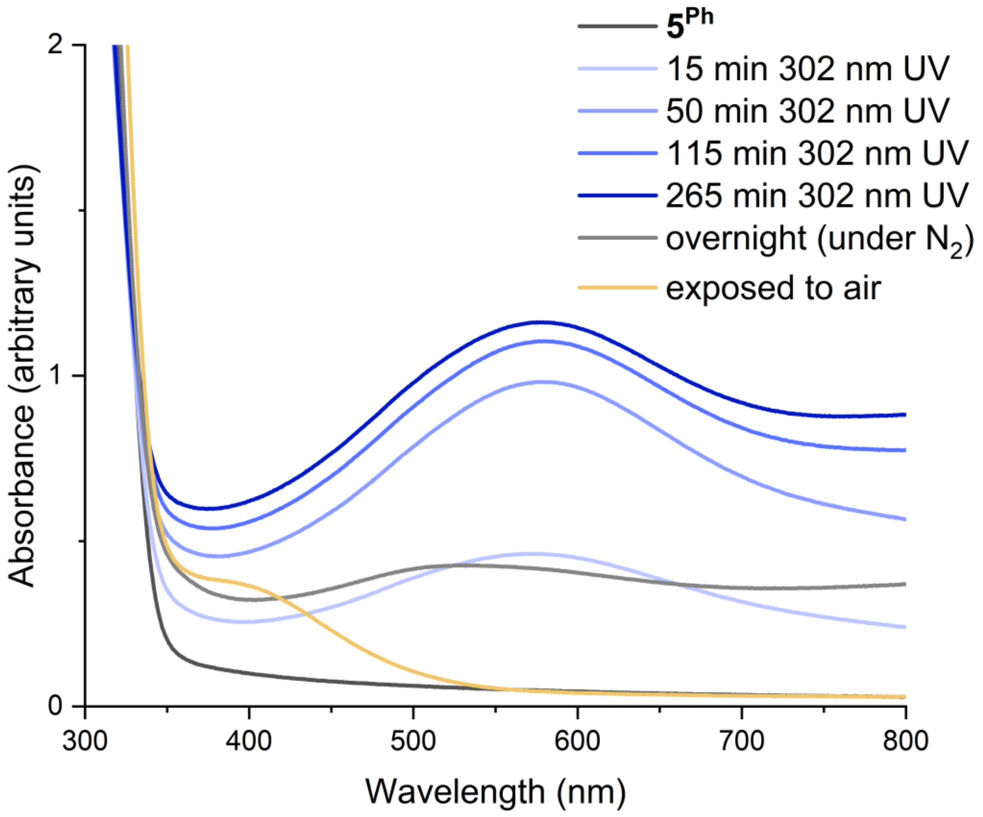

Figure S44. UV-vis spectra of **5**^{Ph} in toluene (with 30 equiv. pyridine) under irradiation with 302 nm light, then aged overnight under N_2 atmosphere and finally exposed to air. [**5**^{Ph}] ~ 1.25 mM, [Ti] ~ 5 mM

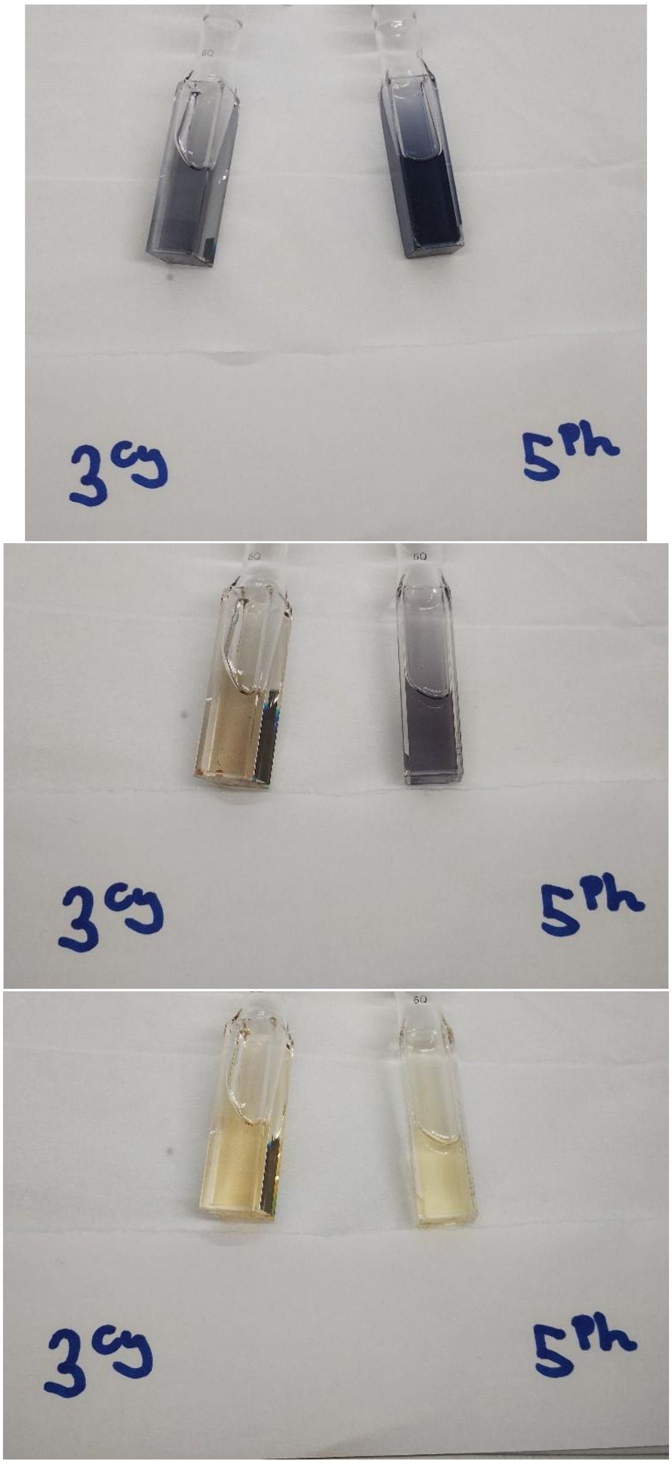

Figure S45. Photos of irradiated solutions of $\mathbf{3}^{Cy}$ and $\mathbf{5}^{Ph}$ after 265 minutes under 302 nm light (top), aged overnight under N_2 (middle) and finally exposed to air (bottom).

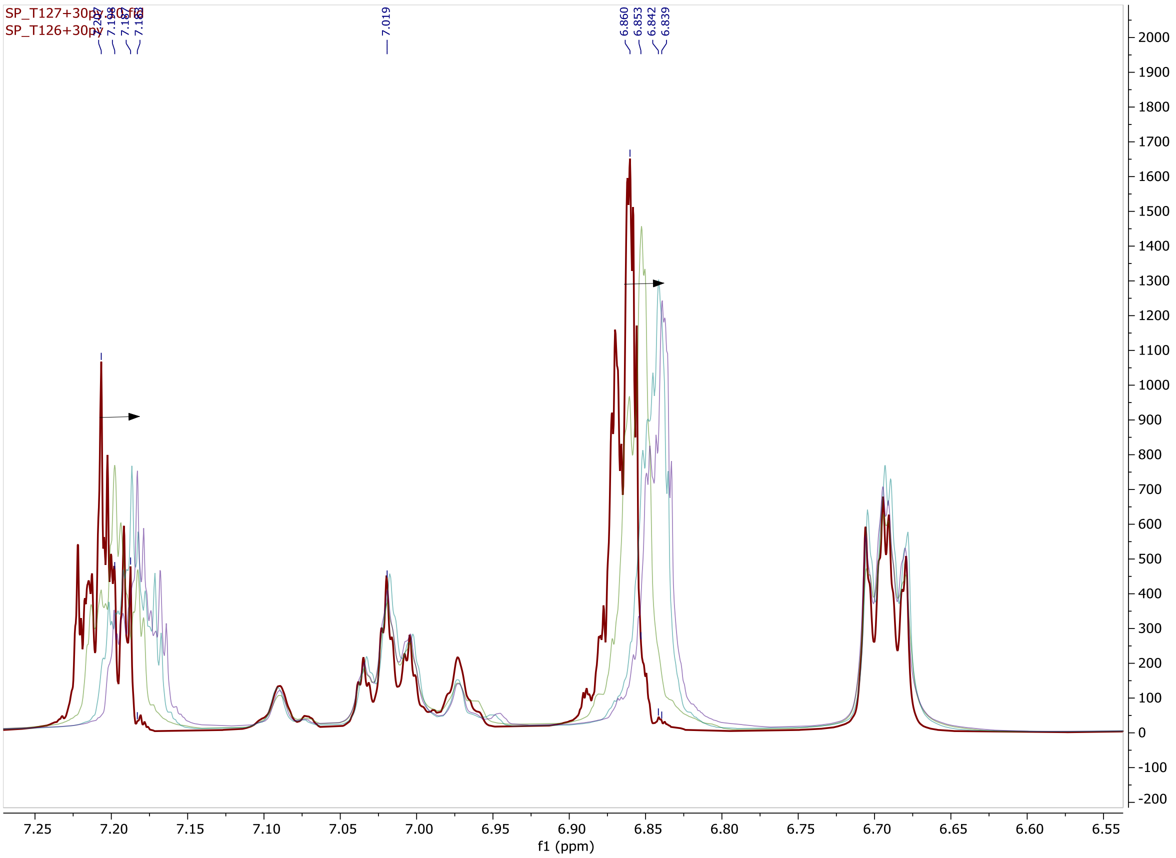

Figure S46. Aromatic region of ^{1}H NMR spectra of a solution of $\mathbf{5^{Ph}}$ undergoing UV irradiation (4 time points over 5 hours displayed). Stationary peaks are from toluene solvent molecules in solution whilst peaks that appear to shift are from within a sealed capillary of PPh_3 in C_6D_6. The apparent shift is caused by a magnetisation of the main solution by the growth of paramagnetic species, this shift can give an estimate of the no. of unpaired electrons per molecule by the Evans NMR method.[19]

Using equations

$\mu_{eff} = \sqrt{((8 \times 3T \times \Delta_{ppm})/(4\pi \times 10^{6} \times c))}$

$\mu_{eff} = \sqrt{n(n+2)}$

where T = temperature (K), Δ_{ppm} is the difference in chemical shift and c = concentration (mol/mL) and any diamagnetic contribution is disregarded. n = number of unpaired electrons per molecule.

The calculated μ_{eff} ~1.1 BM per cluster of $\mathbf{5^{Ph}}$**-red**, corresponds to an average value of ~0.5 unpaired electrons per cluster. This value may be explained by a combination of (essentially degenerate) triplet and singlet states of $\mathbf{5^{Ph}}$**-red** in solution, or by the presence of a combination of different photoproducts with low solubility or different magnetic properties (e.g. $[Ti_4O_4(O_2PPh_2)_6]$).[1]

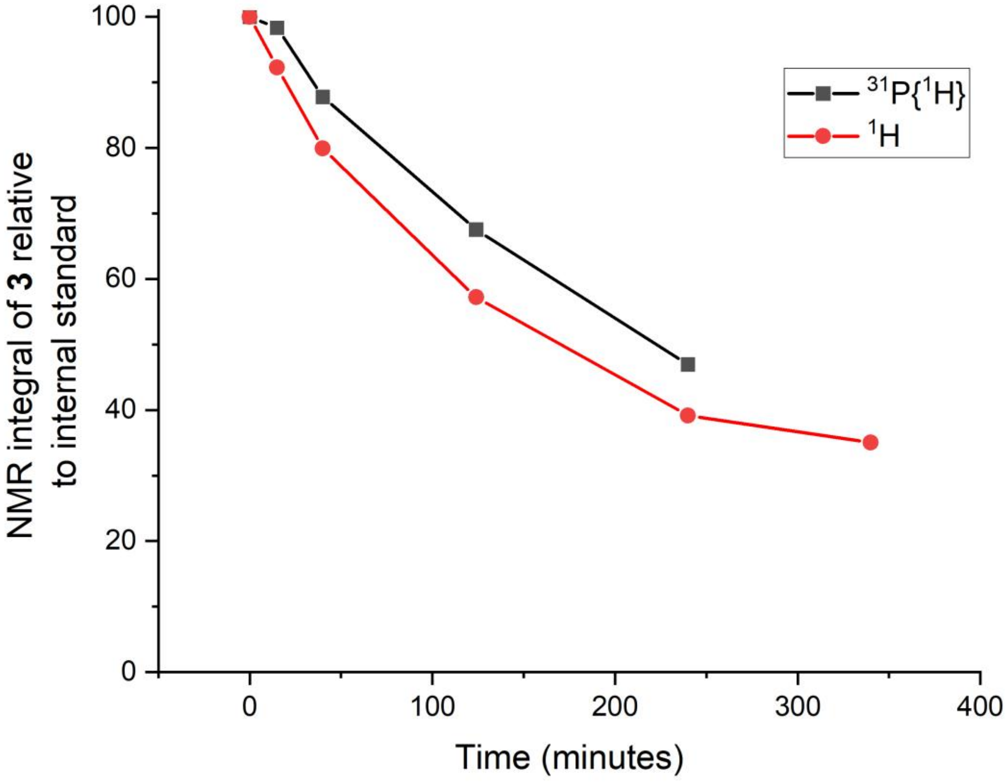

Figure S47. Consumption of **3^{Cy}** during photoreduction of a solution of **3^{Cy}** 30 equiv. pyridine in d^{8}-toluene with medium wave (302 nm) UV irradiation over time. Measured by integral of the ^{31}P {^{1}H} NMR signal relative to internal standard (PPh_3 capilliary) or to OC**H_2**CH_2C**H_2**O signal relative to internal standard (CH_2Cl_2).

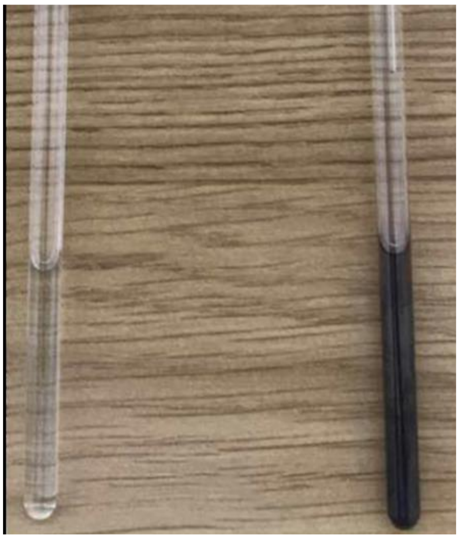

Figure S48. Photograph of a solution of **3^{Cy}** dissolved in toluene in a Young's tap NMR tube with 30 equivalents of THF (left) or pyridine (right) after 4 hours irradiation under 302 nm UV light.

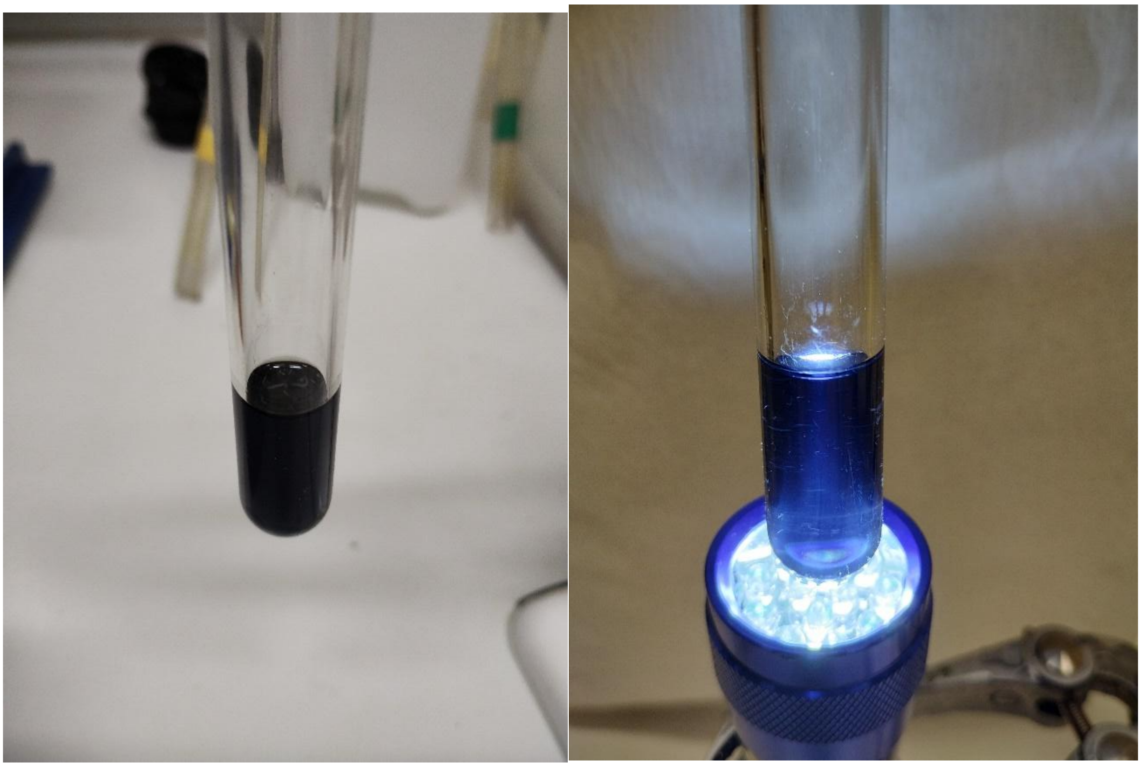

Figure S49. Photographs of a photoactivated solution of $\mathbf{3^{Cy}}$ in toluene with ~60 equiv. pyridine. Right hand photograph shows illumination under torchlight.

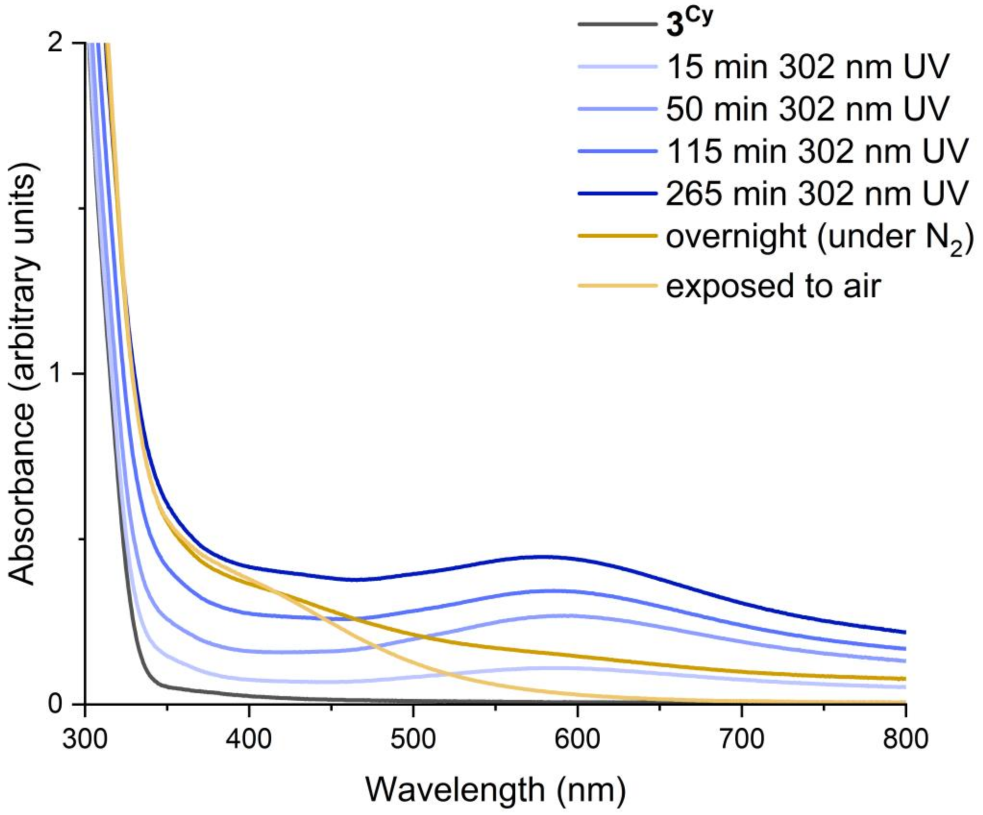

Figure S50. UV-vis spectra of $\mathbf{3}^{Cy}$ under irradiation with 302 nm light, then aged overnight under N_2 atmosphere and finally exposed to air. [$\mathbf{3}^{Cy}$] ~ 1.25 mM, [Ti] ~ 5 mM.

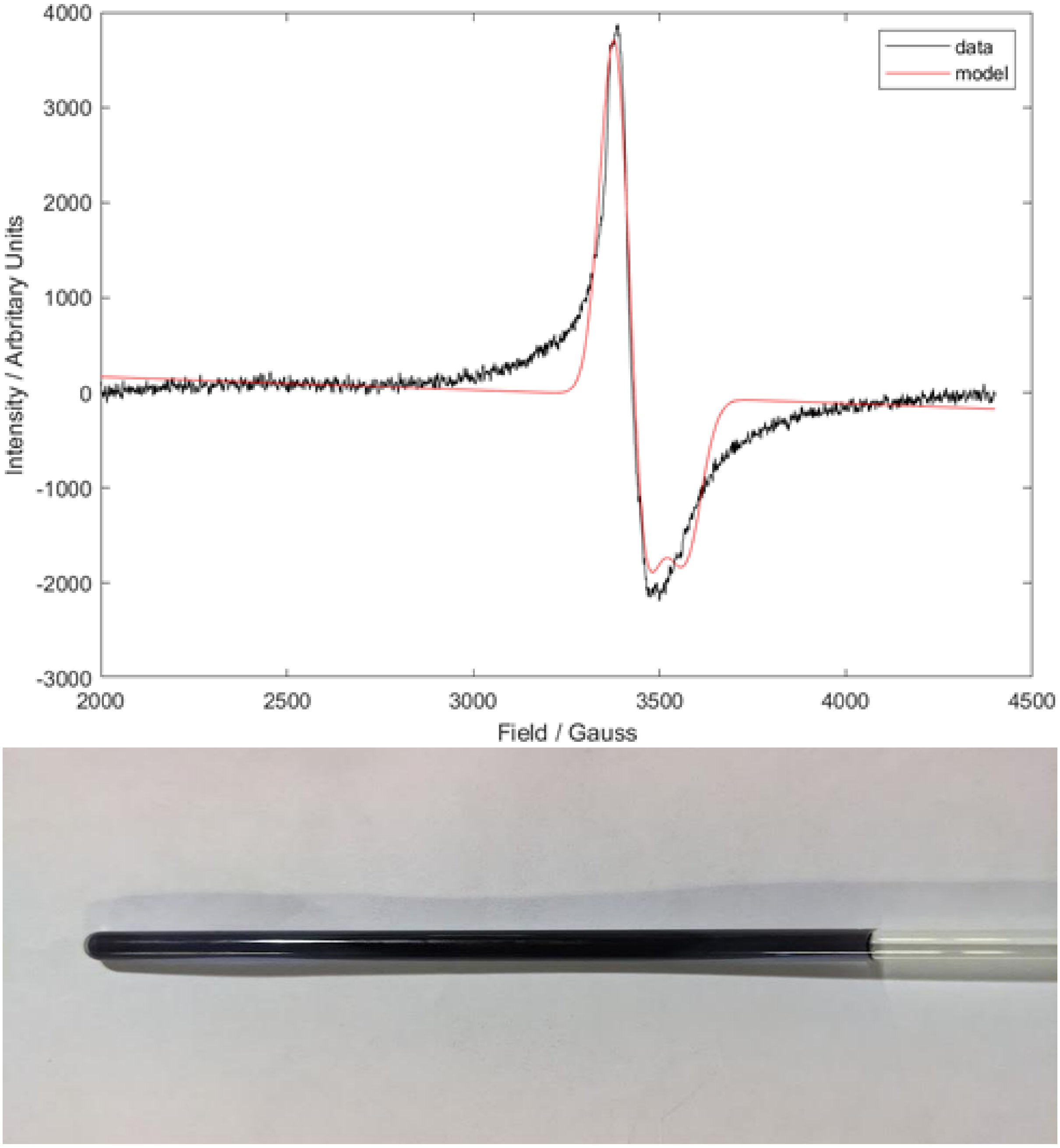

Figure S51. Top) X-band EPR spectrum of **3**^{Cy} + 30 equiv pyridine in toluene which had been irradiated with 302 nm light for 60 minutes and then frozen at 150 K. Modelling (red trace) gives values of g‖ = 1.96, g⊥ = 1.86, with gaussian line broadening of 8.6 G. Bottom) Photograph of the photoactivated solution.

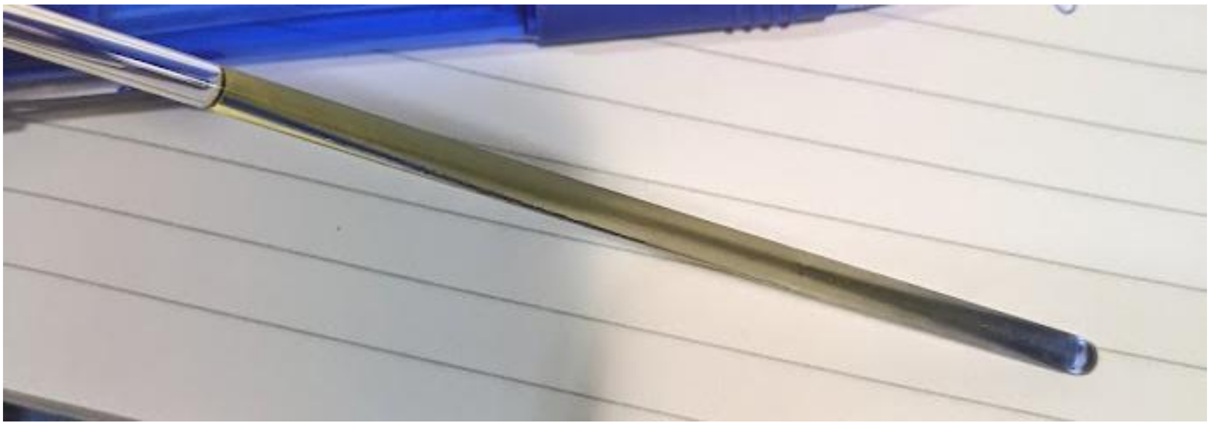

Fig S52. Photograph of photoactivated solution of **3^{Cy}** on exposure to air as blue colour changes to yellow upon reaction with oxygen. An oxidation gradient is observed from left to right as air has not fully diffused throughout the tube.

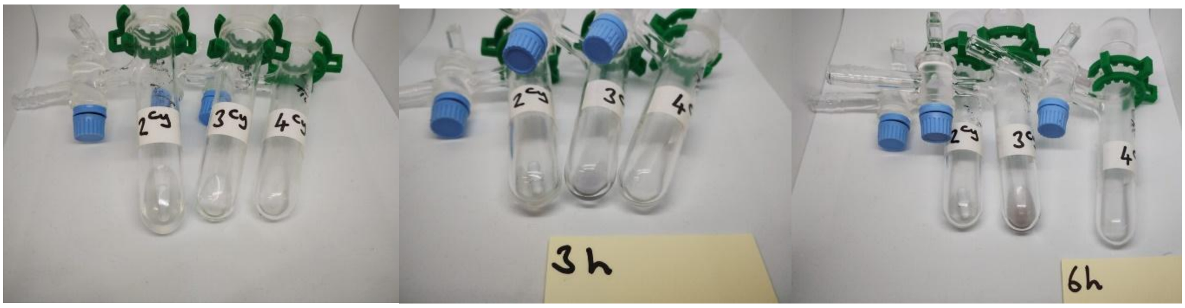

Figure S53. Photographs showing 1 mL toluene solutions containing ~5 mg **2^{Cy}**, **3^{Cy}** or **4^{Cy}** and 20μL pyridine, and after 3 and 6 hours under 302 nm irradiation. Only **3^{Cy}** undergoes colour changes.

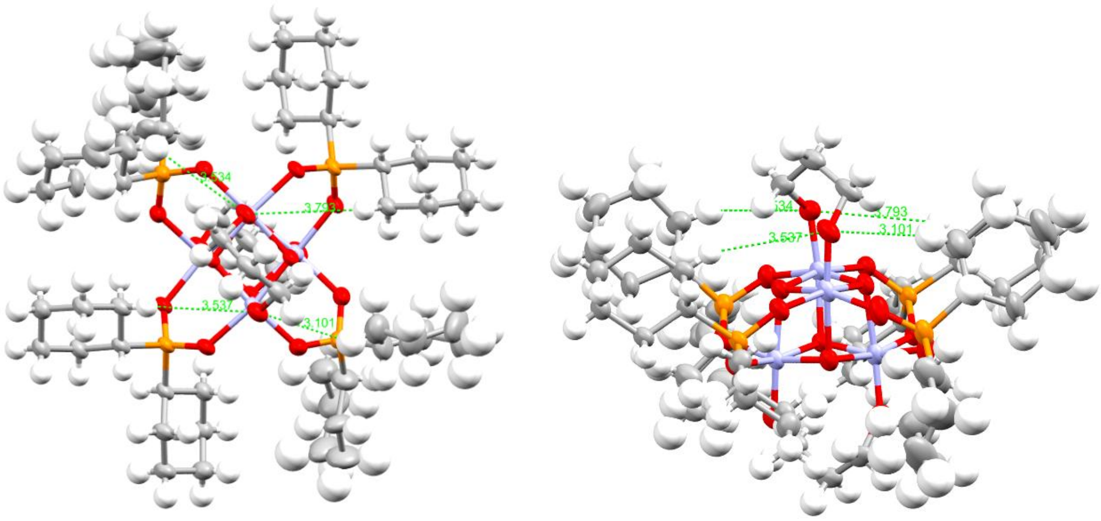

Figure S54. Single crystal structure of **3^{Cy}** showing reasonably close distances (3.1-3.8 Å) of 1,3-propane diolate oxygen with nearby α-H of cyclohexylphosphinate ligands.

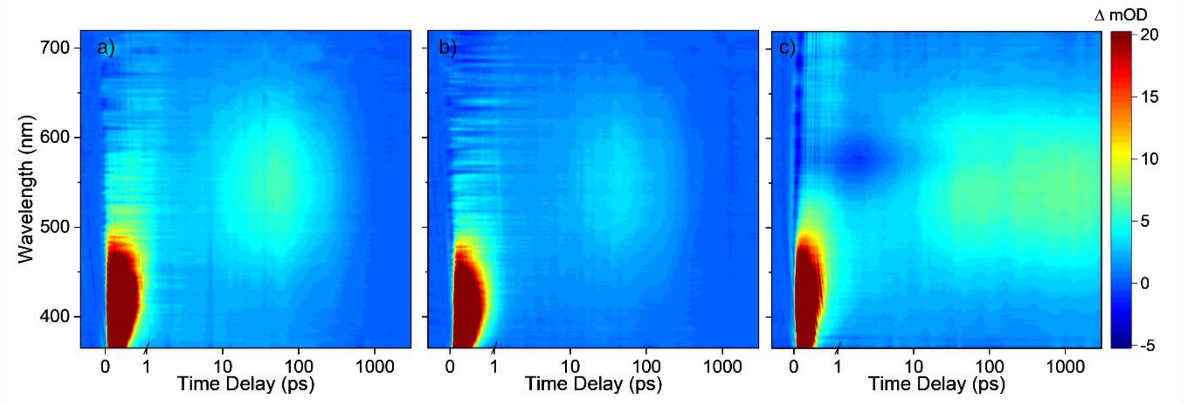

Figure S55. False colour heatmaps of the transient absorption spectra of a) **3**^{Cy} b) **2**^{Cy} solvated in toluene with 30 equivalents ^{i}PrOH, and c) **1**^{Ph} solvated in toluene with 30 equivalents of ^{i}PrOH, following photoexcitation at 350 nm. The time delay is plotted linearly up to 1 ps and then logarithmically to the maximum time delay of 3 ns.

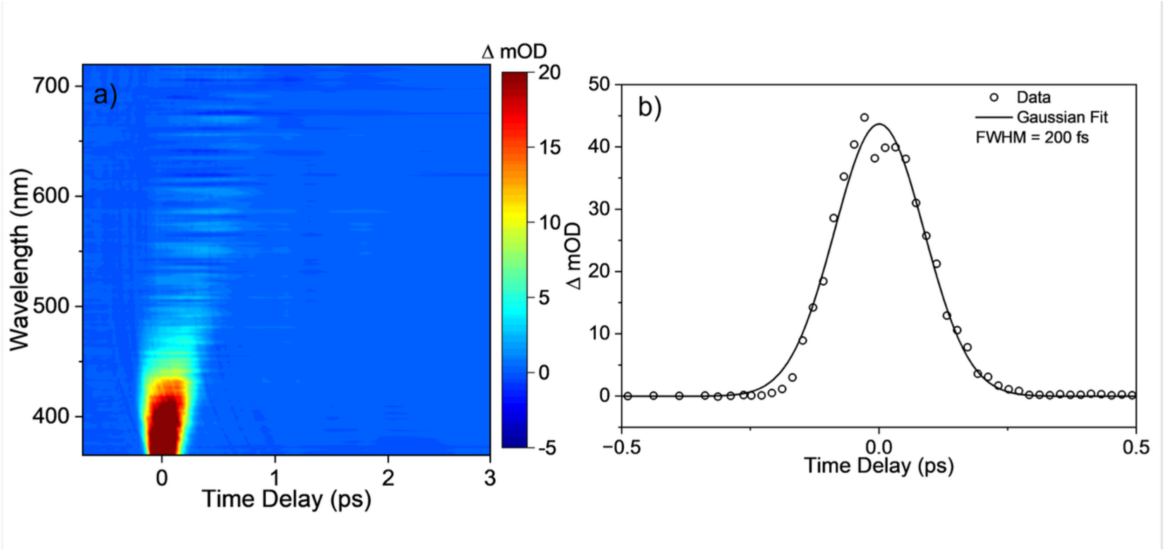

Figure S56: a) False colour heatmaps of the transient absorption spectra of toluene/pyridine solvent mixture, b) Instrument response taken at a probe wavelength of 375 nm.

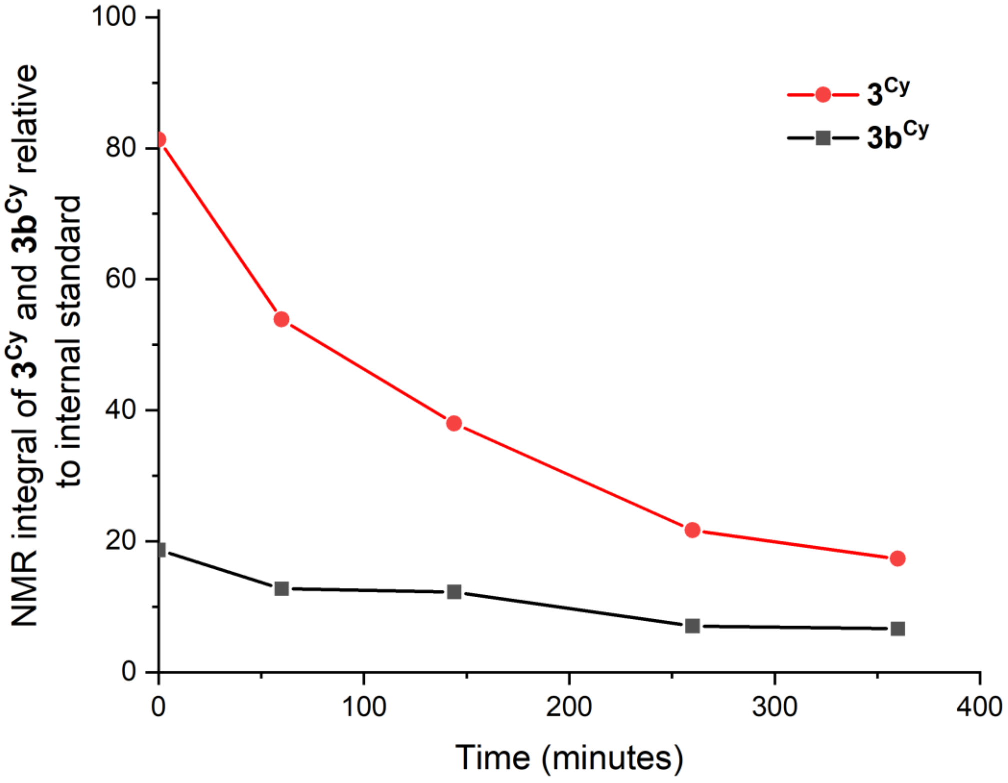

Figure S57. Consumption of **3**^{Cy} & **3b**^{Cy} during photoreduction of a solution of **3**^{Cy} + 30 equiv. ^{i}PrOH (left to equilibrate for three days before photoirradiation) in d^{8}-toluene with medium wave (302 nm) UV irradiation over time. Measured by integral of the ^{31}P NMR signals relative to internal standard (PPh_3 capilliary). Photoirradiation leads to a blue solution. Cluster photoproducts of **3**^{Cy} and **3b**^{Cy} are not observed by NMR spectroscopy, likely due to paramagnetism.

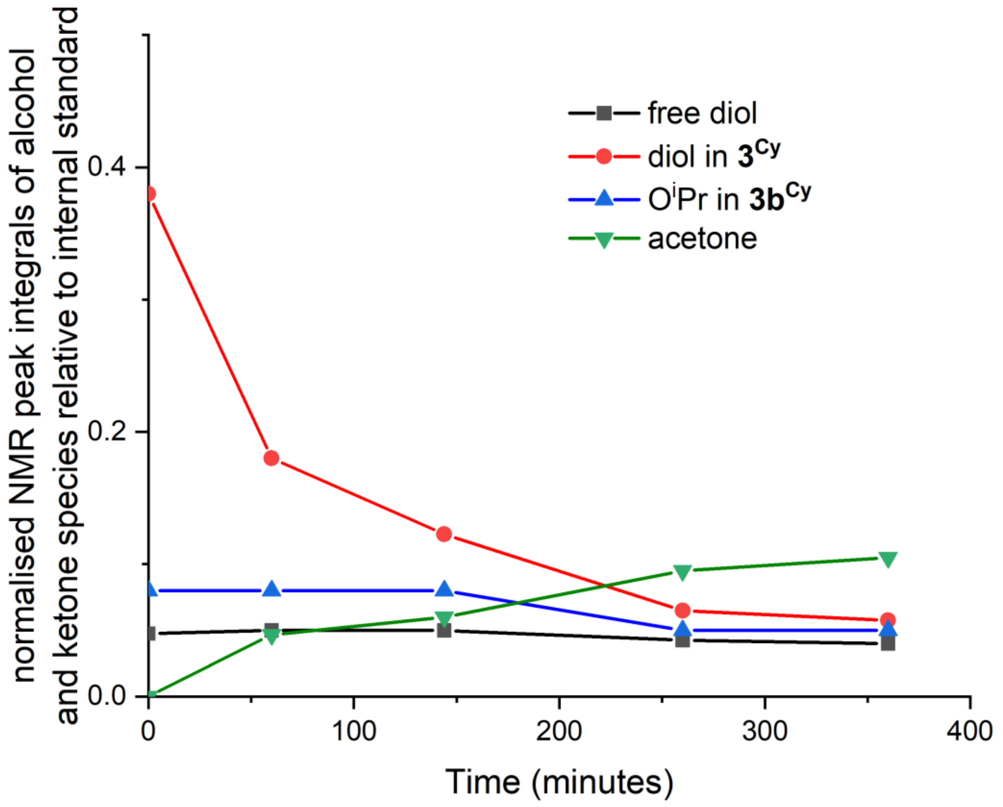

Figure S58. Consumption of **3**^{Cy} & **3b**^{Cy}, and growth of acetone during photoreduction of a solution of **3**^{Cy} + 30 equiv. ^{i}PrOH (left to equilibrate for three days before photoirradiation) in d^{8}-toluene with medium wave (302 nm) UV irradiation over time. Measured by normalised integrals of the ^{1}H NMR signals relative to internal standard (d^{8}-toluene). Direct photoproducts of **3**^{Cy} and the cluster product of **3b**^{Cy} are not observed by NMR spectroscopy, likely due to paramagnetism.

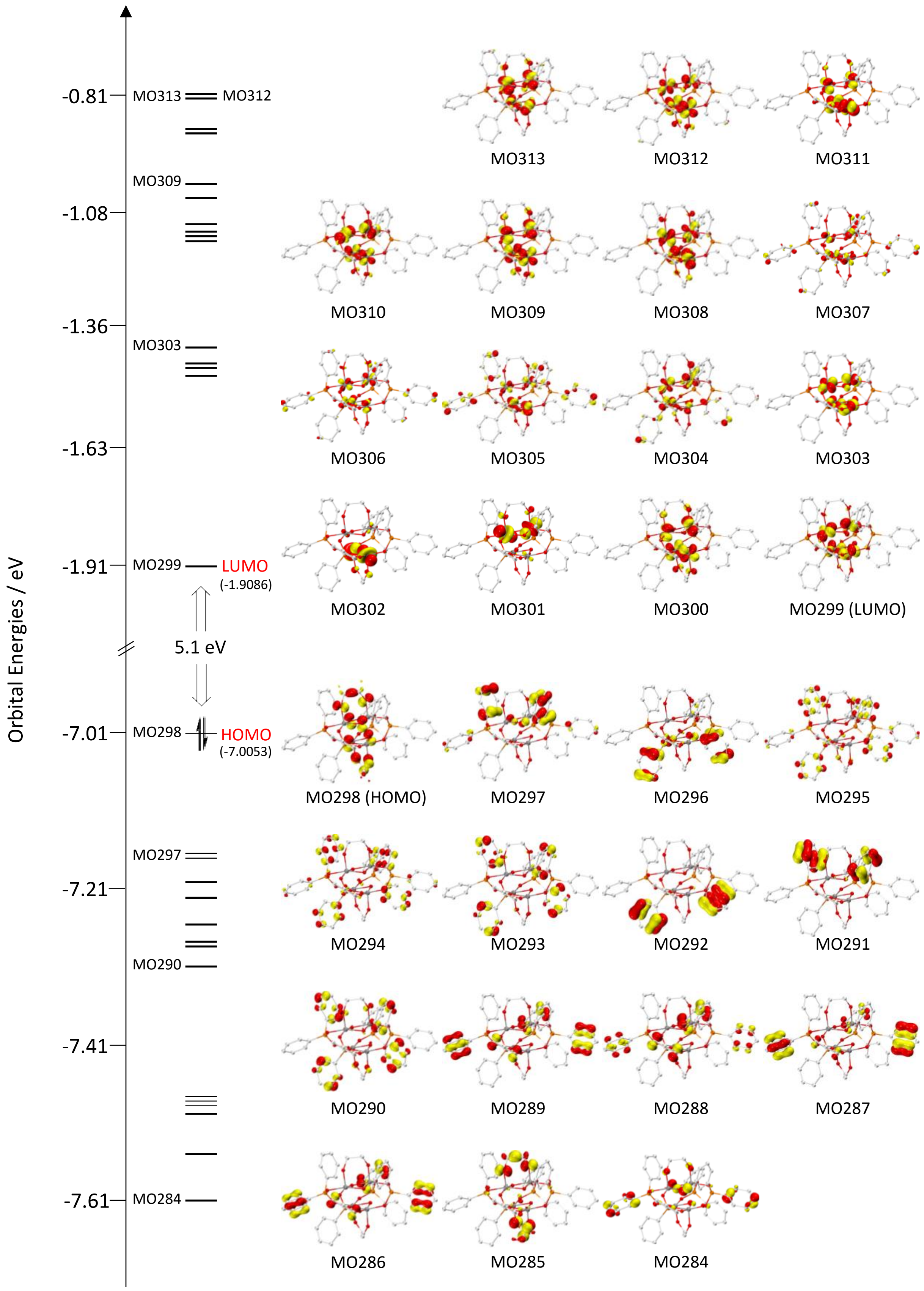

Figure S59. Canonical Kohn-Sham molecular orbital diagram (PBE/TZVP//PBE/def2-SVP) of **2**$^{\mathbf{Ph}}$.

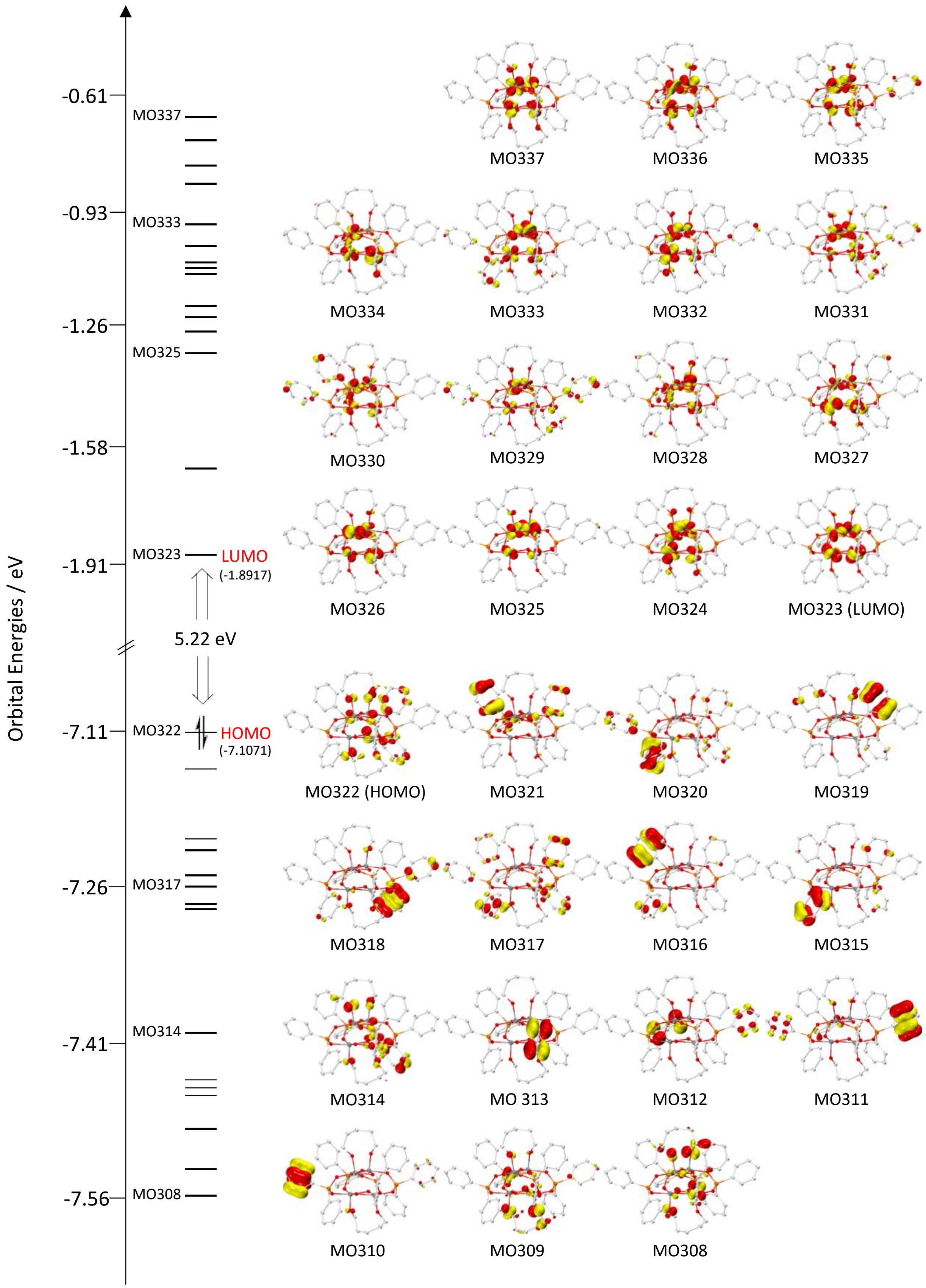

Figure S60. Canonical Kohn-Sham molecular orbital diagram (PBE/TZVP//PBE/def2-SVP) of **5**$^{\mathbf{Ph}}$.

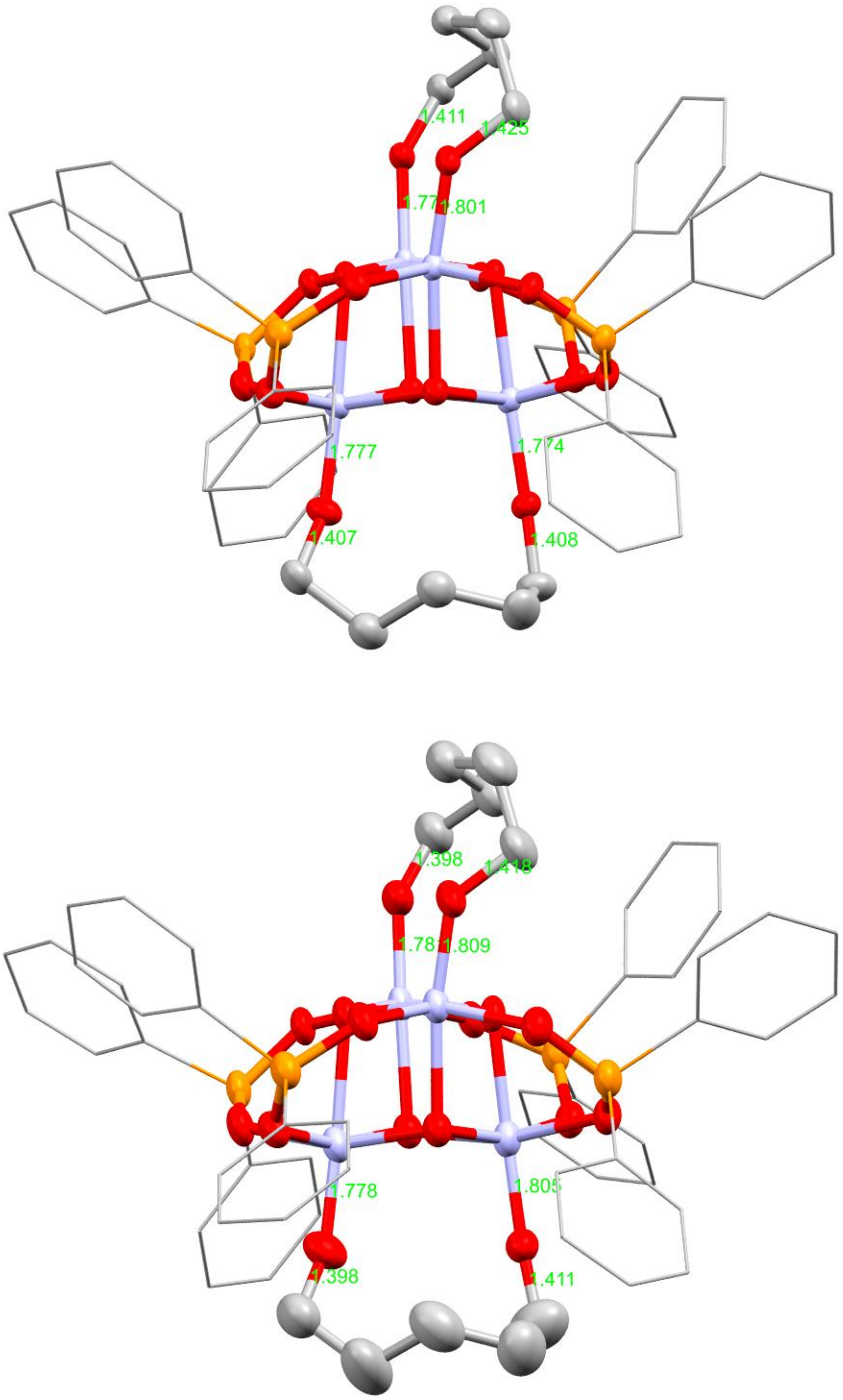

Figure S61. Single crystal structure of $\mathbf{5^{Ph}}$ before and after irradiation with 305 nm light (55 mW laser power, 3 s irradiation) including relevant bond distances. No significant changes were observed. Toluene molecules and H-atoms omitted, and Ph groups displayed as wire frame for clarity.

Supporting note S1. Photoreaction of pyridine and CH_2Cl_2 under 302 nm irradiation

3^{Ph} is insoluble in most common organic solvents (toluene, fluorobenzene, difluorobenzene, THF and pyridine all tested) with the exception of CH_2Cl_2. This restricts photochemical study to CH_2Cl_2 as solvent. In studies in which **3^{Ph}** is dissolved in CH_2Cl_2 + 30 equiv. pyridine and irradiated with 302 nm UV light spontaneous crystallisation of yellow 1,1′-methyl-enedipyridinium dichloride ([Py-CH_2-Py]Cl_2) occurs, which is identified by X-ray crystallography (Fig. S22).[20] This organic product is also produced photochemically in the absence of cluster. Whilst pyridine and CH_2Cl_2 are unreactive under ambient conditions, this salt has been previously synthesised by the application of high pressures (10,000 bar) to a mixture of the solvents.[21] It appears that through use of 302 nm UV irradiation (note that the absorption onset for pyridine is approximately 310 nm) this process becomes accessible under 1 atm. To the best of our knowledge this photochemical organic transformation has not previously been reported and could perhaps be useful for the activation of unreactive alkyl-chloride groups. It is noteworthy that formation of the related Br salt ([Py-CH_2-Py]Br_2 is known to proceed straightforwardly from the reaction of CH_2Br_2 and pyridine at 70° deg and 1 bar,[22] and other pyridinium chloride salts may be prepared in simple heating processes.[23]

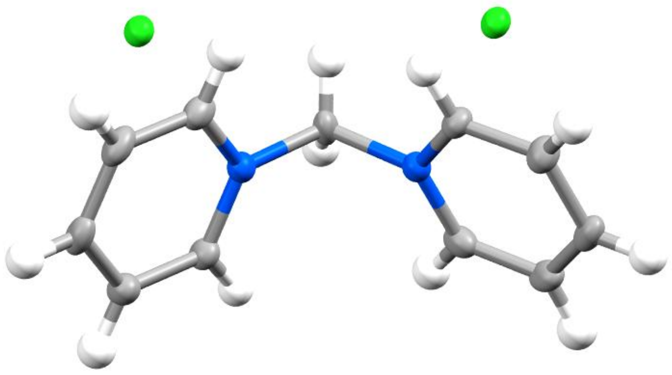

Figure SN1. X-ray crystal structure of 1,1′-methyl-enedipyridinium dichloride, ellipsoids shown with 50% probability.

X-ray Crystallography.

Data was collected using a Rigaku-Agilent Synergy diffractometer equipped with a HyPix-6000HE HPC detector, with Cu-Kα radiation, at 100 K. All structures were solved ab initio using Superflip[24] then refined with Crystals.[25] Crystallographic data have been deposited with the CCDC.

Compound **2^{Ph}**

Only small, weakly diffracting crystals of this compound could be prepared. X-ray crystallography provided confirmation of the expected structure, but the data is of lower accuracy for comparing bond length and angles.

Compound **3^{Cy}**

One of the cyclohexyl groups displays disorder and was modelled over two positions and restrained to maintain sensible geometries.

Upon inspection of the Fourier map residual electron density was located in a solvent accessible void. This is most likely attributed to a pentane molecule disordered over a symmetry position and with partial occupancy (e.g. occupancy = 20%). As such the SQUEEZE algorithm was applied using PLATON.

Compound $\mathbf{3}^{\mathbf{Ph}}$.

Crystals of compound $\mathbf{3}^{\mathbf{Ph}}$ exhibited inherent twinning. Data refinement was conducted to look for two twin components, resulting in two components of ~50% occupancy each, which could be solved separately to give the same structure.

Table S4. Crystallographic Data.

Compound	$\mathbf{2}^{Cy}$	$\mathbf{2}^{Ph}$	$\mathbf{3}^{Cy}$	$\mathbf{3}^{Ph}$	$\mathbf{4}^{Cy}$	$\mathbf{4}^{Ph}$	$\mathbf{5}^{Ph}$
CCDC number	2468340	2468341	2468342	2468343	2468344	2468345	2468346
X-ray source	Cu Kα	Cu Kα	Cu Kα	Cu Kα	Cu Kα	Synchrotron 0.6889 Å	Cu Kα
Formula	$C_{55.5}H_{102}O_{16}P_4Ti_4$	$C_{53.79}H_{49.79}Cl_{5.38}O_{16}P_4Ti_4$	$C_{54}H_{100}O_{16}P_4Ti_4$	$C_{54}H_{52}O_{16}P_4Ti_4$	$C_{56}H_{104}O_{16}P_4Ti_4$	$C_{70}H_{72}O_{16}P_4Ti_4$	$C_{68.5}H_{72}O_{16}P_4Ti_4$
M	1340.91	1458.46	1320.87	1272.49	1348.93	1484.83	1466.81
Crystal System	triclinic	triclinic	monoclinic	triclinic	Monoclinic	Triclinic	Triclinic
Space Group	P -1	P -1	P 2/c	P -1	I2/a	P -1	P -1
T [K]	100 K	100 K	100 K	100 K	100 K	100 K	100 K
a [Å]	10.9490(3)	9.6817(8)	10.9435(2)	14.8777(2)	21.9525(18)	12.2976(8)	12.8917(9)
b [Å]	16.7276(6)	14.9038(8)	15.1830(2)	14.9255(2)	15.0452(13)	15.6957(9)	16.3970(2)
c [Å]	19.3212(8)	21.3452(17)	21.4122(3)	16.20040(10)	21.236(2)	19.4240(11)	17.2133(12)
α [deg]	71.619(4)	93.685(7)	90	63.9439(13)	90	69.5670(13)	93.84(2)
β [deg]	79.491(3)	91.421(8)	94.9865(14)	88.8920(13)	110.585(10)	76.4570(13)	92.043(13)
γ [deg]	73.873(3)	90.050(6)	90	60.8791(18)	90	80.8740(13)	109.294(9)
V [Å]	3208.5(2)	3072.66(4)	3544.28(9)	2735.26(9)	6566.0(11)	3403.47(3)	3416.4(6)
Z	2	2	2	2	4	2	2
θ range [deg]	2.423 – 80.603	2.971 – 79.119	2.910 - 80.175	3.134 - 79.882	3.641 – 79.856	2.21 – 68.376	2.868 – 86.379
Reflns collected	59081	34953	53841	116211	24972	102784	71565
R int	0.099	0.245	0.046	0.081	0.082	0.109	0.054
No. of data/restr/param	12915/238/776	12089/252/758	7605/216/ 408	10923/0/703	6584/824/564	27495/242/902	13827/74/838
R1 [I>2σ(I)]	0.0839	0.1690	0.0484	0.0485	0.0949	0.0595	0.0511
wR2 [all data]	0.2386	0.4642	0.1430	0.1379	0.3226	0.1435	0.1412
GoF	0.967	1.4950	0.9915	0.9838	1.075	0.792	1.001
Largest diff. pk and hole [eÅ3]	-0.66 – 0.79	-2.57 – 2.78	-0.60 – 1.14	-0.79 – 1.69	-0.67 – 0.74	-1.70 – 1.86	-0.66 – 0.90

Computational data

Table S5. Cartesian coordinates of optimised **2^{Ph}** (PBE/def2-SVP) in Å (n_{img} = 0).

Atom	X	Y	Z
Ti	-0.00256400	-1.43264100	1.21367100
Ti	-1.43211900	0.00284800	-1.21373700
Ti	0.00356300	1.43302000	1.21323300
Ti	1.43354100	-0.00336600	-1.21355900
P	-2.95739100	-2.22132100	0.38912200
P	-2.22134500	2.95763700	-0.38929200
P	2.95819300	2.22125200	0.38899600
P	2.22140900	-2.95797100	-0.38901700
O	0.04146900	1.25688600	-0.95538800
O	-1.25654800	0.04096500	0.95488700
O	-0.04008100	-1.25732400	-0.95484100
O	1.25767800	-0.04061500	0.95509500
O	-0.09706300	-1.43792300	3.00712800
O	0.09758300	1.43886600	3.00678900
O	-1.43751600	0.09711600	-3.00726200
O	1.43920600	-0.09736300	-3.00703800
O	-1.58068100	-2.72808400	0.92821900
O	-2.91260900	-1.28456900	-0.87322800
O	-2.72769100	1.58072800	-0.92829400
O	-1.28414600	2.91339200	0.87271300
O	1.58122200	2.72867600	0.92694900
O	2.91422200	1.28403300	-0.87299200
O	2.72892700	-1.58121700	-0.92744300
O	1.28470200	-2.91343000	0.87335500
C	-0.40875100	-0.64750900	4.11269900
C	0.40850400	0.64846500	4.11273400
C	0.64904500	-0.40676700	-4.11349500
C	-0.64700100	0.41041800	-4.11238000
C	-3.83430700	-1.33924300	1.71305400
C	-3.90707700	-1.90804700	2.99949500
C	-4.50501400	-1.18764200	4.04365300

C	-5.02225800	0.09781200	3.80602200
C	-4.94180900	0.66519800	2.52377700
C	-4.34899500	-0.05084900	1.47417500
C	-3.89105000	-3.70056100	-0.09246600
C	-3.48970100	-4.96721200	0.37515700
C	-4.19516400	-6.11279000	-0.02347000
C	-5.29669800	-5.99542500	-0.88676900
C	-5.69367100	-4.73175200	-1.35646400
C	-4.99259300	-3.58335500	-0.96297200
C	-3.70097100	3.89045800	0.09266000
C	-4.96717300	3.48992300	-0.37689300
C	-6.11314700	4.19462900	0.02190600
C	-5.99663400	5.29459300	0.88732600
C	-4.73342200	5.69071600	1.35898500
C	-3.58462000	4.99038500	0.96533000
C	-1.33998500	3.83478000	-1.71355800
C	-1.90938700	3.90791300	-2.99969000
C	-1.18942300	4.50602200	-4.04404800
C	0.09617900	5.02312800	-3.80689600
C	0.66412000	4.94237700	-2.52491500
C	-0.05148000	4.34936300	-1.47513400
C	3.89249300	3.70017200	-0.09225400
C	3.49168000	4.96674700	0.37599500
C	4.19752200	6.11222000	-0.02214800
C	5.29897000	5.99483000	-0.88555700
C	5.69542200	4.73122700	-1.35587500
C	4.99388800	3.58293600	-0.96294700
C	3.83349800	1.33938200	1.71412400
C	3.90418400	1.90825200	3.00060200
C	4.50082100	1.18805200	4.04564900
C	5.01880800	-0.09725100	3.80879100
C	4.94029600	-0.66472700	2.52645500
C	4.34876700	0.05111000	1.47600200
C	1.33881300	-3.83355500	-1.71350300

C	0.05037200	-4.34821200	-1.47479000
C	-0.66597200	-4.94002800	-2.52474600
C	-0.09889500	-5.01936500	-3.80720200
C	1.18650600	-4.50191600	-4.04468300
C	1.90726800	-3.90506800	-3.00013300
C	3.70030800	-3.89242000	0.09203100
C	4.96697200	-3.49145900	-0.37589800
C	6.11239500	-4.19743300	0.02223000
C	5.99487800	-5.29906500	0.88538300
C	4.73120600	-5.69561400	1.35543400
C	3.58295200	-4.99404000	0.96241800
H	-1.49593800	-0.39708800	4.09943800
H	-0.19738100	-1.21022500	5.05280700
H	0.19663500	1.21139800	5.05260400
H	1.49564900	0.39789700	4.10019900
H	1.21207500	-0.19352200	-5.05299700
H	0.39858100	-1.49396200	-4.10253800
H	-1.20963000	0.20028300	-5.05281900
H	-0.39667100	1.49760300	-4.09759700
H	-3.47572000	-2.90330200	3.18787100
H	-4.56038800	-1.62732500	5.05130000
H	-5.48278800	0.66402100	4.63026000
H	-5.32455300	1.68091400	2.33856500
H	-4.23097900	0.40221700	0.47925600
H	-2.61330200	-5.04181700	1.03655800
H	-3.87998300	-7.10358200	0.33778300
H	-5.84758100	-6.89548200	-1.20012300
H	-6.55131800	-4.64254300	-2.04048500
H	-5.28262300	-2.59169800	-1.34194900
H	-5.04113100	2.61462300	-1.03981600
H	-7.10359100	3.88004500	-0.34081500
H	-6.89700700	5.84486500	1.20084300
H	-4.64489900	6.54706100	2.04472300
H	-2.59333700	5.27961300	1.34589000

H	-2.90480400	3.47677600	-3.18770300
H	-1.62958600	4.56169400	-5.05146900
H	0.66202700	5.48383300	-4.63128400
H	1.67992100	5.32507400	-2.34006200
H	0.40197700	4.23118400	-0.48040600
H	2.61530600	5.04132300	1.03742200
H	3.88271200	7.10296600	0.33955000
H	5.85017400	6.89481700	-1.19854400
H	5.28339100	2.59135400	-1.34251900
H	3.47206300	2.90331100	3.18827700
H	4.55457200	1.62776100	5.05337200
H	5.47834800	-0.66329800	4.63369200
H	5.32352500	-1.68036900	2.34184800
H	4.23210400	-0.40203400	0.48094100
H	-0.40256900	-4.23072500	-0.47973000
H	-1.68169800	-5.32280300	-2.33964700
H	-0.66533500	-5.47910500	-4.63172100
H	1.62586700	-4.55625900	-5.05252700
H	2.90242800	-3.47340600	-3.18832200
H	5.04166600	-2.61493300	-1.03711800
H	7.10319700	-3.88256000	-0.33926700
H	6.89482100	-5.85034300	1.19836900
H	4.64188900	-6.55331800	2.03936700
H	2.59131400	-5.28372700	1.34170300
H	6.55293800	4.64201700	-2.04006000

Table S5. Cartesian coordinates of optimised **5^{Ph}** (PBE/def2-SVP) in Å (n_{img} = 0).

Atom	X	Y	Z
Ti	-1.38425600	0.50344500	-1.29369900
Ti	-0.42949100	-1.22231600	1.16976500
Ti	0.52667700	1.48517800	0.98576900
Ti	1.32547400	-0.48466600	-1.32671700
P	-3.20767000	-1.90922200	-0.21587800
P	-2.02298700	3.17180500	0.29261100

P	3.15585100	2.04547600	-0.72370000
P	2.08275900	-2.98150800	0.49329100
O	-0.43587800	-1.17416100	-0.99736000
O	-1.14642200	0.53396000	0.85515200
O	1.24048500	-0.28651300	0.80126200
O	0.37472800	1.20104500	-1.20159600
O	-2.19371500	-2.15144000	0.95229000
O	-3.14155500	-0.51776700	-0.92279700
O	-2.29848100	2.26477300	-0.95385600
O	-0.52641000	3.20978800	0.77520000
O	2.26518400	2.37076500	0.52305500
O	3.05390300	0.58862500	-1.29300200
O	2.29089600	-2.21423100	-0.85242200
O	0.62657200	-2.95305300	1.08313800
O	-1.77202200	0.44888700	-3.03514000
O	1.39944600	-0.99625600	-3.04633800
O	0.86257200	1.57829700	2.74827700
O	-0.59637800	-1.09539100	2.94484900
C	-4.87671900	-2.02741100	0.48927500
C	-5.08930400	-2.77914500	1.66108300
C	-6.36559200	-2.81811900	2.24127800
C	-7.42833200	-2.10995800	1.65524500
C	-7.21545100	-1.35596100	0.48883200
C	-5.94003200	-1.30916900	-0.09365100
C	-2.95524300	-3.23783300	-1.43302300
C	-1.75939600	-3.98237600	-1.38531200
C	-1.49901800	-4.94087800	-2.37567500
C	-2.42821200	-5.16365700	-3.40517900
C	-3.62689500	-4.43113300	-3.44398600
C	-3.89188800	-3.46774400	-2.46004700
C	-3.06420500	2.63576500	1.67947100
C	-4.11609400	1.73010000	1.43995900
C	-4.87113500	1.24148200	2.51534900
C	-4.58475000	1.66259000	3.82486000

C	-3.53158800	2.56117700	4.06396600
C	-2.76122500	3.04154800	2.99447900
C	-2.45701600	4.86120500	-0.21500800
C	-2.75004000	5.86840700	0.72455900
C	-2.99900100	7.17846400	0.29005800
C	-2.95667200	7.48632200	-1.07968800
C	-2.67212900	6.48080000	-2.01822100
C	-2.42562300	5.16787000	-1.59062700
C	2.75126800	3.25035900	-2.01854800
C	1.63329400	4.09089400	-1.84733300
C	1.27825400	4.98388000	-2.86884300
C	2.03141300	5.03944700	-4.05265600
C	3.14516300	4.19917000	-4.22162500
C	3.50644300	3.30234400	-3.20661100
C	4.88311800	2.24508700	-0.20017500
C	5.20067900	3.16105900	0.82205700
C	6.52474600	3.27248200	1.27014400
C	7.53078400	2.47250700	0.70189600
C	7.21339200	1.55632700	-0.31464500
C	5.89014300	1.43810000	-0.76585000
C	3.26104300	-2.33002800	1.71504600
C	4.17232300	-1.32864000	1.32613900
C	5.03652400	-0.76734800	2.27727100
C	4.99289500	-1.20537200	3.61144800
C	4.08193300	-2.20332900	3.99867600
C	3.21280800	-2.76695100	3.05279100
C	2.45690600	-4.72925000	0.17864700
C	3.61033200	-5.07661700	-0.55307700
C	3.88164400	-6.42420800	-0.82606500
C	3.00363800	-7.42496000	-0.37482800
C	1.85158300	-7.07844500	0.34904800
C	1.57493900	-5.73089700	0.62779700
C	-2.53729300	-0.18491400	-4.02287000
C	-1.75797500	-1.35645600	-4.62049800

C	-0.44219500	-0.90905200	-5.27708900
C	0.69850700	-1.92791600	-5.17400900
C	1.12010900	-2.19420000	-3.72561400
C	1.93274200	1.13468000	3.54621100
C	1.56573100	0.91591100	5.01801200
C	0.85672900	-0.40371300	5.37813100
C	-0.67435500	-0.45960000	5.28188300
C	-1.25551900	-0.27990100	3.87992300
H	-4.24177400	-3.30828300	2.12217900
H	-6.53133700	-3.39829000	3.16181700
H	-8.42826100	-2.14021800	2.11454500
H	-8.04551100	-0.79167000	0.03721100
H	-5.75030000	-0.69316100	-0.98581500
H	-1.03003800	-3.78780600	-0.58560300
H	-0.55940900	-5.51368600	-2.34031500
H	-2.21933800	-5.91451700	-4.18263600
H	-4.35700400	-4.60899800	-4.24810100
H	-4.83007400	-2.89169100	-2.49261500
H	-4.29596300	1.37042800	0.41646500
H	-5.67323600	0.51087300	2.32860100
H	-5.17607600	1.27385600	4.66816100
H	-3.29302300	2.87161900	5.09273400
H	-1.89489100	3.69413500	3.18236800
H	-2.79156800	5.62797900	1.79772600
H	-3.22982600	7.96403000	1.02546500
H	-3.15257600	8.51543300	-1.41766600
H	-2.64784800	6.71921800	-3.09267800
H	-2.20838700	4.36596000	-2.31233600
H	1.03651000	4.02494600	-0.92528700
H	0.40078900	5.63551100	-2.73609400
H	1.74861400	5.74091300	-4.85271900
H	3.73274800	4.24072100	-5.15133400
H	4.37518900	2.63899400	-3.34056800
H	4.39928200	3.76469200	1.27419800

H	6.77327600	3.98263300	2.07348900
H	8.56866800	2.55980600	1.05813600
H	7.99999500	0.92257100	-0.75164500
H	5.62001700	0.70228100	-1.53846000
H	4.16097700	-0.96249400	0.28910200
H	5.73250000	0.03002600	1.97379600
H	5.66663600	-0.75885200	4.35891400
H	4.03986400	-2.53573600	5.04705000
H	2.48170700	-3.53170400	3.35734700
H	4.28521300	-4.28651100	-0.91643900
H	4.78066100	-6.69731300	-1.39931500
H	3.21861500	-8.48236200	-0.59274600
H	1.16141900	-7.86225000	0.69673100
H	0.67106200	-5.43693000	1.18245100
H	-3.49102600	-0.54186400	-3.56753600
H	-2.79477900	0.56235900	-4.81086400
H	-1.55972700	-2.06360100	-3.78893000
H	-2.40754500	-1.90174200	-5.33886300
H	-0.09750900	0.01588100	-4.77004000
H	-0.61678700	-0.64284800	-6.34131200
H	0.41164600	-2.90192300	-5.63054600
H	1.57681600	-1.55469200	-5.74300500
H	2.02015500	-2.85099700	-3.69497600
H	0.31293100	-2.73314300	-3.17645500
H	2.74520300	1.89571600	3.47790200
H	2.33754600	0.19174800	3.11113400
H	2.53062800	0.94116400	5.56938000
H	0.98636900	1.79399100	5.38162700
H	1.12598000	-0.66411600	6.42419600
H	1.27935200	-1.21348800	4.74527000
H	-1.13084700	0.31632900	5.93686300
H	-1.00551400	-1.44152400	5.68473100
H	-2.34227800	-0.52739300	3.87809400
H	-1.16258200	0.77624400	3.54737900

References

1. Krämer, T.; Tuna, F.; Pike, S. D., Photo-redox reactivity of titanium-oxo clusters: mechanistic insight into a two-electron intramolecular process, and structural characterisation of mixed-valent Ti(iii)/Ti(iv) products. *Chem. Sci.* **2019,** *10* (28), 6886-6898.
2. Brown, S. E.; Warren, M. R.; Kubicki, D. J.; Fitzpatrick, A.; Pike, S. D., Photoinitiated Single-Crystal to Single-Crystal Redox Transformations of Titanium-Oxo Clusters. *J. Am. Chem. Soc.* **2024,** *146* (25), 17325-17333.
3. Woolley, J. M.; Staniforth, M.; Horbury, M. D.; Richings, G. W.; Wills, M.; Stavros, V. G., Unravelling the Photoprotection Properties of Mycosporine Amino Acid Motifs. *J. Phys. Chem. Lett.* **2018,** *9* (11), 3043-3048.
4. Grubb, M. P.; Orr-Ewing, A. J.; Ashfold, M. N. R., KOALA: A program for the processing and decomposition of transient spectra. *Rev. Sci. Instrum.* **2014,** *85* (6).
5. Frisch, M. J.; Trucks, G. W.; Schlegel, H. B.; Scuseria, G. E.; Robb, M. A.; Cheeseman, J. R.; Scalmani, G.; Barone, V.; Petersson, G. A.; Nakatsuji, H.; Li, X.; Caricato, M.; Marenich, A. V.; Bloino, J.; Janesko, B. G.; Gomperts, R.; Mennucci, B.; Hratchian, H. P.; Ortiz, J. V.; Izmaylov, A. F.; Sonnenberg, J. L.; Williams; Ding, F.; Lipparini, F.; Egidi, F.; Goings, J.; Peng, B.; Petrone, A.; Henderson, T.; Ranasinghe, D.; Zakrzewski, V. G.; Gao, J.; Rega, N.; Zheng, G.; Liang, W.; Hada, M.; Ehara, M.; Toyota, K.; Fukuda, R.; Hasegawa, J.; Ishida, M.; Nakajima, T.; Honda, Y.; Kitao, O.; Nakai, H.; Vreven, T.; Throssell, K.; Montgomery Jr., J. A.; Peralta, J. E.; Ogliaro, F.; Bearpark, M. J.; Heyd, J. J.; Brothers, E. N.; Kudin, K. N.; Staroverov, V. N.; Keith, T. A.; Kobayashi, R.; Normand, J.; Raghavachari, K.; Rendell, A. P.; Burant, J. C.; Iyengar, S. S.; Tomasi, J.; Cossi, M.; Millam, J. M.; Klene, M.; Adamo, C.; Cammi, R.; Ochterski, J. W.; Martin, R. L.; Morokuma, K.; Farkas, O.; Foresman, J. B.; Fox, D. J. *Gaussian 16 Rev. C.01*, Wallingford, CT, 2016.
6. Perdew, J. P.; Burke, K.; Ernzerhof, M., Generalized Gradient Approximation Made Simple. *Phys. Rev. Lett.* **1996,** *77* (18), 3865-3868.
7. Weigend, F.; Ahlrichs, R., Balanced basis sets of split valence, triple zeta valence and quadruple zeta valence quality for H to Rn: Design and assessment of accuracy. *Phys. Chem. Chem. Phys.* **2005,** *7* (18), 3297-3305.
8. Grimme, S.; Ehrlich, S.; Goerigk, L., Effect of the damping function in dispersion corrected density functional theory. *J. Comput. Chem.* **2011,** *32* (7), 1456-1465.
9. Grimme, S.; Antony, J.; Ehrlich, S.; Krieg, H., A consistent and accurate ab initio parametrization of density functional dispersion correction (DFT-D) for the 94 elements H-Pu. *J. Chem. Phys.* **2010,** *132* (15).
10. Marenich, A. V.; Cramer, C. J.; Truhlar, D. G., Universal Solvation Model Based on Solute Electron Density and on a Continuum Model of the Solvent Defined by the Bulk Dielectric Constant and Atomic Surface Tensions. *J. Phys. Chem. B* **2009,** *113* (18), 6378-6396.
11. Schäfer, A.; Huber, C.; Ahlrichs, R., Fully optimized contracted Gaussian basis sets of triple zeta valence quality for atoms Li to Kr. *J. Chem. Phys.* **1994,** *100* (8), 5829-5835.
12. Adamo, C.; Barone, V., Toward reliable density functional methods without adjustable parameters: The PBE0 model. *J. Chem. Phys.* **1999,** *110* (13), 6158-6170.
13. Hirata, S.; Head-Gordon, M., Time-dependent density functional theory within the Tamm–Dancoff approximation. *Chem. Phys. Lett.* **1999,** *314* (3), 291-299.
14. Stratmann, R. E.; Scuseria, G. E.; Frisch, M. J., An efficient implementation of time-dependent density-functional theory for the calculation of excitation energies of large molecules. *J. Chem. Phys.* **1998,** *109* (19), 8218-8224.
15. Casida, M. E., Time-dependent density-functional theory for molecules and molecular solids. *J. Mol. Struct.: THEOCHEM* **2009,** *914* (1), 3-18.
16. Martin, R. L., Natural transition orbitals. *J. Chem. Phys.* **2003,** *118* (11), 4775-4777.
17. Humphrey, W.; Dalke, A.; Schulten, K., VMD: Visual molecular dynamics. *J. Mol. Graph.* **1996,** *14* (1), 33-38.

18. Lu, T.; Chen, F., Multiwfn: A multifunctional wavefunction analyzer. *J. Comput. Chem.* **2012,** *33* (5), 580-592.
19. Lin, C.-G.; Hutin, M.; Busche, C.; Bell, N. L.; Long, D.-L.; Cronin, L., Elucidating the paramagnetic interactions of an inorganic–organic hybrid radical-functionalized Mn-Anderson cluster. *Dalton Trans.* **2021,** *50* (7), 2350-2353.
20. Fu, W.-Z.; Wang, W.-J.; Niu, Y.-Y.; Ng, S. W., 1,1'-Methylenedipyridinium dichloride monohydrate. *Acta Cryst. Sect. E* **2010,** *66* (5), o1211.
21. Almarzoqi, B.; George, A. V.; Isaacs, N. S., The Quarternisation of Tertiary Amines with Dihalomethane. *Tetrahedron* **1986,** *42* (2), 601-607.
22. Zhang, Z.-F., Crystal structure of 1,1'-methylenedipyridinium dibromide monohydrate, (C11H12N2)Br2) H2O. *Z. Kristallogr. - New Cryst. Struct.* **2011,** *226* (4), 553-554.
23. Zhao, S.; He, M.; Guo, Z.; Zhou, N.; Wang, D.; Li, J.; Zhang, L., [HyEtPy]Cl–H2O: an efficient and versatile solvent system for the DABCO-catalyzed Morita–Baylis–Hillman reaction. *RSC Adv.* **2015,** *5* (41), 32839-32845.
24. Palatinus, L.; Chapuis, G., SUPERFLIP - a computer program for the solution of crystal structures by charge flipping in arbitrary dimensions. *J. Appl. Crystallogr.* **2007,** *40* (4), 786-790.
25. Betteridge, P. W.; Carruthers, J. R.; Cooper, R. I.; Prout, K.; Watkin, D. J., CRYSTALS version 12: software for guided crystal structure analysis. *J. Appl. Crystallogr.* **2003,** *36* (6), 1487.
